# Small area variation in severe, moderate, and mild anemia among women and children: A multilevel analysis of 707 districts in India

**DOI:** 10.3389/fpubh.2022.945970

**Published:** 2022-09-20

**Authors:** Sunil Rajpal, Akhil Kumar, Md Juel Rana, Rockli Kim, S. V. Subramanian

**Affiliations:** ^1^Interdisciplinary Program in Precision Public Health, Department of Public Health Sciences, Graduate School of Korea University, Seoul, South Korea; ^2^Department of Economics, FLAME University, Pune, India; ^3^Turner Fenton Secondary School, Brampton, ON, Canada; ^4^Korea University Research and Business Foundation, Seoul, South Korea; ^5^Division of Health Policy and Management, College of Health Science, Korea University, Seoul, South Korea; ^6^Harvard Center for Population and Development Studies, Cambridge, MA, United States; ^7^Department of Social and Behavioral Sciences, Harvard T.H. Chan School of Public Health, Boston, MA, United States

**Keywords:** anemia, small area variation, multilevel modeling, nutrition, India

## Abstract

India is home to the highest global number of women and children suffering from anemia, with one in every two women impacted. India's current strategy for targeting areas with a high anemia burden is based on district-level averages, yet this fails to capture the substantial small area variation in micro-geographical (small area) units such as villages. We conducted statistical and econometric analyses to quantify the extent of small area variation in the three grades of anemia (severe, moderate, and mild) among women and children across 36 states/union territories and 707 districts of India. We utilized data from the fifth round of the National Family Health Survey conducted in 2019–21. The final analytic sample for analyses was 183,883 children aged 6–59 months and 690,153 women aged 15–49 years. The primary outcome variable for the analysis was the three anemia grades among women and children. We adopted a three-level and four-level logistic regression model to compute variance partitioning of anemia among women and children. We also computed precision-weighted prevalence estimates of women and childhood anemia across 707 districts and within-district, between-cluster variation using standard deviation (SD). For severe anemia among women, small area (villages or urban blocks) account for highest share (46.1%; Var: 0.494; SE: 0.150) in total variation followed by states (39.4%; Var: 0.422; SE: 0.134) and districts (12.8%; Var: 0.156; SE: 0.012). Similarly, clusters account for the highest share in the variation in severe (61.3%; Var: 0.899; SE: 0.069) and moderate (46.4%: Var: 0.398; SE: 0.011) anemia among children. For mild and moderate anemia among women, however, states were the highest source of variation. Additionally, we found a high and positive correlation between mean prevalence and inter-cluster SD of moderate and severe anemia among women and children. In contrast, the correlation was weaker for mild anemia among women (*r* = 0.61) and children (0.66). In this analysis, we are positing the critical importance of small area variation within districts when designing strategies for targeting high burden areas for anemia interventions.

## Introduction

Anemia (low hemoglobin concentration in the blood) continues to be a major public health concern affecting one-fourth of the global population, with the highest prevalence among children younger than 5 years (1). Global anemia is commonly attributed to iron deficiencies resulting from low iron intake from foods (2, 3), folate (4), and vitamin B12 deficiencies (5), worm infections, and malaria (6). Anemia linked to iron deficiency among women and children exerts the heaviest toll in terms of ill-health and premature deaths, resulting in the loss of productivity and earnings in Low and Middle-income countries (LMICs). Anemia during early childhood years also has debilitating effects on cognitive development, leading to lifelong compromised vitality and ability. Anemia is not a disease *per se*, but a manifestation of hidden deficiencies with several overt symptoms (2). As such, policymakers, service providers, and communities tend to overlook anemia's significant societal costs and health consequences. In 2015, however, acknowledging anemia as a public health priority, the United Nations General Assembly (UN-GA) explicitly outlined the complete elimination of anemia (along with other forms of malnutrition) by 2030 in its Sustainable Development Goals (SDG) (Target 2.2) (7).

Globally, the prevalence of anemia continues to be highest in India, with more than half of its maternal and child population affected (8). Recent findings from the National Family Health Survey (NFHS), 2019–2021 report prevalence of anemia among children (6–59 months) at 67.1% and among women (15–49 months) at 57% (9). High anemia burden remains a public health priority in India for two noteworthy reasons. First, despite consistent policy efforts like National Nutrition Control Anemia Program (NNCAP), Weekly Iron and Folic Acid Supplementation (WIFS), and National Iron Plus Initiative (NIPI), long-term improvements in maternal and childhood anemia remain elusively substandard—as an example, the observed decline in prevalence among children was only 1.1 percentage point per annum between 2005–06 and 2015–16 (10). Despite episodes of rapid economic growth during the above timeframe, gains in prosperity did not translate into better nutritional outcomes across the country. In 2018, the Government of India (GOI) launched a flagship program POSHAN *Abhiyaan* (Prime Ministers Overarching Scheme for Holistic Nutrition—previously the National Nutrition Mission), with an explicit focus on improving maternal and child nutritional outcomes, including a targeted three percentage points per annum reduction in anemia (11). Additionally, in 2018 the GOI partnered with UNICEF to launch *Anemia Mukt Bharat* (AMB) with the specific goal of reducing anemia among both children and women of reproductive age (12). Despite all of these high-profile initiatives, the persistently high burden of anemia in India suggests an increasingly urgent need to strengthen and sharpen the policy design for these interventions.

Second, unlike most malnutrition measures in India which have registered positive declines to various degrees over the last 5 years, the anemia burden has actually increased. Compared to the levels in the NFHS 2015–2016, anemia prevalence among children (6–59 months) is 8.5 percentage points higher in the NFHS 2019–2021 (9). This translates to a 1.7 percentage point increase per annum. In the same vein, the proportion of anemic women in the country has increased from 53.1% to 57.0% between 2015–16 and 2019–21 (9). While this may be partially attributable to COVID-19-induced disruptions, such regressive trends are alarming and warrant an expedient reassessment of policy design and targeting.

Identifying vulnerable regions and subpopulations most at risk within the larger monitored areas is critical for effective policy design. To this end, an emerging concern with India's current strategy is its exclusive focus on district-level estimates for prioritizing interventions (12). As an example, the implementation, monitoring, and evaluation of iron-folic supplementation under *Anemia Mukt Bharat* are carried out at the district level. Focusing solely on the district-level estimates overlooks any small area variation within districts in the reported outcomes, as anemia prevalence at different geographical levels (states, districts, villages) can differ significantly depending on the area. Recent research shows that in the case of anthropometric failures such as stunting, geographic variation attributable to states (13, 14), and villages (15–17) is much higher than variation between districts. (18). While previous studies have examined the risk factors for anemia among children in a geographic (spatial) multilevel model, none have parsed out the relative difference in variations across the geographies (19–21).

This study examines the small area variation within districts—the current targeting geographical unit—in the prevalence of three grades (hemoglobin cut-off-based) of anemia (severe, moderate, and mild) among women and children across 707 districts in India using recently released National Family Health Survey, 2019–21. The rationale behind segregating the analysis by three anemia grades stems from the difference in the nature and intensity of treatment interventions. While remedies for mild and moderate anemia are measures like food and iron supplementation, cases of severe anemia may additionally warrant urgent clinical treatment. More specifically, the objective of the study is to quantify and elucidate on the geographical variation in the prevalence of anemia within the macro policy units in India—i.e., states, districts, and villages/blocks. It is worth mentioning here that we estimated the variation within and across geographical policy units. This is because in the context of current policy making in India, the targeting strategy is based on district-level prevalence and therefore, this study aims to underscore the relevance of the overlooked geographical variation within these macro-policy units. As previous studies have not quantified India's small area variations in maternal and childhood anemia, the findings from this analysis can substantially contribute to ongoing policy efforts to eradicate anemia and achieve the target SDG 2.2 goal.

## Methods

### Overview

We utilized data from the recently released fifth round of the National Family Health Survey (equivalent to the Demographic Health Survey) conducted in 2019–21. The NFHS-5 released the data for 707 districts nested within all 36 states and UTs. Initiated in the early 1990s, the NFHS is part of the Demographic Health Surveys (DHS) program and provides individual-level information on important population, health, and nutrition indicators. Since its inception, DHS has conducted more than 400 surveys in 90 countries.

### Data

The NFHS adopts a multistage, stratified cluster sampling design. Microdata from the fifth round of the survey is the latest one available and was used in this study. The NFHS collects the data for rural and urban areas separately using the latest census data as the sampling frame. According to the Demographic Health Survey, the clusters, which are villages (for rural areas) and Census Enumeration Blocks (CEBs) (for urban areas), serve as primary sampling units (PSU). A representative sample of households was constructed for rural areas *via* stratified, probabilistic two-stage random sampling. In the first stage, the PSU (or cluster) corresponding to villages was classified on key variables and selected by probability proportional to cluster size. This was followed by household selections from the household list using systematic sampling with equal probability (10). A similar process was used for the urban areas, but because the urban clusters correspond to CEBs, a mix of a two-stage sampling approach was employed (10). It may be noted that PSUs with more than 300 households are divided into 100 to 150 household segments. Hence one cluster can be either a PSU or a segment of a PSU.

### Study population and sample size

The NFHS-5 provides data for 232,920 children (0–59 months) and 724,115 women (15–49 years) nested within 707 districts in all 36 states/Union Territories of India. After excluding the information on deceased children, age criteria (those <6 months), and missing (flagged) observations on demography and hemoglobin, the final analytic sample for the analysis was 183,883 children (6–59 months) and 690,153 women (15–49 years). Supplementary Figure 1 reports the construction of the analytic sample, original sample size, inclusion and exclusion criteria, and the final sample used for the analysis.

### Primary outcomes

The primary outcome variables were three binary grades (Yes = 1/No = 0) of anemia (mild, moderate, and severe) based on hemoglobin levels (g/dl) of women (15–49 years) and children (6–59 months). Hemoglobin among women and children was measured using a finger prick blood sample. Children with hemoglobin levels between 10.0 and 10.9 g/dl were coded as mildly anemic, between 7.0 to 9.9 g/dl as moderately anemic, and levels lower than 7.0 g/dl as severely anemic. Similarly, for women aged 15–49 years, mild and moderate anemia was defined as hemoglobin levels from 11 to 11.9 g/dl (10.0 to 10.9 g/dl for pregnant women) and from 8.0 to 10.9 g/dl (7 to 9.9 g/dl for pregnant women), respectively. For severe anemia, the cutoff was 8.0 g/dl (7.0 g/dl for pregnant women).

### Statistical analyses

We employed a four-level logistic regression model to partition the total geographical variation in the women and childhood anemia (Y) across child/anemia *i* (level-1); cluster *j* (level-2); district *k* (level-3); state *l* (level-4): *Y*_*ijkl*_ = β_0_+(*u*_0*jkl*_+ *v*_0*kl*_+ *f*_0*l*_). In the model mentioned above, *u*_0*jkl*_, *v*_0*kl*_, *f*_0*l*_ are model residuals specific to cluster, district, and state, respectively. These set of residuals are assumed to have a normal distribution around the mean of 0 and the variance of *u*_0*jkl*_ ~ (0, $\sigma_{u0}^{2}$); *v*_0*kl*_ ~ (0, $\sigma_{v0}^{2}$); *f*_0*l*_ ~ (0, $\sigma_{f0}^{2}$). Here, the term $\sigma_{u0}^{2}$ denotes within-district, inter-cluster variation, $\sigma_{v0}^{2}$ denotes within-state, inter-district variation and $\sigma_{f0}^{2}$ stands for inter-state variation. Variance across individual children and women is not computed directly for binary outcomes and is instead assumed to follow a logistic distribution with a fixed variance of π^2^/3 or 3.29 (22). We then computed the variance partitioning coefficient (VPC hereafter) to assess the significance of each geographical unit (z) in total variability as ($\frac{\sigma_{z}^{2}}{\sigma_{u0}^{2}+\;\sigma_{v0}^{2}+\;\sigma_{f0}^{2}}$) ^*^ 100. It may be noted that we did not include individual-level fixed variance in the computation of VPC. This is because incorporating a fixed constant will not change the relative share of each geography and therefore, the qualitative interpretation of the findings. We also performed the multilevel regression after adjusting the model for the place of residence i.e., rural and urban areas. Multilevel modeling was performed using the STATA 15 (23) and MLwiN 3.0 software program (24) (using *runmlwin*) (25) and the Monte Carlo Markov Chain (MCMC) method using the Gibbs sampler, keeping the default prior distribution of Iterated Generalized Least Square (IGLS) as the starting value (22).

Based on the multilevel logistic model estimates, we then generated precision-weighted cluster level predicted probabilities of anemia for both women and children. For more robust estimates, these probabilities were predicted by pooling information (and borrowing strength) from other clusters that share the same district membership (26). The probability of each Y for each village/block was calculated as exp((β_0_+*u*_0*jkl*_+ *v*_0*kl*_+ *f*_0*l*_) + (1/*exp*(β_0_+*u*_0*jkl*_+ *v*_0*kl*_+ *f*_0*l*_)). From the above precision-weighted estimates, we then computed within-district, inter-cluster variation (small area variation) in anemia *via* standard deviation (SD). All maps were generated using ArcGIS Pro 2.9.1 (27). The shapefiles for the 707 districts as per the NFHS-5 survey were obtained from the International Institute for Population Sciences (IIPS), the administrating organization of the NFHS-5 survey in India.

## Results

### Sample characteristics—anemia among women and children

**Figure 1:**
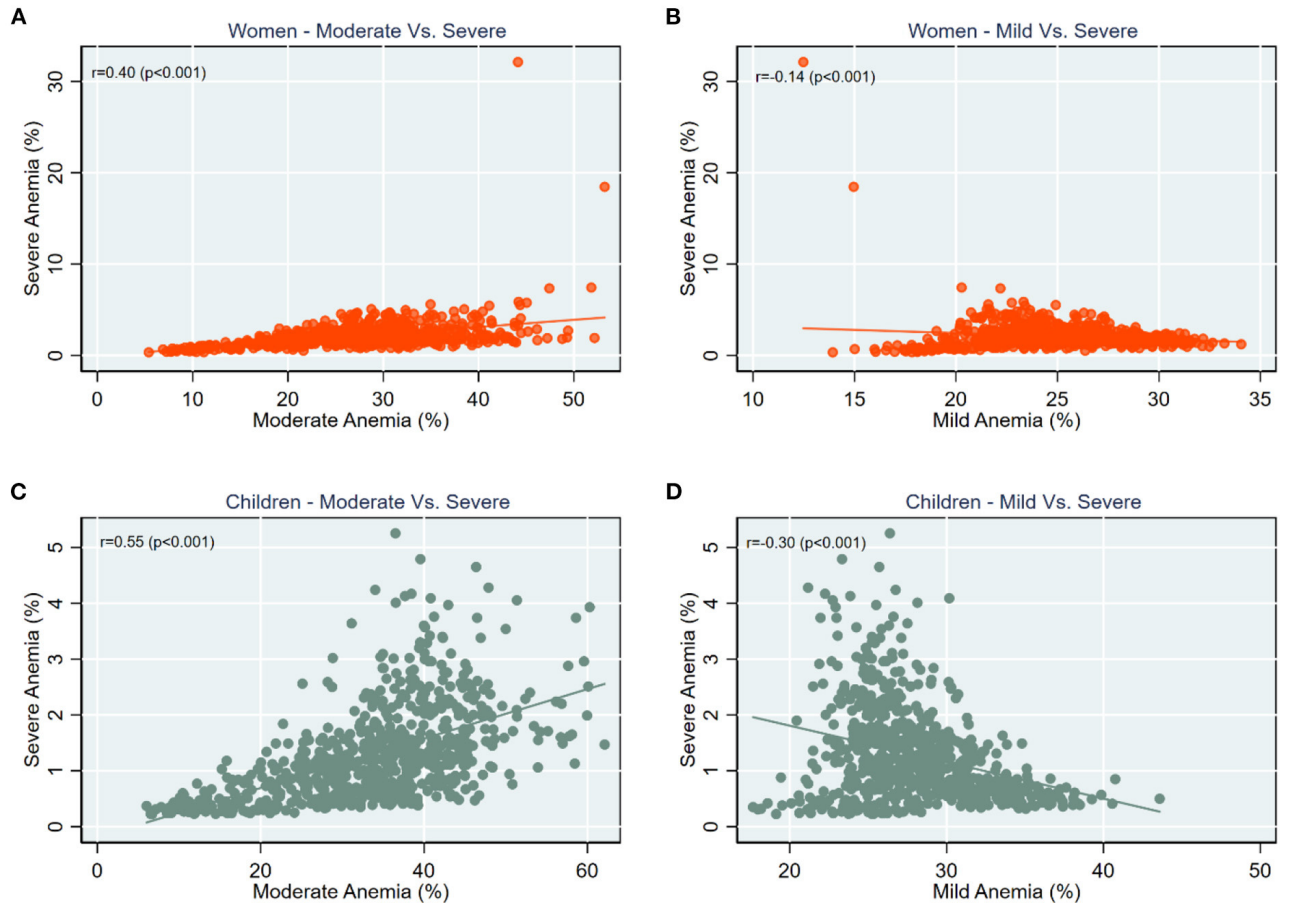
Scatter plots for correlation between prevalence of **(A)** Women—moderate vs. severe anemia; **(B)** Women—mild vs. severe anemia; **(C)** Children—moderate vs. severe anemia; **(D)** Children—mild vs. severe anemia, India, NFHS 2021.

Of the total sample children, 28.7% (*n* = 52,480) were mildly anemic, 35.4% (*n* = 64,941) were moderately anemic, and 2.0% (*n* = 3,609) were severely anemic (Supplementary Table 1). Among sample women, the prevalence of mild anemia was 25.2% (*n* = 173,893), and moderate anemia was 28.3% (*n* = 195,685). 2.6% (*n* = 18,221) of the total sample women were severely anemic (Supplementary Table 1). Figure 1 reveals a null to moderate correlation between different grades of anemia across districts in India. For example, while a low correlation (*r* = 0.40; *p* < 0.001) was observed between moderate and severe anemia (Figure 1A), it was much lower (*r* = 0.14; *p* < 0.001) between mild and severe anemia among women (Figure 1B). Further, correlation between mild and severe anemia among children was also weak (*r* = 0.30; *p* < 0.001) (Figure 1D). The correlation pattern was similar for SD in different grades of anemia across districts (Supplementary Figure 2).

### The relative importance of geographic levels

**Figure 2:**
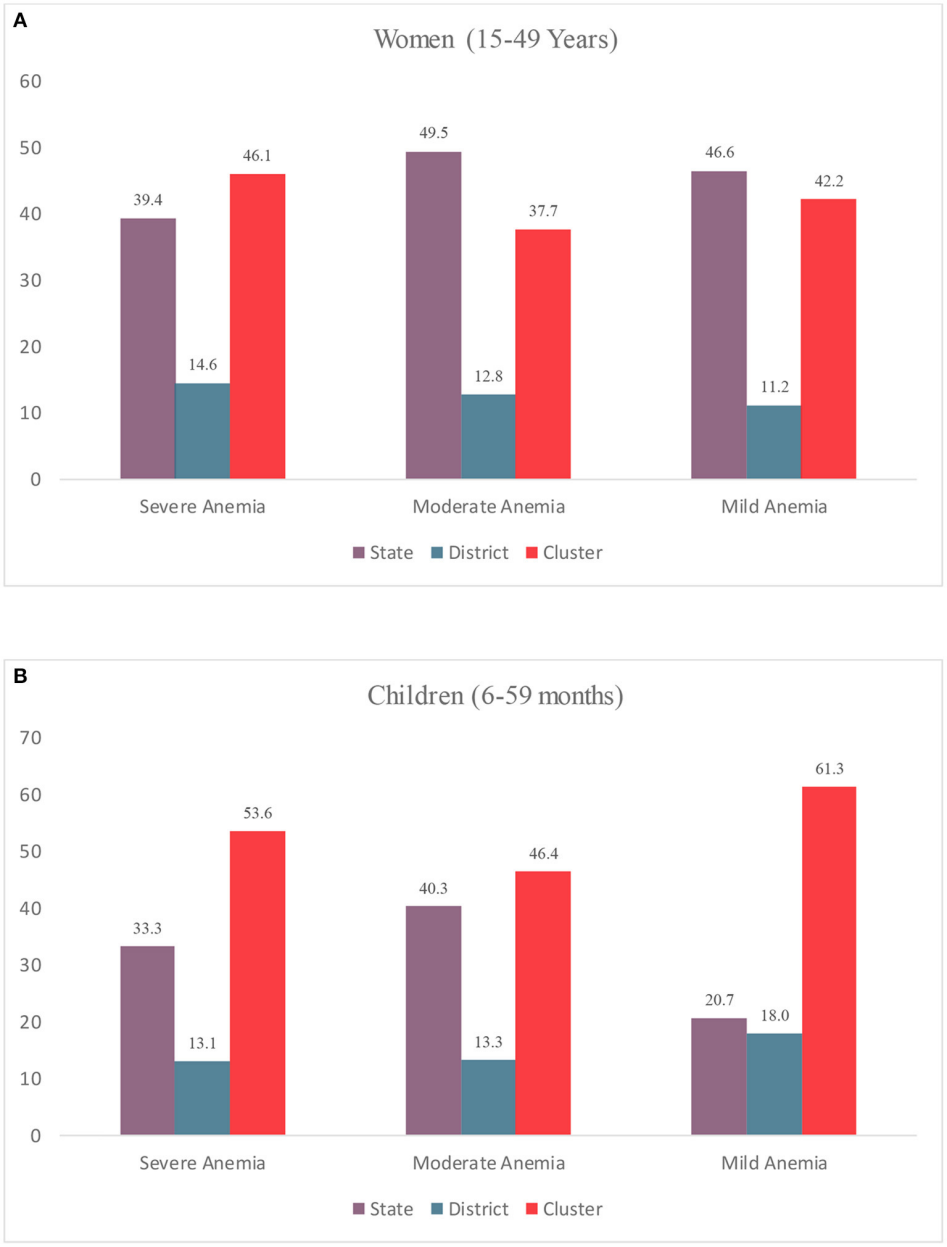
Variance Partitioning Coefficient (VPC) (%) in anemia among **(A)** Women (15–49 years); **(B)** Children (6–59 Months) by multiple geographies, India, NFHS 2021.

The variance partitioning estimates from the multilevel logistic regression indicate that smaller units (clusters) account for the largest proportion of the total variation in severe anemia among women and children (Figure 2). In the case of severe anemia among women, about 46.1% was attributed to the clusters (Var: 0.494; SE: 0.150), followed by 39.4% (Var: 0.156; SE: 0.134) to states, and 14.6% to districts (Var: 0.156; SE: 0.012) (Figure 2A).

In the case of moderate anemia, states accounted for the highest variation (49.5%; Var: 0.290; SE: 0.078), followed by cluster (37.7%; Var: 0.221; SE:0.003), and districts (12.8%; Var: 0.0.075; SE: 0.004). Among children, the share of clusters in the total variation was highest for mild (53.6%; Var: 0.899; SE: 0.069) w, moderate (46.4 %; Var: 0.0.398; SE: 0.011) and mild (61.3%; Var: 0.092; SE: 0.016) anemia.

**Figure 3:**
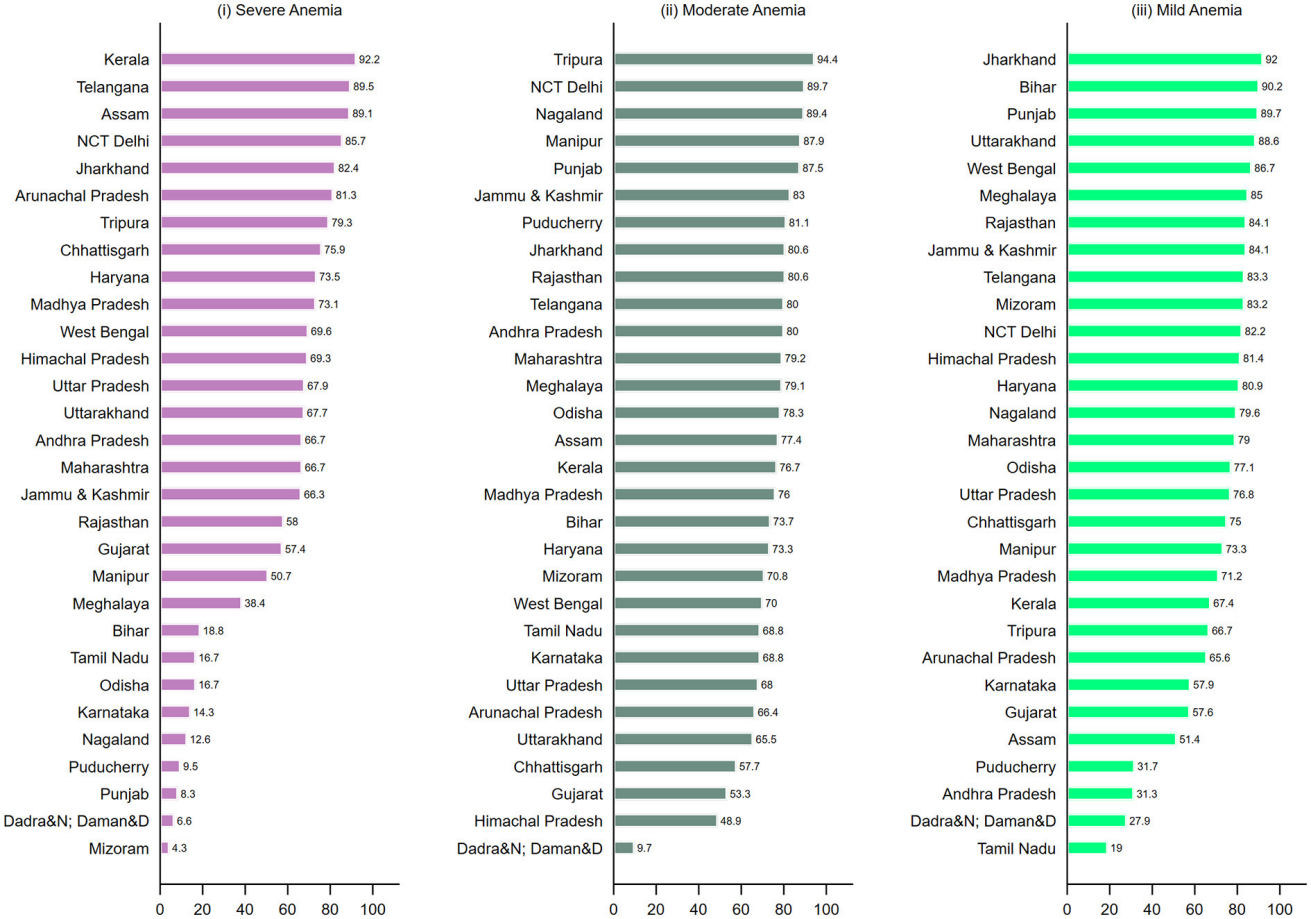
Geographic variation of (i) Severe anemia; (ii) Moderate anemia; (iii) Mild anemia among women (15–49 years) attributable to small areas (cluster) (%) across states and Union Territories in India, NFHS, 2021.

The findings from these three-level models reveal that clusters account for the majority of total variation in anemia among women in most Indian states (Figure 3). Some top states with more than 80 % of variability across clusters include Kerala (92.2%), Telangana (89.5%), Assam (89.1%), NCT Delhi (85.7%), and Jharkhand (82.4%) (Panel i). In about 20 states, more than 50% of the total variation in severe anemia was attributable to small areas. For moderate anemia (Panel ii), clusters again account for a major share (more than 50%) in total variation for 80% of states/UTs (28 out of 36). A similar pattern was observed for mild anemia among women (Panel iii). Share of clusters in total variation was more than 60% in about 23 states, with clusters in certain states (Jharkhand, Bihar, Punjab) accounting for more than 90% of variation.

**Figure 4:**
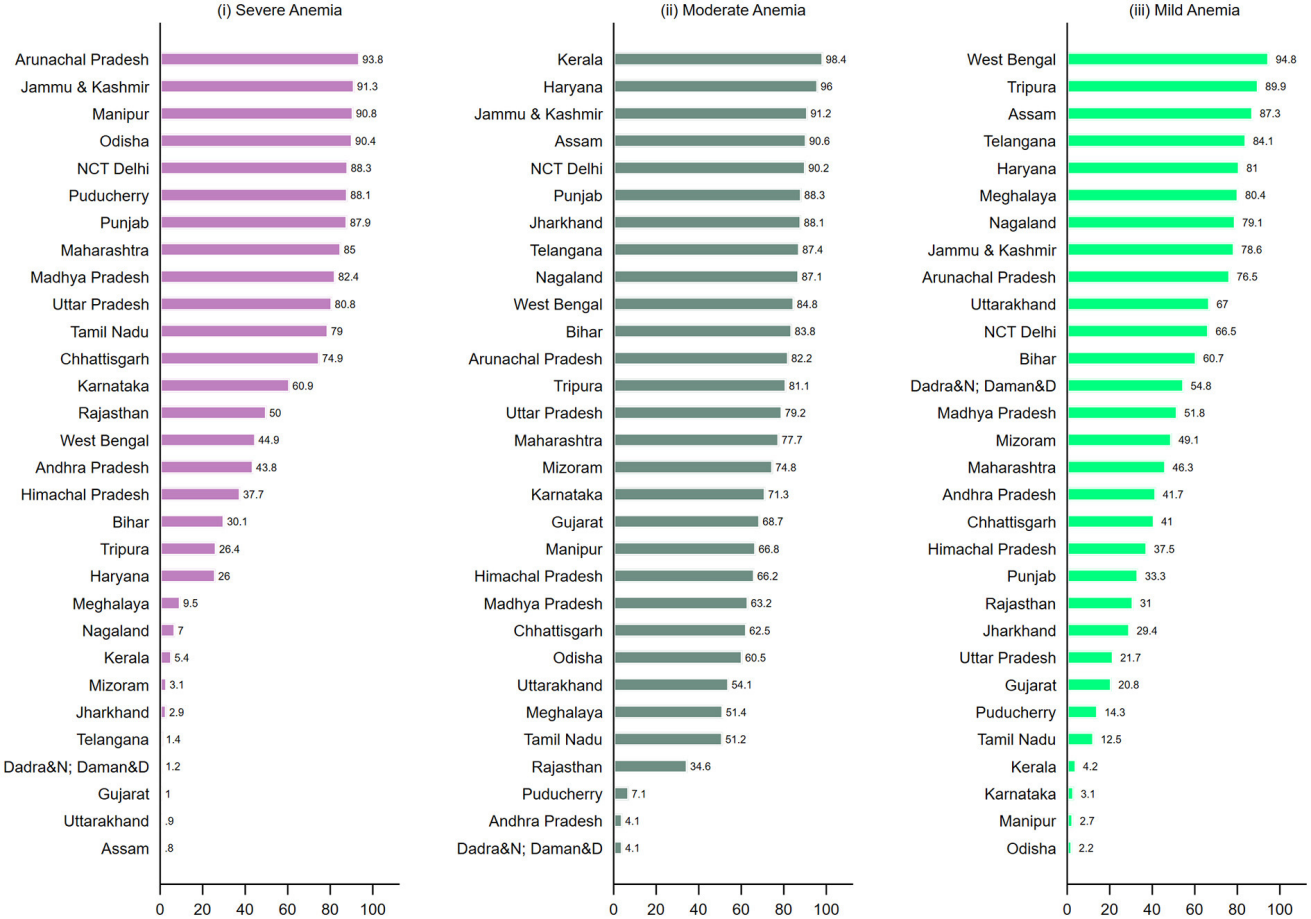
Geographic variation of (i) Severe anemia; (ii) Moderate anemia; (iii) Mild anemia among children (6–59 months) attributable to small areas (clusters) (%) across states and Union Territories in India, NFHS, 2021.

For severe anemia among children, variation attributable to clusters was more than 50% in 14 states (UTs) (Figure 4-i), and more than 90% in Arunachal Pradesh (93.8); Jammu & Kashmir (91.3); and Manipur (90.8). For variation in moderate anemia (Panel ii), 26 states present more than a 50% share of clusters, including four states with more than 90%, Kerala (96.4%), Haryana (96.0%), Assam (90.1%), and J&K (90.6%). A similar pattern was observed across states for mild childhood anemia (Panel iii) with higher variability between small area in most of the states. The findings were consistent after adjusting the regression models for household's place of residence (Supplementary Table 3). For moderate (OR: 1.14; 95% CI 1.12; 1.17) and severe anemia (OR: 1.23; 95% CI: 1.17; 1.29) among women, the likelihood was higher for women from rural areas than urban areas (Supplementary Table 4).

### Mean prevalence and small area variation (SD)

We computed inter-cluster standard deviations (SD) of the prevalence of each outcome by district. These values quantified anemia's small area variation within districts. The SD across districts ranges from 0.73 to 2.87 for mild anemia, 1.15 to 16.0 for moderate anemia, and 0.01 to 28.8 for severe anemia among women (Supplementary Figure 3). Similarly in the case of children, the SD across districts ranges from 0.91 to 3.26 (IQR: 0.62) for mild anemia, 1.12 to 13.3 (IQR: 2.64) for moderate anemia, and 0.01 to 07.95 (IQR: 0.67) for severe anemia. Notably, the range of SDs across districts was higher in the case of mild or moderate anemia among women compared to that among children.

**Figure 5:**
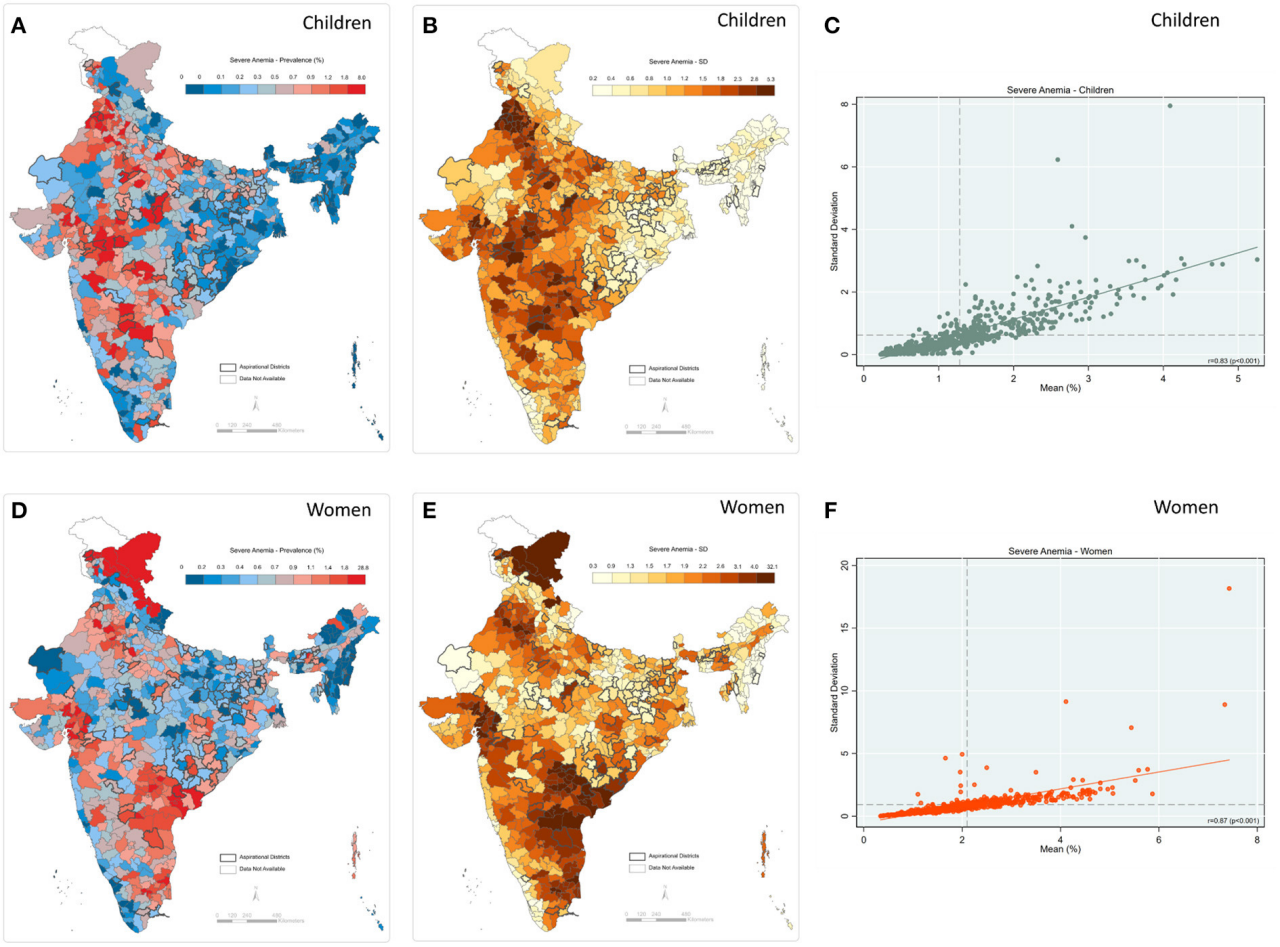
Severe anemia **(A)** Map-district-level prevalence for children; **(B)** Map-inter-cluster SD for children; **(C)** Scatter-prevalence vs. SD for children; **(D)** Map-district-level prevalence for women; **(E)** Map-inter-cluster SD for women; **(F)** Scatter-prevalence vs. SD for women, India, NFHS 2021.

Mapping of severe anemia among children reveals a concordance in the mean prevalence and inter-cluster SD across districts (Figures 5A,B). Districts with a high mean (red shade) and high SD (brown shade) are clustered around India's central and south-eastern regions. Further, a significantly high and positive correlation was observed between prevalence and inter-cluster SD (*r* = 0.83; *p* < 0.001) (Figure 5C). The geographic pattern for prevalence and inter-cluster SD is similar for severe anemia among women (Figures 5D,E). For example, high mean and high SD were observed in the south-eastern regions along with scatter plots, reflecting a high and positive correlation (*r* = 0.81; *p* < 0.001) (Figure 5F). The state-level estimations also suggest a moderate to a high correlation between prevalence and inter-cluster SDs for severe anemia among both women and children (Supplementary Tables 5, 6). Further, 550 out of the 707 districts were concentrated in the same tertiles (i.e., high prevalence-high SD; low prevalence-low SD) of severe anemia among women and children (Supplementary Table 7).

**Figure 6:**
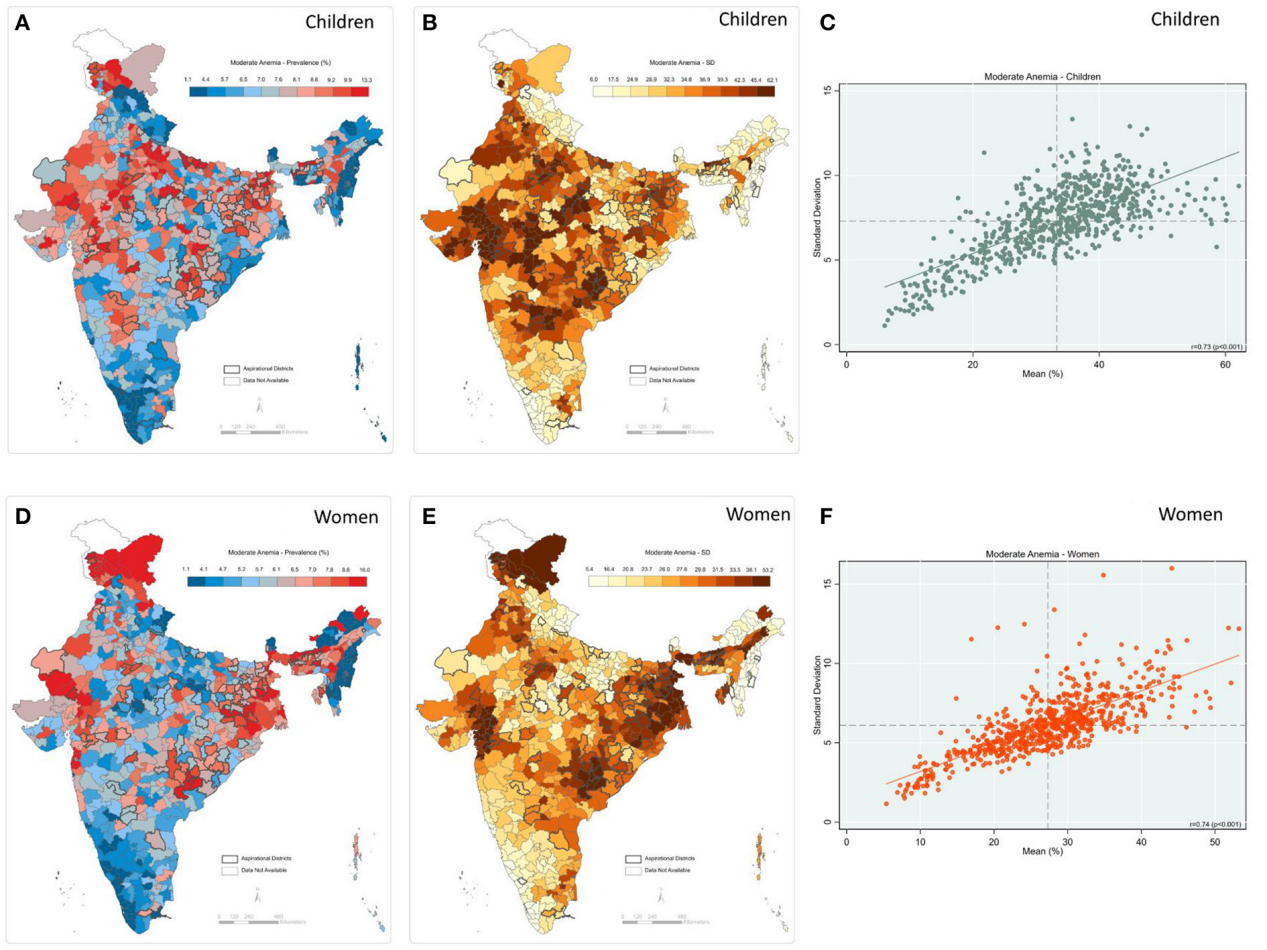
Moderate anemia **(A)** Map-district-level prevalence among children; **(B)** Map-inter-cluster SD for children; **(C)** Scatter-prevalence vs. SD for children; **(D)** Map-district-level prevalence for women; **(E)** Map-inter-cluster SD for women; **(F)** Scatter-prevalence vs. SD for women, India, NFHS 2021.

A moderately high and positive correlation between prevalence and inter-cluster SD was observed for moderate anemia among both children (*r* = 0.73; *p* < 0.001) and women (*r* = 0.74; *p* < 0.001) (Figure 6). In addition, the geographic pattern for prevalence was akin to that of inter-cluster SD—for example, central and western parts of India display both high prevalence and inter-cluster SD among children (Panel A & B). Further, the eastern region shows a high mean prevalence and high SD for moderate anemia among women (Panel 5D and 5E). State-level estimates also reflect a moderate to a high correlation between prevalence and inter-cluster SD (Supplementary Tables 5, 6).

**Figure 7:**
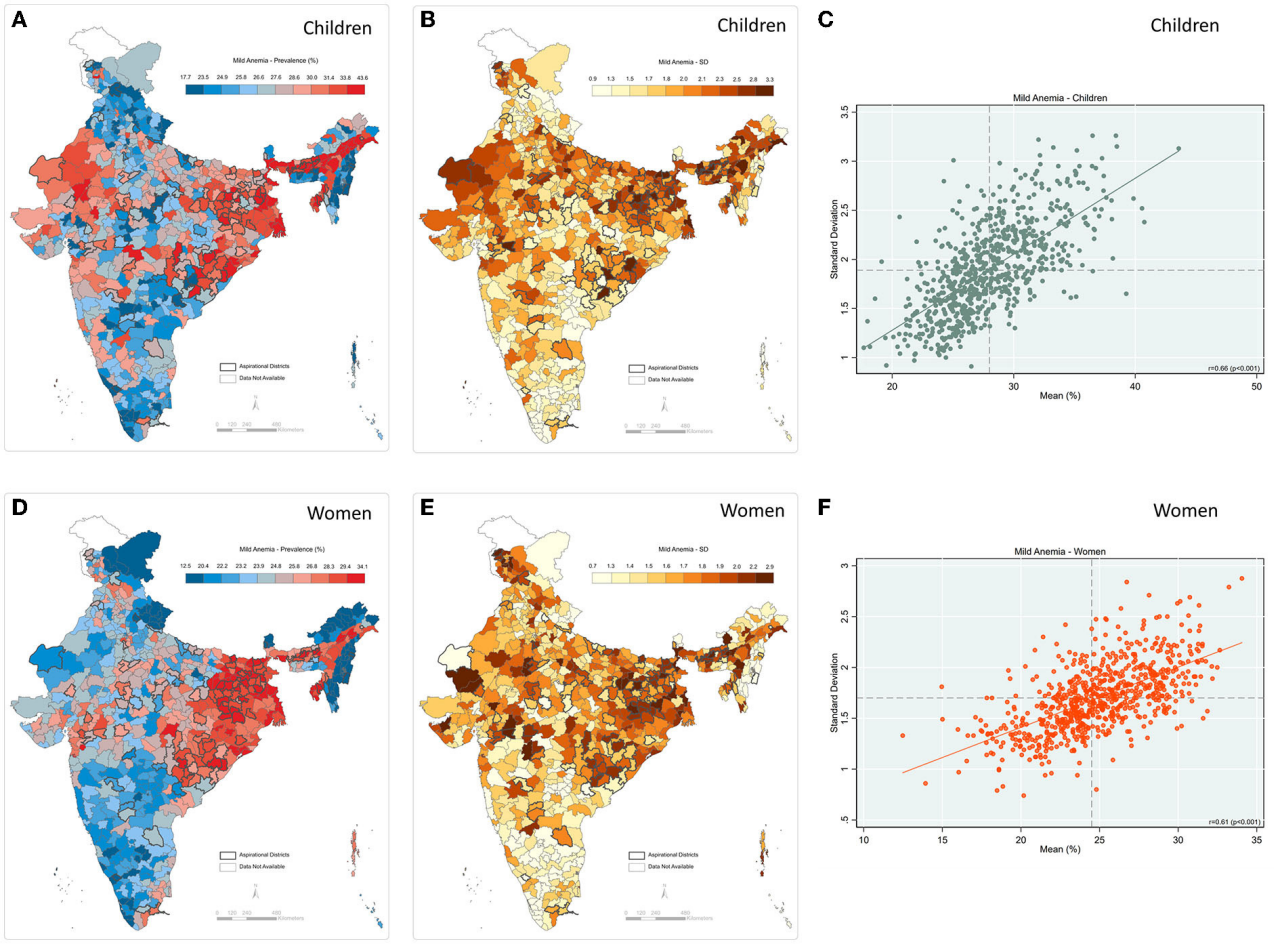
Mild anemia **(A)** Map-district-level prevalence for children; **(B)** Map-inter-cluster SD for children; **(C)** Scatter-prevalence vs. SD for children; **(D)** Map-district-level prevalence for women; **(E)** Map-inter-cluster SD for women; **(F)** Scatter-prevalence vs. SD for women, India, NFHS 2021.

We observed a comparatively lesser similarity in the geographic pattern of district-level prevalence and inter-cluster SD for mild anemia among children and women (Figure 7). The mapping reveals that the pockets of the high burden of mild anemia among children and high SD are not evenly spread across the country, the scatter plot confirming a moderately positive correlation (*r* = 0.66; *p* < 0.001) between prevalence and SD (Figure 7C). A null to moderate correlation is observed across the states (Supplementary Table 3). For mild anemia among women, this pattern is even less robust, with a correlation coefficient (r) of 0.61 (*p* < 0.001) (Figure 7F)—and negative for Jharkhand (*r* = −0.32) and Gujarat (*r* = −0.10) (Supplementary Table 4). Similarly, correlation was very low for the states of West Bengal (*r* = 0.06), Punjab (*r* = 0.17), and Bihar (*r* = 0.11) (Supplementary Table 3).

Categorization of districts across prevalence and SD tertiles also suggests discordance, as about 304 districts were in different tertiles (Supplementary Table 7). Supplementary Tables 8, 9 present prevalence estimates and inter-cluster SD for all three grades of anemia among women and children across 707 districts.

## Discussion

This study quantifies and examines the geographic variation in anemia among women and children across India's states, districts, and villages. Our analysis reveals four salient findings. First, a null to moderate positive correlation between mild/moderate and severe anemia across districts was observed, implying that districts with a low prevalence of mild or moderate anemia may nonetheless have a high burden of severe anemia (or vice versa). Second, within-district variation accounts for the highest share of total geographic variance in severe anemia among women and children across all three geographies, a pattern that was consistent for most of the states and UTs—among children, within-district variation was highest for all three grades of anemia. This finding is consistent with previous multilevel studies on partitioning variance of India's socioeconomic, health, and undernutrition indicators (13, 28, 29). Third, we found a strong positive correlation between prevalence and inter-cluster SD of severe anemia among women and children, respectively, suggesting that districts with a higher overall burden of severe anemia also tend to have larger small area variations. For mild anemia, however, the strength of the correlation was less strong, indicating the possibility that low burden districts can still have higher variation. Finally, an examination of spatial distribution reveals a notable concentration of high burden and high SD of severe anemia among women from southeastern parts of the country like Telangana and Andhra Pradesh, whereas severe anemia among children is concentrated in central west region of the country.

The observed discordance across districts between the prevalence of mild/moderate anemia and that of severe anemia is of potential importance for fine-tuning the strategic focus of anemia interventions. Despite POSHAN *Abhiyaan's* goal to reduce anemia prevalence among children and women by three percentage points per year, the program does not currently distinguish between different grades of anemia for its targeted interventions. Given our observations of variation across grades, it is imperative to factor in these differences when developing an effective strategy. For example, in the Kargil district of Ladakh, the prevalence of mild anemia was 12.48% (better compared to the national average), but the prevalence of severe anemia was very high at 32.1%. Designing interventions based on the prevalence of a composite metric (i.e., any anemia) without considering the variation across grades of severity risks limiting the success of the intended outcomes. This becomes even more important when considering the magnitude of difference across grades—while the prevalence of mild and moderate anemia among children is 28 and 29%, respectively, only 1.6% of the total children were severely anemic.

Our findings take on policy salience in the crucial work of anemia-focused programs accurately selecting the appropriate geographic unit for program targeting, monitoring, and evaluation. In the case of severe anemia among both women and children, most of the geographic variation is sourced at the cluster level (village/blocks), which underscores the importance of these smaller geographic units. Among children, this finding was consistent across all grades of severity. Previous studies on poverty, child malnutrition, and associated risk factors have similarly asserted the importance of smaller area units in total geographic variation (12, 13, 28, 29). Further, in most states—those comprising more than 70% of India's population—we found that clusters account for the highest share in the total geographic variation. Despite such relevance, most of India's maternal and child health and nutrition programs like POSHAN *Abhiyaan* currently rely on district-level averages for targeting, governance, and research purposes (30). Even more narrowly focused programs like *Anemia Mukt Bharat* use district-level data to monitor progress and prioritize interventions. Given India's widespread deprivations, geographical and sociocultural heterogeneities, and limited resource settings, these programs must prioritize selecting the most effective geographical policy unit [state, district, village/block] when designing interventions, an especially critical need for severely anemic cases requiring urgent and more intense clinical interventions. It is important to mention here that a few studies have also included the salience of inter-household and inter-individual variation in the health outcomes (31–34). It will be interesting to further extend the scope and address the question of whether the policy targeting strategy to reduce anemia should be “universal” (for all individuals) or “geographical area-specific”.

In the case of severe anemia among both women and children, the strong and positive correlation between prevalence and SD is critical to note, indicating that districts with a higher overall burden are also subject to higher variation between communities. For instance, severe anemia among women in Kishtwar district of Jammu and Kashmir was 7.4% with a high inter-cluster SD of 18.6%. Although the current strategy in India is to design the targeting intervention based on district-level averages of nutritional outcomes, we assert that considering small area variation can further enhance policy efficiency by not overlooking the high burden clusters within the district. Importantly, even for mild anemia, we found a moderate positive correlation between district prevalence and inter-cluster SD among women and children. This warrants special attention because districts with a low prevalence of mild anemia might mask the high inter-cluster variation. This finding has additional significance, given that the prevalence of mild anemia is highest across the three grades of severity. Moreover, the most common drivers of anemia, such as household income and dietary deprivations, vary mostly between villages within a district (35), further emphasizing the importance of looking at small area variation when formulating the most appropriate intervention for a targeted outcome. These findings can also be calibrated with key performance indicators (interventions) under anemia-specific programs like Anemia *Mukt Bharat* (36, 37). For example, in Punjab, the 87.5 percent of geographic variation in anemia is attributed to clusters, and only 11.8 percent of children were given 8–10 doses of IFA syrup (a key program performance indicator) (36).

This study is not without limitations. Precision weighted estimates for mean prevalence and inter-cluster SD are not adjusted for the demographic and socioeconomic inequalities that may drive the burden of anemia. Additionally, while our analysis shows the variation across small areas (villages/blocks) within a district, rural villages and urban blocks are not directly comparable in demographics, size, administrative role, and relevance.

This analysis suggests that small area (villages/blocks) inequality in anemia outcomes across Indian districts calls for more precise policy targeting. Substantial state-level heterogeneities in anemia prevalence can be attributed to these small areas, strengthening the argument that current (and future) initiatives such as POSHAN *Abhiyaan* and *Anemia Mukt Bharat* should prioritize within-district variation when designing a targeting strategy such as Iron Folic Acid (IFA), deworming tablets, and food and nutrition supplementation. The findings from this study can be utilized by anemia-focused programs to determine the appropriate geographic/policy unit for efficiently channeling efforts and resources to help effectively achieve India's Sustainable Development Goals within the stated 2030 time frame.

## Data availability statement

Publicly available datasets were analyzed in this study. This data can be found here: www.dhsprogram.com.

## Ethics statement

This project used publicly accessible secondary data obtained from the DHS website. The DHS data are not collected specifically for this study and no one on the study team has access to identifiers linked to the data. These activities do not meet the regulatory definition of human subject research. As such, an Institutional Review Board (IRB) review is not required. The Harvard Longwood Campus IRB allows researchers to self-determine when their research does not meet the requirements for IRB oversight *via* guidance online regarding when an IRB application is required using an IRB Decision Tool.

## Author contributions

SR did data management, analysis, and reporting and led the writing of the manuscript. AK, MJR, RK, and SVS contributed to the manuscript's writing and provided a critical review of the data analysis and reporting of results. RK and SVS provided overall supervision. All authors contributed to the article and approved the submitted version.

## Funding

This work was funded by the Bill and Melinda Gates Foundation, INV-002992. RK would like to acknowledge support from Korea University, Grant K2122891.

## Conflict of interest

The authors declare that the research was conducted in the absence of any commercial or financial relationships that could be construed as a potential conflict of interest.

## Publisher's note

All claims expressed in this article are solely those of the authors and do not necessarily represent those of their affiliated organizations, or those of the publisher, the editors and the reviewers. Any product that may be evaluated in this article, or claim that may be made by its manufacturer, is not guaranteed or endorsed by the publisher.
