# Supplementary material for: Small area variation in severe, moderate, and mild anemia among women and children: A multilevel analysis of 707 districts in India
Source: Front Public Health. 2022 Sep 20;10:945970. doi: 10.3389/fpubh.2022.945970 (PMC9530333; doi:10.3389/fpubh.2022.945970)
Supplement: Supplementary file 1 [file Data_Sheet_1.docx]

**SUPPLEMENTARY APPENDIX**

SUPPLEMENT TO:

Rajpal S, Kumar A, Rana MJ, Kim R and Subramanian SV (2022) "Small area variation in severe, moderate, and mild anemia among women and children: A multilevel analysis of 707 districts in India". Front. Public Health 10:945970. doi: 10.3389/fpubh.2022.945970

TABLE OF CONTENTS

| No. | Tittle | Page No. |
| --- | --- | --- |
| Figure S1 | Flow diagram showing exclusions and final sample sizes for primary analysis of the study population, Indian National Family Health Survey 2015-2021 | 2 |
| Table S1 | Descriptive Statistics of Response Outcomes, NFHS 2021, India |  |
| Figure S2 | Scatter plots for correlation between (A) Children – SD Mild Vs SD Severe Anemia; (B) Children –SD Moderate Vs SD Severe Anemia Prevalence; (C) Women – SD Mild Vs SD Severe Anemia Prevalence; (D) Women – SD Moderate Vs SD Severe Anemia Prevalence India, NFHS 2021. | 3 |
| Table S2 | Variation in Anemia among children (6-59 months) and Women (15-49 years) by Multiple Geographies, India, NFHS 2021 | 4 |
| Table S3 | Variation (VPC %) in Anemia among children (6-59 months) and Women (15-49 years) by Multiple Geographies adjusting for Place of Residence (Rural/Urban), India, NFHS 2021 | 5 |
| Table S4 | Odds Ratio for association between anemia (among children and women) and place of residence, India, NFHS, 2021 | 6 |
| Figure S3 | Dot plots showing distribution of standard deviations (SDs) of (A) Mild Anemia (B) Moderate Anemia; (C) Severe Anemia among Women and Children within districts. Note: Red lines represent median SD and blue lines represent 25th and 75th percentiles of SD. | 7 |
| Table S5 | Correlation (r) between district-level prevalence and within-district,  between small areas standard deviation of anemia among children by states, NFHS 2021, India | 8 |
| Table S6 | Correlation (r) between district-level prevalence and within-district,  between small areas standard deviation of anemia among children by states, NFHS 2021, India | 9 |
| Table S7 | Distribution of districts based on the district level percent and within district, between small areas standard deviation of Anemia among Children and Women in India, NFHS 2021 | 10 |
| Table S8 | Mean Prevalence and Standard Deviation of Anemia (Severe, Moderate, Mild) among children (6-59 months) Failures by Districts, India, NFHS, 2021 | 11-25 |
| Table S9 | Mean Prevalence and Standard Deviation of Anemia (Severe, Moderate, Mild) among Women (15-49 Years) by Districts, India, NFHS, 2021 | 26-40 |

Figure S1: Flow diagram showing exclusions and final sample sizes for primary analysis of the study population, Indian National Family Health Survey 2021

NFHS Original Sample Size

232920 children (0-59 months) and 724115 women (15-49 years)

8702 dead children excluded.

224218 alive children and 699686 alive women

Exclusion Criteria – Children aged below 6 months and missing age values.

| Category | Sample Children |
| --- | --- |
| Below 6 Months | 22788 |
| Missing Age Observations | 2955 |

198475 eligible sample of children aged 6-59 months 724115 women 15-49 years

Analytic sample of 183883 children aged 6-59 months and 690153 women aged 15-49 years for primary analysis

Exclusion on missing and flagged cases of Hemoglobin

| Category | Children | Women |
| --- | --- | --- |
| Missing | 7463 | 0 |
| Flagged | 7129 | 33962 |

Table S1**:** Descriptive Statistics of Response Outcomes, NFHS 2021, India

| Outcome | N | Prevalence |
| --- | --- | --- |
| Children (6-59 Months) |  |  |
| Mild Anemia (HB 10-10.9 gm/dcl) - No | 131043 | 71.3 |
| Mild Anemia (HB 10-10.9 gm/dcl) - Yes | 52840 | 28.7 |
| All | 183883 | 100 |
| Moderate Anemia (HB 7.0-9.9 gm/dcl) - No | 118942 | 68.6 |
| Moderate Anemia (HB 7.0-9.9 gm/dcl) - Yes | 64941 | 35.4 |
| All | 183883 | 100 |
| Severe Anemia (HB < 7.0 gm/dcl) – No | 180274 | 98.0 |
| Severe Anemia (HB < 7.0 gm/dcl) - Yes | 3609 | 2.0 |
| All | 183883 | 100 |
| Women (15-49 Years) |  |  |
| Mild Anemia* (HB 10-10.9 gm/dcl) - Yes | 516260 | 74.8 |
| Mild Anemia* (HB 10-10.9 gm/dcl) - No | 173893 | 25.2 |
| All | 690153 | 100 |
| Moderate Anemia (HB 7.0-9.9 gm/dcl) - Yes | 494468 | 71.7 |
| Moderate Anemia (HB 7.0-9.9 gm/dcl) - Yes | 195685 | 28.3 |
| All | 690153 | 100 |
| Severe Anemia (HB < 7.0 gm/dcl) - Yes | 671932 | 97.4 |
| Severe Anemia (HB < 7.0 gm/dcl) - No | 18221 | 2.6 |
| All | 690153 | 100 |

Figure S2**:** Scatter plots for correlation between (A) Children – SD Mild Vs SD Severe Anemia; (B) Children –SD Moderate Vs SD Severe Anemia Prevalence; (C) Women – SD Mild Vs SD Severe Anemia Prevalence; (D) Women – SD Moderate Vs SD Severe Anemia Prevalence India, NFHS 2021.


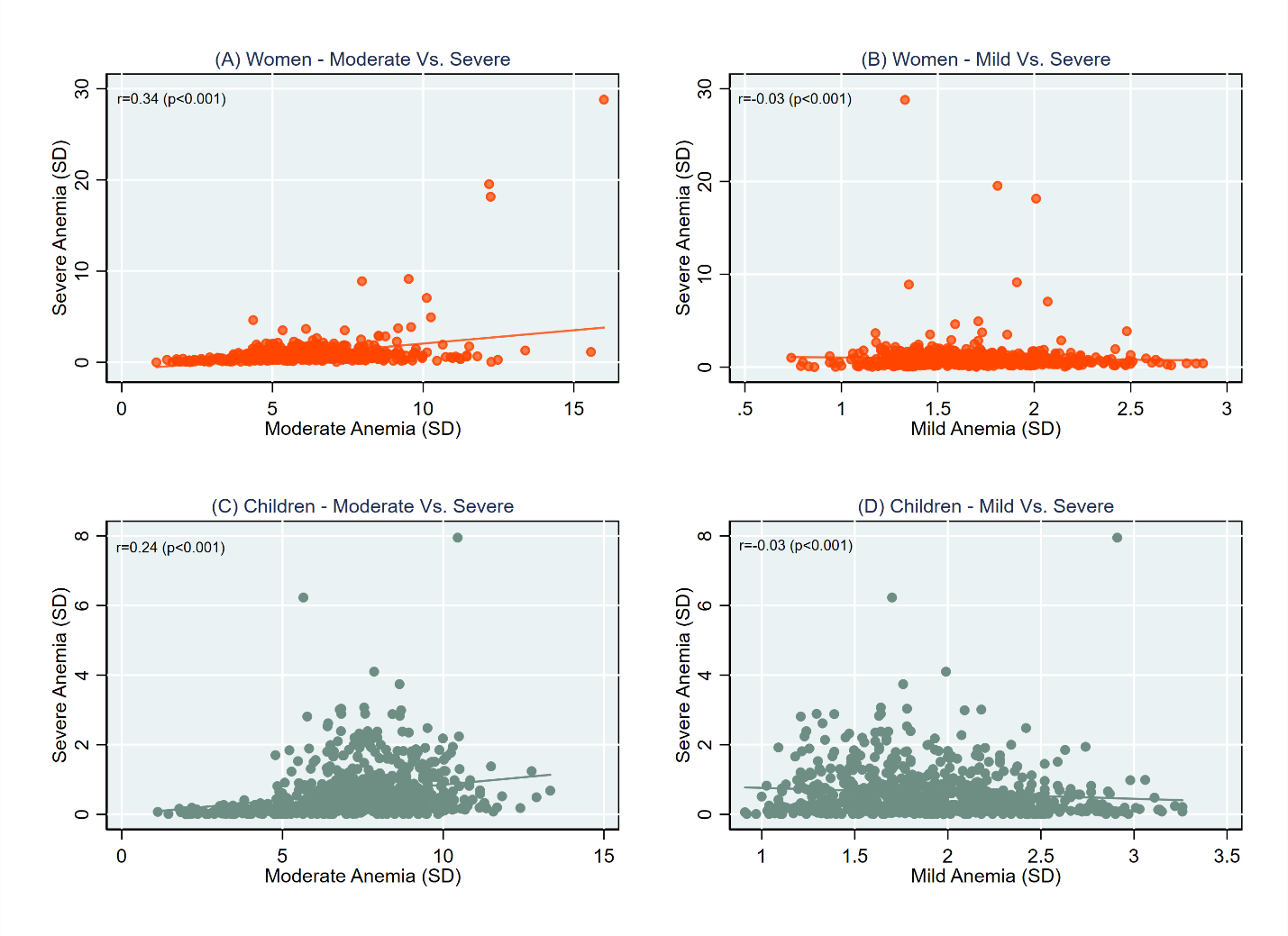


Note: SD = Standard Deviation

Table S2: Variation in Anemia among children (6-59 months) and Women (15-49 years) by Multiple Geographies, India, NFHS 2021

| **Overall Mean** | **Mild Anemia** | **Moderate Anemia** | **Severe Anemia** |
| --- | --- | --- | --- |
| **Variance partitioning - Women** |  |  |  |
| State | 0.054 *(0.014)* | 0.290 *(0.078)* | 0.422 *(0.134)* |
| District | 0.013 *(0.001)* | 0.075 *(0.004)* | 0.156 *(0.012)* |
| Village | 0.049 *(0.002)* | 0.221 *(0.003)* | 0.494 *(0.15)* |
| **Variance partitioning - Children** |  |  |  |
| State | 0.031 *(0.009)* | 0.346 *(0.095)* | 0.559 *(0.167)* |
| District | 0.027 *(0.002)* | 0.114 *(0.001)* | 0.220 *(0.028)* |
| Village | 0.092 *(0.016)* | 0.398 *(0.011)* | 0.899 *(0.069)* |

Table S3: Variation (VPC %) in Anemia among children (6-59 months) and Women (15-49 years) by Multiple Geographies adjusting for Place of Residence (Rural/Urban), India, NFHS 2021

| VPC - Child | Mild Anemia | Moderate Anemia | Severe Anemia |
| --- | --- | --- | --- |
| State | 0.032 (21.08%) | 0.347 (40.52%) | 0.571 (33.50%) |
| District | 0.027 (18.11%) | 0.114 (13.25%) | 0.228 (13.37%) |
| Village | 0.091 (60.81%) | 0.396 (46.23%) | 0.905 (53.13%) |
| VPC - Child | Mild Anemia | Moderate Anemia | Severe Anemia |
| State | 0.053 (46.36%) | 0.273 (48.32%) | 0.398 (38.17%) |
| District | 0.013 (10.98%) | 0.075 (13.21%) | 0.156 (14.95%) |
| Village | 0.049 (42.66%) | 0.217 (38.47%) | 0.489 (46.88%) |

Table S4: Odds Ratio for association between anemia (among children and women) and place of residence, India, NFHS, 2021

|  | Children | | Women | |
| --- | --- | --- | --- | --- |
| Mild Anemia | Odds Ratio | 95% CI | Odds Ratio | 95% CI |
| Urban^®^ | 1.00 |  | 1.00 |  |
| Rural | 0.97 | [0.94; 1.00] | 1.01 | [0.99; 1.03] |
| Moderate Anemia |  |  |  |  |
| Urban^®^ | 1.00 |  | 1.00 |  |
| Rural | 1.19 | [1.14; 1.24] | 1.14 | [1.12; 1.17] |
| Severe Anemia |  |  |  |  |
| Urban^®^ | 1.00 |  | 1.00 |  |
| Rural | 1.08 | [0.99; 1.19] | 1.23 | [1.17; 1.29] |

Note: Estimates are derived from four-level (state, district, cluster, individual) random effects models. 95% CI – 95% Confidence Interval.

Figure S3: Dot plots showing distribution of standard deviations (SDs) of (A) Mild Anemia (B) Moderate Anemia; (C) Severe Anemia among Women and Children within districts. Note: Red lines represent median SD and blue lines represent 25th and 75th percentiles of SD.


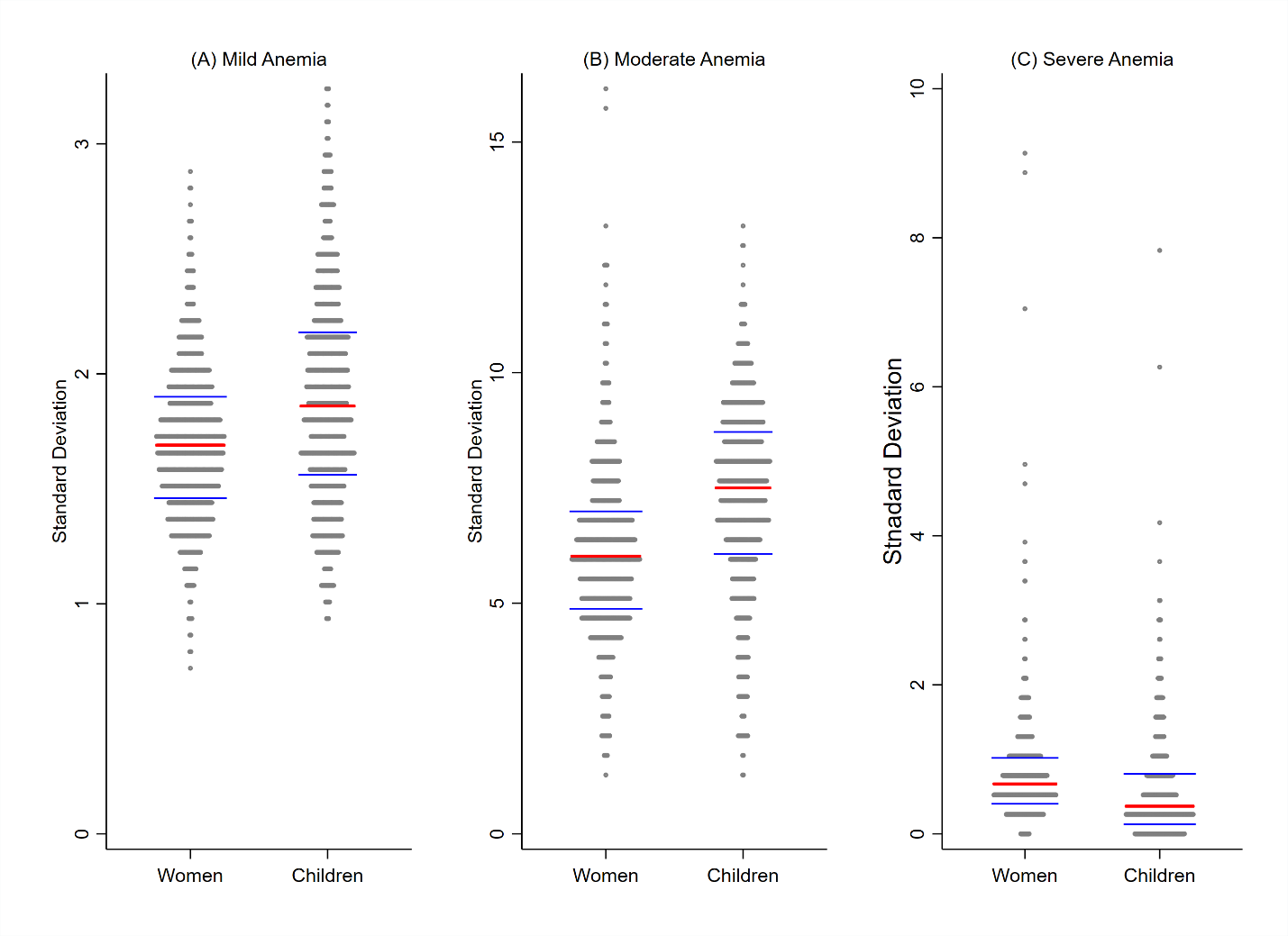


Table S3**:** Correlation (r) between district-level prevalence and within-district,

between small areas standard deviation of anemia among children by states, NFHS 2021, India

| State | No. of Districts | Mild Anemia (r) | Moderate Anemia (r) | Severe Anemia (r) |
| --- | --- | --- | --- | --- |
| Andhra Pradesh | 13 | 0.52 | 0.65 | 0.91 |
| Arunachal Pradesh | 20 | 0.76 | 0.81 | 0.93 |
| Assam | 33 | 0.46 | 0.55 | 0.95 |
| Bihar | 38 | 0.54 | 0.36 | 0.84 |
| Chhattisgarh | 27 | 0.53 | 0.59 | 0.95 |
| Gujarat | 33 | 0.69 | 0.17 | 0.87 |
| Haryana | 22 | 0.38 | 0.66 | 0.95 |
| Himachal Pradesh | 12 | 0.65 | 0.82 | 0.93 |
| Jammu & Kashmir | 20 | 0.69 | 0.74 | 0.83 |
| Jharkhand | 24 | 0.23 | 0.79 | 0.91 |
| Karnataka | 30 | 0.42 | 0.8 | 0.93 |
| Kerala | 14 | 0.83 | 0.68 | 0.92 |
| Madhya Pradesh | 51 | 0.51 | 0.47 | 0.81 |
| Maharashtra | 36 | 0.44 | 0.58 | 0.71 |
| Manipur | 9 | 0.91 | 0.96 | 0.82 |
| Meghalaya | 11 | 0.74 | 0.97 | 0.89 |
| Mizoram | 8 | 0.49 | 0.88 | 0.93 |
| Nagaland | 11 | 0.75 | 0.87 | 0.92 |
| Dadra & N; Daman & Diu | 3 | -0.45 | 0.78 | 0.99 |
| NCT Delhi | 11 | 0.81 | 0.67 | 0.93 |
| Odisha | 30 | 0.66 | 0.78 | 0.77 |
| Puducherry | 4 | 0.13 | 0.97 | 0.98 |
| Punjab | 22 | 0.72 | 0.41 | 0.91 |
| Rajasthan | 33 | 0.39 | 0.32 | 0.86 |
| Sikkim | 4 | 0.72 | 0.86 | 0.96 |
| Tamil Nadu | 32 | 0.63 | 0.81 | 0.82 |
| Telangana | 31 | 0.31 | 0.39 | 0.93 |
| Tripura | 8 | 0.65 | 0.61 | 0.94 |
| Uttar Pradesh | 75 | 0.35 | 0.69 | 0.85 |
| Uttarakhand | 13 | 0.45 | 0.88 | 0.97 |
| West Bengal | 20 | 0.34 | 0.81 | 0.91 |

Table S4: Correlation (r) between district-level prevalence and within-district,

between small areas standard deviation of anemia among women by states, NFHS 2021, India

| State | No. of Districts | Mild Anemia (r) | Moderate Anemia (r) | Severe Anemia (r) |
| --- | --- | --- | --- | --- |
| Andhra Pradesh | 13 | 0.45 | -0.06 | 0.84 |
| Arunachal Pradesh | 20 | 0.52 | 0.85 | 0.75 |
| Assam | 33 | 0.35 | 0.49 | 0.68 |
| Bihar | 38 | 0.51 | 0.37 | 0.71 |
| Chhattisgarh | 27 | 0.53 | 0.65 | 0.93 |
| Gujarat | 33 | 0.26 | 0.71 | 0.90 |
| Haryana | 22 | 0.23 | 0.65 | 0.87 |
| Himachal Pradesh | 12 | -0.03 | 0.65 | 0.99 |
| Jammu & Kashmir | 20 | 0.53 | 0.55 | 0.84 |
| Jharkhand | 24 | 0.37 | 0.35 | 0.91 |
| Karnataka | 30 | 0.72 | 0.85 | 0.89 |
| Kerala | 14 | 0.37 | 0.77 | 0.94 |
| Madhya Pradesh | 51 | 0.52 | 0.61 | 0.86 |
| Maharashtra | 36 | 0.53 | 0.61 | 0.94 |
| Manipur | 9 | 0.52 | 0.75 | 0.96 |
| Meghalaya | 11 | 0.68 | 0.69 | 0.95 |
| Mizoram | 8 | 0.87 | 0.95 | 0.95 |
| Nagaland | 11 | 0.52 | 0.68 | 0.96 |
| Dadra & N; Daman & Diu | 3 | 0.86 | 0.99 | 0.93 |
| NCT Delhi | 11 | 0.13 | 0.65 | 0.76 |
| Odisha | 30 | 0.16 | 0.63 | 0.91 |
| Puducherry | 4 | 0.5 | -0.29 | 0.98 |
| Punjab | 22 | 0.19 | 0.69 | 0.87 |
| Rajasthan | 33 | 0.45 | 0.47 | 0.91 |
| Sikkim | 4 | -0.13 | 0.49 | 0.88 |
| Tamil Nadu | 32 | 0.54 | 0.61 | 0.96 |
| Telangana | 31 | 0.62 | 0.7 | 0.98 |
| Tripura | 8 | 0.41 | 0.69 | 0.88 |
| Uttar Pradesh | 75 | 0.32 | 0.65 | 0.89 |
| Uttarakhand | 13 | 0.46 | 0.86 | 0.86 |
| West Bengal | 20 | 0.26 | 0.16 | 0.93 |

Table S5: Distribution of districts based on the district level percent and within district, between small areas standard deviation of Anemia among Children and Women in India, NFHS 2021

| Mild Anemia - Children | Standard Deviation | | | |
| --- | --- | --- | --- | --- |
| Mean Prevalence | Low | Medium | High | Total |
| Low | 157 | 62 | 19 | 238 |
| medium | 64 | 102 | 68 | 234 |
| High | 21 | 71 | 143 | 235 |
| Total | 242 | 235 | 230 | 707 |
| Moderate Anemia - Children |  |  |  |  |
| Mean Prevalence | Low | Medium | High | Total |
| Low | 177 | 44 | 15 | 236 |
| medium | 44 | 107 | 86 | 237 |
| High | 16 | 85 | 133 | 234 |
| Total | 237 | 236 | 234 | 707 |
| Mild Anemia - Children |  |  |  |  |
| Severe Prevalence | Low | Medium | High | Total |
| Low | 202 | 33 | 1 | 236 |
| medium | 38 | 160 | 39 | 237 |
| High | 0 | 45 | 189 | 234 |
| Total | 240 | 238 | 229 | 707 |
|  | | | | |
| Mild Anemia - Women | Standard Deviation | | | |
| Mean Prevalence | Low | Medium | High | Total |
| Low | 156 | 57 | 23 | 236 |
| medium | 70 | 101 | 65 | 236 |
| High | 19 | 70 | 146 | 235 |
| Total | 245 | 228 | 234 | 707 |
| Moderate Anemia - Women |  |  |  |  |
| Mean Prevalence | Low | Medium | High | Total |
| Low | 179 | 48 | 10 | 237 |
| medium | 50 | 115 | 70 | 235 |
| High | 7 | 73 | 155 | 235 |
| Total | 236 | 236 | 235 | 707 |
| Mild Anemia - Women |  |  |  |  |
| Severe Prevalence | Low | Medium | High | Total |
| Low | 203 | 31 | 3 | 237 |
| medium | 33 | 168 | 35 | 236 |
| High | 0 | 38 | 196 | 234 |
| Total | 236 | 237 | 234 | 707 |

Table S6: Mean Prevalence and Standard Deviation of Anemia (Any and Severe) among children (6-59 months) Failures by Districts, India, NFHS, 2021

| DISTRICT NAME | STATE NAME | Mild Anemia - Prevalence (%) | Decile Position (Mild Anemia) | Mild Anemia - SD | Decile Position (Mild Anemia SD) | Moderate Anemia - Prevalence (%) | Decile Position (Moderate Anemia) | Moderate Anemia - SD | Decile Position (Moderate Anemia SD) | Severe Anemia -Prevalence (%) | Decile Position (Severe Anemia) | Severe Anemia - Sd | Decile Position (Severe Anemia SD) |
| --- | --- | --- | --- | --- | --- | --- | --- | --- | --- | --- | --- | --- | --- |
| KUPWARA | JAMMU & KASHMIR | 27.33 | 5 | 2.70 | 5 | 35.0 | 5 | 9.29 | 7 | 0.96 | 5 | 0.6 | 7 |
| BADGAM | JAMMU & KASHMIR | 31.26 | 8 | 2.63 | 8 | 36.3 | 7 | 9.88 | 10 | 1.49 | 7 | 1.9 | 10 |
| LEH(LADAKH) | LADAKH | 26.69 | 5 | 1.66 | 5 | 29.7 | 4 | 8.10 | 7 | 0.84 | 4 | 0.7 | 7 |
| KARGIL | LADAKH | 27.49 | 5 | 2.17 | 5 | 35.2 | 2 | 10.06 | 2 | 0.56 | 2 | 0.1 | 2 |
| PUNCH | JAMMU & KASHMIR | 26.95 | 5 | 2.04 | 5 | 26.9 | 4 | 9.38 | 5 | 0.81 | 4 | 0.3 | 5 |
| RAJOURI | JAMMU & KASHMIR | 26.62 | 5 | 2.36 | 5 | 47.6 | 7 | 12.75 | 9 | 1.53 | 7 | 1.2 | 9 |
| KATHUA | JAMMU & KASHMIR | 27.49 | 5 | 1.80 | 5 | 33.6 | 6 | 7.22 | 5 | 1.14 | 6 | 0.4 | 5 |
| BARAMULA | JAMMU & KASHMIR | 24.06 | 2 | 2.13 | 2 | 36.1 | 8 | 9.51 | 9 | 1.67 | 8 | 1.2 | 9 |
| BANDIPORE | JAMMU & KASHMIR | 22.86 | 1 | 1.43 | 1 | 34.3 | 7 | 9.58 | 9 | 1.53 | 7 | 1.1 | 9 |
| SRINAGAR | JAMMU & KASHMIR | 25.45 | 3 | 1.32 | 3 | 30.4 | 5 | 8.36 | 5 | 1.03 | 5 | 0.4 | 5 |
| GANDERBAL | JAMMU & KASHMIR | 21.49 | 1 | 1.23 | 1 | 45.0 | 7 | 10.49 | 10 | 1.36 | 7 | 2.2 | 10 |
| PULWAMA | JAMMU & KASHMIR | 30.37 | 8 | 2.52 | 8 | 25.8 | 4 | 6.75 | 5 | 0.81 | 4 | 0.4 | 5 |
| SHUPIYAN | JAMMU & KASHMIR | 25.88 | 4 | 2.07 | 4 | 28.9 | 5 | 9.82 | 5 | 0.94 | 5 | 0.3 | 5 |
| ANANTNAG | JAMMU & KASHMIR | 30.60 | 8 | 2.09 | 8 | 33.0 | 4 | 9.07 | 3 | 0.76 | 4 | 0.2 | 3 |
| KULGAM | JAMMU & KASHMIR | 35.77 | 10 | 2.42 | 10 | 25.7 | 3 | 7.19 | 3 | 0.72 | 3 | 0.2 | 3 |
| DODA | JAMMU & KASHMIR | 26.04 | 4 | 1.85 | 4 | 38.4 | 3 | 10.00 | 2 | 0.71 | 3 | 0.1 | 2 |
| RAMBAN | JAMMU & KASHMIR | 25.77 | 3 | 1.92 | 3 | 30.2 | 3 | 10.29 | 3 | 0.67 | 3 | 0.1 | 3 |
| KISHTWAR | JAMMU & KASHMIR | 25.08 | 3 | 1.56 | 3 | 37.9 | 4 | 9.90 | 3 | 0.77 | 4 | 0.1 | 3 |
| UDHAMPUR | JAMMU & KASHMIR | 24.79 | 2 | 1.51 | 2 | 37.8 | 9 | 10.24 | 10 | 2.32 | 9 | 1.8 | 10 |
| REASI | JAMMU & KASHMIR | 28.07 | 6 | 2.42 | 6 | 23.2 | 4 | 6.46 | 4 | 0.77 | 4 | 0.2 | 4 |
| JAMMU | JAMMU & KASHMIR | 27.49 | 5 | 1.92 | 5 | 31.3 | 5 | 6.88 | 7 | 1.09 | 5 | 0.6 | 7 |
| SAMBA | JAMMU & KASHMIR | 24.18 | 2 | 1.87 | 2 | 41.4 | 4 | 9.19 | 4 | 0.81 | 4 | 0.2 | 4 |
| CHAMBA | HIMACHAL PRADESH | 23.13 | 1 | 1.40 | 1 | 10.5 | 3 | 2.20 | 1 | 0.60 | 3 | 0.0 | 1 |
| KANGRA | HIMACHAL PRADESH | 24.12 | 2 | 1.20 | 2 | 22.5 | 4 | 5.49 | 4 | 0.85 | 4 | 0.2 | 4 |
| LAHUL & SPITI | HIMACHAL PRADESH | 21.02 | 1 | 1.18 | 1 | 16.5 | 4 | 3.05 | 4 | 0.84 | 4 | 0.2 | 4 |
| KULLU | HIMACHAL PRADESH | 25.81 | 4 | 1.45 | 4 | 25.4 | 4 | 6.83 | 6 | 0.84 | 4 | 0.4 | 6 |
| MANDI | HIMACHAL PRADESH | 24.69 | 2 | 1.78 | 2 | 15.8 | 6 | 3.81 | 6 | 1.18 | 6 | 0.4 | 6 |
| HAMIRPUR | HIMACHAL PRADESH | 25.40 | 3 | 1.37 | 3 | 21.5 | 3 | 4.84 | 1 | 0.66 | 3 | 0.0 | 1 |
| UNA | HIMACHAL PRADESH | 24.01 | 2 | 1.32 | 2 | 25.9 | 8 | 5.28 | 9 | 1.55 | 8 | 1.2 | 9 |
| BILASPUR | HIMACHAL PRADESH | 23.75 | 2 | 1.13 | 2 | 17.3 | 4 | 3.32 | 4 | 0.84 | 4 | 0.2 | 4 |
| SOLAN | HIMACHAL PRADESH | 24.89 | 2 | 1.70 | 2 | 20.0 | 5 | 4.86 | 6 | 1.03 | 5 | 0.4 | 6 |
| SIRMAUR | HIMACHAL PRADESH | 26.72 | 5 | 2.10 | 5 | 20.7 | 7 | 7.31 | 7 | 1.34 | 7 | 0.7 | 7 |
| SHIMLA | HIMACHAL PRADESH | 22.90 | 1 | 1.44 | 1 | 20.0 | 6 | 5.63 | 6 | 1.15 | 6 | 0.4 | 6 |
| KINNAUR | HIMACHAL PRADESH | 23.92 | 2 | 1.35 | 2 | 14.0 | 3 | 3.18 | 1 | 0.67 | 3 | 0.0 | 1 |
| KAPURTHALA | PUNJAB | 23.11 | 1 | 1.22 | 1 | 43.1 | 9 | 6.22 | 8 | 2.28 | 9 | 0.8 | 8 |
| JALANDHAR | PUNJAB | 25.24 | 3 | 1.42 | 3 | 39.5 | 10 | 6.45 | 9 | 3.30 | 10 | 1.3 | 9 |
| HOSHIARPUR | PUNJAB | 25.09 | 3 | 1.63 | 3 | 38.2 | 10 | 7.07 | 10 | 3.02 | 10 | 1.9 | 10 |
| SHAHID BHAGAT SINGH NAGAR | PUNJAB | 25.31 | 3 | 1.32 | 3 | 35.4 | 9 | 6.94 | 6 | 2.03 | 9 | 0.5 | 6 |
| FATEHGARH SAHIB | PUNJAB | 26.51 | 4 | 1.73 | 4 | 32.6 | 9 | 6.10 | 7 | 2.31 | 9 | 0.7 | 7 |
| LUDHIANA | PUNJAB | 25.51 | 3 | 1.54 | 3 | 43.0 | 10 | 7.24 | 10 | 3.97 | 10 | 2.2 | 10 |
| MOGA | PUNJAB | 26.75 | 5 | 1.64 | 5 | 34.0 | 10 | 7.54 | 10 | 4.24 | 10 | 3.1 | 10 |
| MUKTSAR | PUNJAB | 27.21 | 5 | 2.01 | 5 | 37.1 | 10 | 6.88 | 10 | 2.60 | 10 | 1.7 | 10 |
| FARIDKOT | PUNJAB | 26.52 | 4 | 1.90 | 4 | 40.1 | 10 | 8.06 | 10 | 3.11 | 10 | 2.0 | 10 |
| BATHINDA | PUNJAB | 23.86 | 2 | 1.09 | 2 | 37.7 | 10 | 7.40 | 10 | 4.13 | 10 | 1.9 | 10 |
| MANSA | PUNJAB | 26.61 | 5 | 1.80 | 5 | 41.2 | 10 | 6.82 | 10 | 3.76 | 10 | 2.4 | 10 |
| PATIALA | PUNJAB | 22.98 | 1 | 1.39 | 1 | 46.5 | 10 | 6.39 | 10 | 3.74 | 10 | 1.8 | 10 |
| AMRITSAR | PUNJAB | 25.97 | 4 | 1.71 | 4 | 40.9 | 10 | 8.79 | 9 | 2.96 | 10 | 1.4 | 9 |
| TARN TARAN | PUNJAB | 24.59 | 2 | 1.78 | 2 | 40.1 | 10 | 9.02 | 10 | 3.00 | 10 | 1.8 | 10 |
| RUPNAGAR | PUNJAB | 30.28 | 8 | 1.82 | 8 | 28.8 | 10 | 5.69 | 8 | 2.50 | 10 | 0.8 | 8 |
| SAHIBZADA AJIT SINGH NAGAR | PUNJAB | 24.26 | 2 | 1.18 | 2 | 34.7 | 10 | 6.58 | 10 | 3.04 | 10 | 1.7 | 10 |
| SANGRUR | PUNJAB | 25.23 | 3 | 1.27 | 3 | 36.3 | 10 | 7.07 | 9 | 3.01 | 10 | 1.2 | 9 |
| BARNALA | PUNJAB | 26.54 | 4 | 1.80 | 4 | 28.8 | 10 | 5.97 | 9 | 3.02 | 10 | 1.5 | 9 |
| CHANDIGARH | CHANDIGARH | 26.24 | 4 | 1.39 | 4 | 23.8 | 4 | 4.51 | 4 | 0.86 | 4 | 0.2 | 4 |
| UTTARKASHI | UTTARAKHAND | 25.24 | 3 | 1.53 | 3 | 25.3 | 5 | 7.25 | 6 | 1.05 | 5 | 0.4 | 6 |
| CHAMOLI | UTTARAKHAND | 26.62 | 5 | 1.93 | 5 | 18.3 | 3 | 5.08 | 1 | 0.59 | 3 | 0.0 | 1 |
| RUDRAPRAYAG | UTTARAKHAND | 21.70 | 1 | 1.43 | 1 | 19.6 | 5 | 5.19 | 5 | 1.03 | 5 | 0.4 | 5 |
| TEHRI GARHWAL | UTTARAKHAND | 24.23 | 2 | 1.66 | 2 | 20.6 | 3 | 4.80 | 3 | 0.73 | 3 | 0.2 | 3 |
| DEHRADUN | UTTARAKHAND | 23.21 | 1 | 1.09 | 1 | 28.5 | 6 | 5.96 | 6 | 1.16 | 6 | 0.4 | 6 |
| GARHWAL | UTTARAKHAND | 23.59 | 2 | 1.53 | 2 | 21.5 | 3 | 4.29 | 2 | 0.73 | 3 | 0.1 | 2 |
| PITHORAGARH | UTTARAKHAND | 21.18 | 1 | 1.33 | 1 | 12.2 | 4 | 2.18 | 3 | 0.77 | 4 | 0.2 | 3 |
| BAGESHWAR | UTTARAKHAND | 19.44 | 1 | 1.20 | 1 | 15.9 | 4 | 4.83 | 4 | 0.88 | 4 | 0.3 | 4 |
| ALMORA | UTTARAKHAND | 25.01 | 3 | 1.47 | 3 | 21.9 | 3 | 5.37 | 3 | 0.71 | 3 | 0.2 | 3 |
| CHAMPAWAT | UTTARAKHAND | 22.16 | 1 | 1.97 | 1 | 13.1 | 3 | 3.13 | 2 | 0.63 | 3 | 0.1 | 2 |
| NAINITAL | UTTARAKHAND | 25.79 | 4 | 1.46 | 4 | 25.4 | 4 | 6.42 | 6 | 0.89 | 4 | 0.4 | 6 |
| UDHAM SINGH NAGAR | UTTARAKHAND | 23.60 | 2 | 1.47 | 2 | 29.5 | 7 | 6.43 | 8 | 1.38 | 7 | 0.7 | 8 |
| HARDWAR | UTTARAKHAND | 24.43 | 2 | 1.58 | 2 | 29.3 | 7 | 7.87 | 8 | 1.48 | 7 | 0.8 | 8 |
| PANCHKULA | HARYANA | 25.71 | 3 | 1.22 | 3 | 30.9 | 7 | 6.78 | 5 | 1.50 | 7 | 0.3 | 5 |
| AMBALA | HARYANA | 24.18 | 2 | 1.58 | 2 | 37.2 | 8 | 8.34 | 7 | 1.74 | 8 | 0.6 | 7 |
| YAMUNANAGAR | HARYANA | 25.43 | 3 | 1.93 | 3 | 40.4 | 10 | 9.99 | 10 | 3.29 | 10 | 2.2 | 10 |
| KURUKSHETRA | HARYANA | 26.40 | 4 | 1.65 | 4 | 42.0 | 9 | 8.06 | 8 | 2.28 | 9 | 0.9 | 8 |
| KAITHAL | HARYANA | 20.44 | 1 | 1.11 | 1 | 42.7 | 9 | 7.38 | 7 | 1.90 | 9 | 0.7 | 7 |
| KARNAL | HARYANA | 28.09 | 6 | 2.34 | 6 | 42.1 | 10 | 7.22 | 10 | 2.90 | 10 | 2.0 | 10 |
| PANIPAT | HARYANA | 26.15 | 4 | 2.02 | 4 | 40.6 | 10 | 8.29 | 10 | 2.96 | 10 | 1.7 | 10 |
| SONIPAT | HARYANA | 26.47 | 4 | 1.86 | 4 | 39.6 | 8 | 8.25 | 6 | 1.68 | 8 | 0.5 | 6 |
| JIND | HARYANA | 24.13 | 2 | 2.14 | 2 | 40.1 | 9 | 7.59 | 8 | 2.39 | 9 | 0.8 | 8 |
| FATEHABAD | HARYANA | 25.14 | 3 | 1.66 | 3 | 33.1 | 8 | 6.32 | 7 | 1.72 | 8 | 0.6 | 7 |
| SIRSA | HARYANA | 24.68 | 2 | 1.21 | 2 | 39.9 | 9 | 7.67 | 8 | 2.29 | 9 | 0.8 | 8 |
| HISAR | HARYANA | 25.19 | 3 | 1.93 | 3 | 38.7 | 10 | 8.22 | 10 | 2.81 | 10 | 1.9 | 10 |
| ROHTAK | HARYANA | 28.14 | 6 | 1.78 | 6 | 36.5 | 10 | 6.40 | 10 | 4.01 | 10 | 2.5 | 10 |
| JHAJJAR | HARYANA | 25.90 | 4 | 2.26 | 4 | 40.1 | 9 | 8.41 | 9 | 2.02 | 9 | 1.1 | 9 |
| MAHENDRAGARH | HARYANA | 27.49 | 5 | 1.65 | 5 | 39.9 | 9 | 7.31 | 8 | 2.09 | 9 | 0.8 | 8 |
| REWARI | HARYANA | 25.90 | 4 | 1.68 | 4 | 44.1 | 9 | 10.50 | 8 | 2.10 | 9 | 0.8 | 8 |
| GURGAON | HARYANA | 28.92 | 7 | 1.62 | 7 | 32.8 | 9 | 4.99 | 7 | 1.98 | 9 | 0.6 | 7 |
| MEWAT | HARYANA | 25.17 | 3 | 1.77 | 3 | 46.3 | 9 | 10.51 | 9 | 2.35 | 9 | 1.3 | 9 |
| FARIDABAD | HARYANA | 27.49 | 5 | 1.77 | 5 | 37.6 | 8 | 7.62 | 6 | 1.66 | 8 | 0.5 | 6 |
| PALWAL | HARYANA | 25.18 | 3 | 2.01 | 3 | 42.6 | 8 | 9.24 | 7 | 1.79 | 8 | 0.6 | 7 |
| GANGANAGAR | RAJASTHAN | 30.06 | 8 | 1.60 | 8 | 37.9 | 7 | 8.26 | 7 | 1.52 | 7 | 0.7 | 7 |
| HANUMANGARH | RAJASTHAN | 31.15 | 8 | 2.40 | 8 | 43.6 | 7 | 8.84 | 9 | 1.47 | 7 | 1.1 | 9 |
| BIKANER | RAJASTHAN | 33.84 | 10 | 2.52 | 10 | 43.3 | 7 | 9.48 | 8 | 1.47 | 7 | 1.0 | 8 |
| CHURU | RAJASTHAN | 29.11 | 7 | 2.28 | 7 | 44.1 | 8 | 9.03 | 10 | 1.57 | 8 | 1.7 | 10 |
| JHUNJHUNUN | RAJASTHAN | 29.45 | 7 | 1.94 | 7 | 44.4 | 7 | 9.25 | 6 | 1.36 | 7 | 0.5 | 6 |
| ALWAR | RAJASTHAN | 26.92 | 5 | 1.76 | 5 | 32.9 | 9 | 6.48 | 9 | 2.39 | 9 | 1.4 | 9 |
| BHARATPUR | RAJASTHAN | 26.12 | 4 | 1.95 | 4 | 43.5 | 9 | 8.76 | 10 | 2.21 | 9 | 1.8 | 10 |
| DHAULPUR | RAJASTHAN | 28.18 | 6 | 2.52 | 6 | 47.8 | 10 | 9.35 | 9 | 2.55 | 10 | 1.5 | 9 |
| KARAULI | RAJASTHAN | 29.09 | 7 | 2.42 | 7 | 41.2 | 9 | 9.51 | 10 | 2.05 | 9 | 2.5 | 10 |
| SAWAI MADHOPUR | RAJASTHAN | 26.35 | 4 | 1.58 | 4 | 44.9 | 5 | 10.06 | 5 | 0.93 | 5 | 0.3 | 5 |
| DAUSA | RAJASTHAN | 29.56 | 7 | 1.92 | 7 | 41.7 | 7 | 9.34 | 6 | 1.43 | 7 | 0.5 | 6 |
| JAIPUR | RAJASTHAN | 31.41 | 9 | 2.52 | 9 | 33.9 | 8 | 7.96 | 8 | 1.83 | 8 | 0.7 | 8 |
| SIKAR | RAJASTHAN | 26.80 | 5 | 2.26 | 5 | 34.4 | 4 | 8.33 | 4 | 0.84 | 4 | 0.2 | 4 |
| NAGAUR | RAJASTHAN | 32.89 | 9 | 2.26 | 9 | 30.4 | 3 | 7.51 | 2 | 0.63 | 3 | 0.1 | 2 |
| JODHPUR | RAJASTHAN | 31.51 | 9 | 2.82 | 9 | 30.3 | 3 | 9.16 | 4 | 0.72 | 3 | 0.2 | 4 |
| JAISALMER | RAJASTHAN | 30.58 | 8 | 2.83 | 8 | 24.7 | 4 | 7.17 | 5 | 0.87 | 4 | 0.3 | 5 |
| BARMER | RAJASTHAN | 31.35 | 9 | 2.39 | 9 | 28.2 | 4 | 9.84 | 5 | 0.89 | 4 | 0.3 | 5 |
| JALOR | RAJASTHAN | 28.16 | 6 | 2.14 | 6 | 35.8 | 5 | 13.33 | 7 | 1.08 | 5 | 0.7 | 7 |
| SIROHI | RAJASTHAN | 28.42 | 6 | 1.92 | 6 | 48.4 | 5 | 10.26 | 5 | 1.06 | 5 | 0.4 | 5 |
| PALI | RAJASTHAN | 34.50 | 10 | 2.38 | 10 | 35.0 | 4 | 8.80 | 4 | 0.85 | 4 | 0.2 | 4 |
| AJMER | RAJASTHAN | 30.60 | 8 | 1.93 | 8 | 40.9 | 4 | 8.98 | 4 | 0.87 | 4 | 0.2 | 4 |
| TONK | RAJASTHAN | 25.66 | 3 | 1.67 | 3 | 39.9 | 8 | 9.69 | 9 | 1.82 | 8 | 1.2 | 9 |
| BUNDI | RAJASTHAN | 33.11 | 9 | 2.57 | 9 | 34.3 | 5 | 10.01 | 5 | 1.01 | 5 | 0.4 | 5 |
| BHILWARA | RAJASTHAN | 30.93 | 8 | 1.85 | 8 | 31.6 | 3 | 7.93 | 1 | 0.63 | 3 | 0.0 | 1 |
| RAJSAMAND | RAJASTHAN | 34.39 | 10 | 1.97 | 10 | 43.9 | 4 | 8.23 | 3 | 0.82 | 4 | 0.2 | 3 |
| DUNGARPUR | RAJASTHAN | 31.07 | 8 | 2.16 | 8 | 45.1 | 3 | 9.53 | 2 | 0.67 | 3 | 0.1 | 2 |
| BANSWARA | RAJASTHAN | 30.82 | 8 | 2.25 | 8 | 43.2 | 4 | 11.19 | 4 | 0.86 | 4 | 0.2 | 4 |
| CHITTAURGARH | RAJASTHAN | 28.95 | 7 | 1.82 | 7 | 43.8 | 5 | 8.25 | 4 | 0.91 | 5 | 0.3 | 4 |
| KOTA | RAJASTHAN | 31.06 | 8 | 2.08 | 8 | 33.8 | 6 | 8.76 | 5 | 1.13 | 6 | 0.4 | 5 |
| BARAN | RAJASTHAN | 28.82 | 7 | 2.45 | 7 | 34.8 | 7 | 10.80 | 8 | 1.47 | 7 | 0.9 | 8 |
| JHALAWAR | RAJASTHAN | 33.64 | 10 | 2.17 | 10 | 37.3 | 4 | 8.74 | 6 | 0.84 | 4 | 0.4 | 6 |
| UDAIPUR | RAJASTHAN | 31.10 | 8 | 2.37 | 8 | 43.5 | 4 | 9.75 | 3 | 0.77 | 4 | 0.2 | 3 |
| PRATAPGARH | RAJASTHAN | 34.03 | 10 | 2.00 | 10 | 25.4 | 4 | 8.38 | 3 | 0.78 | 4 | 0.2 | 3 |
| SAHARANPUR | UTTAR PRADESH | 27.54 | 6 | 2.65 | 6 | 36.2 | 6 | 8.87 | 7 | 1.10 | 6 | 0.6 | 7 |
| BIJNOR | UTTAR PRADESH | 29.29 | 7 | 2.21 | 7 | 28.9 | 5 | 7.42 | 6 | 1.04 | 5 | 0.5 | 6 |
| RAMPUR | UTTAR PRADESH | 31.79 | 9 | 2.77 | 9 | 30.1 | 6 | 7.23 | 8 | 1.27 | 6 | 0.8 | 8 |
| JYOTIBA PHULE NAGAR | UTTAR PRADESH | 30.16 | 8 | 2.30 | 8 | 35.8 | 7 | 11.15 | 7 | 1.39 | 7 | 0.6 | 7 |
| MEERUT | UTTAR PRADESH | 27.57 | 6 | 1.68 | 6 | 25.1 | 7 | 5.70 | 8 | 1.37 | 7 | 0.8 | 8 |
| BAGHPAT | UTTAR PRADESH | 22.41 | 1 | 1.85 | 1 | 38.5 | 8 | 9.76 | 9 | 1.56 | 8 | 1.1 | 9 |
| GAUTAM BUDDHA NAGAR | UTTAR PRADESH | 25.58 | 3 | 1.73 | 3 | 36.7 | 7 | 9.20 | 8 | 1.48 | 7 | 0.7 | 8 |
| BULANDSHAHR | UTTAR PRADESH | 24.80 | 2 | 1.76 | 2 | 37.3 | 6 | 8.66 | 8 | 1.31 | 6 | 0.8 | 8 |
| ALIGARH | UTTAR PRADESH | 25.02 | 3 | 1.86 | 3 | 36.5 | 5 | 8.20 | 5 | 1.00 | 5 | 0.3 | 5 |
| MAHAMAYA NAGAR | UTTAR PRADESH | 24.98 | 3 | 1.98 | 3 | 44.2 | 9 | 9.09 | 8 | 2.13 | 9 | 0.9 | 8 |
| MATHURA | UTTAR PRADESH | 26.08 | 4 | 1.43 | 4 | 47.1 | 9 | 9.38 | 9 | 1.99 | 9 | 1.1 | 9 |
| AGRA | UTTAR PRADESH | 26.04 | 4 | 2.16 | 4 | 44.7 | 10 | 9.15 | 10 | 2.49 | 10 | 1.9 | 10 |
| FIROZABAD | UTTAR PRADESH | 24.06 | 2 | 1.66 | 2 | 46.0 | 7 | 8.22 | 8 | 1.45 | 7 | 0.8 | 8 |
| MAINPURI | UTTAR PRADESH | 28.58 | 6 | 2.44 | 6 | 38.1 | 6 | 8.98 | 7 | 1.22 | 6 | 0.7 | 7 |
| BAREILLY | UTTAR PRADESH | 28.05 | 6 | 2.52 | 6 | 39.4 | 6 | 10.02 | 5 | 1.12 | 6 | 0.3 | 5 |
| PILIBHIT | UTTAR PRADESH | 26.87 | 5 | 1.86 | 5 | 28.4 | 6 | 8.06 | 6 | 1.21 | 6 | 0.4 | 6 |
| SHAHJAHANPUR | UTTAR PRADESH | 28.85 | 7 | 2.74 | 7 | 40.8 | 9 | 10.30 | 10 | 2.00 | 9 | 1.9 | 10 |
| KHERI | UTTAR PRADESH | 27.72 | 6 | 2.20 | 6 | 34.0 | 7 | 8.03 | 9 | 1.49 | 7 | 1.2 | 9 |
| SITAPUR | UTTAR PRADESH | 29.59 | 7 | 1.86 | 7 | 33.3 | 8 | 5.90 | 8 | 1.62 | 8 | 0.9 | 8 |
| HARDOI | UTTAR PRADESH | 28.27 | 6 | 2.09 | 6 | 40.9 | 7 | 9.27 | 7 | 1.47 | 7 | 0.7 | 7 |
| UNNAO | UTTAR PRADESH | 28.44 | 6 | 2.01 | 6 | 36.8 | 9 | 9.27 | 9 | 1.94 | 9 | 1.3 | 9 |
| LUCKNOW | UTTAR PRADESH | 26.40 | 4 | 1.49 | 4 | 27.3 | 8 | 4.84 | 8 | 1.54 | 8 | 0.8 | 8 |
| FARRUKHABAD | UTTAR PRADESH | 28.78 | 7 | 2.52 | 7 | 42.8 | 6 | 9.73 | 6 | 1.13 | 6 | 0.4 | 6 |
| KANNAUJ | UTTAR PRADESH | 28.85 | 7 | 2.98 | 7 | 42.6 | 9 | 10.35 | 8 | 1.89 | 9 | 1.0 | 8 |
| ETAWAH | UTTAR PRADESH | 27.17 | 5 | 2.37 | 5 | 43.8 | 6 | 10.91 | 6 | 1.20 | 6 | 0.4 | 6 |
| AURAIYA | UTTAR PRADESH | 28.42 | 6 | 2.24 | 6 | 30.3 | 8 | 9.34 | 7 | 1.57 | 8 | 0.6 | 7 |
| KANPUR DEHAT | UTTAR PRADESH | 29.54 | 7 | 1.68 | 7 | 35.1 | 8 | 6.98 | 8 | 1.60 | 8 | 0.8 | 8 |
| KANPUR NAGAR | UTTAR PRADESH | 32.28 | 9 | 2.16 | 9 | 36.5 | 7 | 6.63 | 6 | 1.34 | 7 | 0.5 | 6 |
| JALAUN | UTTAR PRADESH | 25.30 | 3 | 1.85 | 3 | 26.9 | 5 | 6.83 | 5 | 1.04 | 5 | 0.3 | 5 |
| JHANSI | UTTAR PRADESH | 28.54 | 6 | 1.70 | 6 | 37.9 | 5 | 9.10 | 5 | 0.96 | 5 | 0.4 | 5 |
| LALITPUR | UTTAR PRADESH | 27.78 | 6 | 2.45 | 6 | 27.6 | 3 | 8.90 | 3 | 0.71 | 3 | 0.2 | 3 |
| HAMIRPUR | UTTAR PRADESH | 26.98 | 5 | 2.21 | 5 | 38.2 | 5 | 10.80 | 5 | 1.05 | 5 | 0.4 | 5 |
| MAHOBA | UTTAR PRADESH | 27.41 | 5 | 2.40 | 5 | 37.9 | 5 | 11.83 | 6 | 1.05 | 5 | 0.5 | 6 |
| BANDA | UTTAR PRADESH | 25.98 | 4 | 2.03 | 4 | 48.1 | 5 | 10.03 | 8 | 1.08 | 5 | 0.9 | 8 |
| CHITRAKOOT | UTTAR PRADESH | 27.04 | 5 | 2.33 | 5 | 28.7 | 5 | 7.64 | 5 | 1.00 | 5 | 0.3 | 5 |
| FATEHPUR | UTTAR PRADESH | 25.52 | 3 | 1.93 | 3 | 44.9 | 8 | 8.34 | 8 | 1.75 | 8 | 0.9 | 8 |
| PRATAPGARH | UTTAR PRADESH | 28.19 | 6 | 1.66 | 6 | 29.7 | 4 | 7.06 | 4 | 0.85 | 4 | 0.2 | 4 |
| KAUSHAMBI | UTTAR PRADESH | 33.20 | 9 | 2.10 | 9 | 28.4 | 4 | 6.20 | 3 | 0.83 | 4 | 0.2 | 3 |
| ALLAHABAD | UTTAR PRADESH | 27.87 | 6 | 1.74 | 6 | 21.8 | 4 | 5.09 | 3 | 0.76 | 4 | 0.2 | 3 |
| BARA BANKI | UTTAR PRADESH | 27.18 | 5 | 2.12 | 5 | 34.0 | 9 | 6.85 | 9 | 1.88 | 9 | 1.2 | 9 |
| FAIZABAD | UTTAR PRADESH | 28.16 | 6 | 2.21 | 6 | 27.1 | 4 | 6.31 | 2 | 0.75 | 4 | 0.1 | 2 |
| AMBEDKAR NAGAR | UTTAR PRADESH | 27.87 | 6 | 1.47 | 6 | 25.5 | 6 | 6.11 | 5 | 1.17 | 6 | 0.4 | 5 |
| BAHRAICH | UTTAR PRADESH | 24.79 | 2 | 2.13 | 2 | 40.8 | 8 | 10.81 | 8 | 1.65 | 8 | 0.8 | 8 |
| SHRAWASTI | UTTAR PRADESH | 22.80 | 1 | 1.96 | 1 | 38.5 | 8 | 9.65 | 9 | 1.83 | 8 | 1.5 | 9 |
| BALRAMPUR | UTTAR PRADESH | 27.41 | 5 | 2.17 | 5 | 44.8 | 8 | 10.10 | 10 | 1.72 | 8 | 1.7 | 10 |
| GONDA | UTTAR PRADESH | 24.61 | 2 | 2.16 | 2 | 34.7 | 9 | 8.86 | 9 | 2.44 | 9 | 1.3 | 9 |
| SIDDHARTHNAGAR | UTTAR PRADESH | 30.26 | 8 | 2.21 | 8 | 44.6 | 6 | 10.84 | 8 | 1.24 | 6 | 1.0 | 8 |
| BASTI | UTTAR PRADESH | 28.20 | 6 | 2.02 | 6 | 27.6 | 6 | 7.95 | 8 | 1.11 | 6 | 0.7 | 8 |
| SANT KABIR NAGAR | UTTAR PRADESH | 29.30 | 7 | 2.60 | 7 | 38.8 | 7 | 9.55 | 7 | 1.34 | 7 | 0.7 | 7 |
| MAHRAJGANJ | UTTAR PRADESH | 27.33 | 5 | 2.05 | 5 | 34.1 | 4 | 9.33 | 4 | 0.83 | 4 | 0.2 | 4 |
| GORAKHPUR | UTTAR PRADESH | 30.98 | 8 | 2.29 | 8 | 34.8 | 4 | 9.93 | 4 | 0.89 | 4 | 0.3 | 4 |
| KUSHINAGAR | UTTAR PRADESH | 30.01 | 8 | 2.59 | 8 | 21.1 | 3 | 5.47 | 3 | 0.73 | 3 | 0.2 | 3 |
| DEORIA | UTTAR PRADESH | 29.66 | 7 | 1.65 | 7 | 25.4 | 3 | 7.03 | 1 | 0.66 | 3 | 0.0 | 1 |
| AZAMGARH | UTTAR PRADESH | 28.13 | 6 | 1.71 | 6 | 29.3 | 5 | 6.72 | 8 | 1.07 | 5 | 0.9 | 8 |
| MAU | UTTAR PRADESH | 27.08 | 5 | 2.09 | 5 | 32.0 | 5 | 9.15 | 5 | 0.93 | 5 | 0.3 | 5 |
| BALLIA | UTTAR PRADESH | 32.37 | 9 | 1.78 | 9 | 34.5 | 4 | 7.96 | 4 | 0.83 | 4 | 0.2 | 4 |
| JAUNPUR | UTTAR PRADESH | 30.37 | 8 | 2.03 | 8 | 32.9 | 4 | 8.93 | 4 | 0.80 | 4 | 0.2 | 4 |
| GHAZIPUR | UTTAR PRADESH | 31.70 | 9 | 1.92 | 9 | 34.3 | 4 | 8.44 | 4 | 0.87 | 4 | 0.2 | 4 |
| CHANDAULI | UTTAR PRADESH | 29.71 | 8 | 1.80 | 8 | 33.5 | 5 | 6.70 | 7 | 1.02 | 5 | 0.7 | 7 |
| VARANASI | UTTAR PRADESH | 25.86 | 4 | 1.68 | 4 | 35.5 | 6 | 9.52 | 7 | 1.31 | 6 | 0.6 | 7 |
| SANT RAVIDAS NAGAR (BHADOHI) | UTTAR PRADESH | 24.11 | 2 | 1.81 | 2 | 32.2 | 8 | 6.84 | 9 | 1.86 | 8 | 1.1 | 9 |
| MIRZAPUR | UTTAR PRADESH | 25.69 | 3 | 1.68 | 3 | 30.6 | 6 | 7.80 | 6 | 1.24 | 6 | 0.4 | 6 |
| SONBHADRA | UTTAR PRADESH | 32.89 | 9 | 2.71 | 9 | 27.8 | 4 | 8.28 | 3 | 0.77 | 4 | 0.2 | 3 |
| ETAH | UTTAR PRADESH | 26.08 | 4 | 1.95 | 4 | 48.4 | 7 | 10.30 | 7 | 1.45 | 7 | 0.7 | 7 |
| KANSHIRAM NAGAR | UTTAR PRADESH | 28.07 | 6 | 2.45 | 6 | 47.0 | 9 | 9.42 | 9 | 2.16 | 9 | 1.1 | 9 |
| PASHCHIM CHAMPARAN | BIHAR | 28.39 | 6 | 2.11 | 6 | 33.3 | 5 | 8.55 | 6 | 1.01 | 5 | 0.5 | 6 |
| PURBA CHAMPARAN | BIHAR | 30.86 | 8 | 2.29 | 8 | 31.5 | 5 | 6.97 | 6 | 1.08 | 5 | 0.5 | 6 |
| SHEOHAR | BIHAR | 35.14 | 10 | 2.88 | 10 | 35.1 | 3 | 7.50 | 2 | 0.63 | 3 | 0.1 | 2 |
| SITAMARHI | BIHAR | 30.24 | 8 | 2.07 | 8 | 36.0 | 8 | 8.84 | 8 | 1.61 | 8 | 1.0 | 8 |
| MADHUBANI | BIHAR | 30.33 | 8 | 2.66 | 8 | 38.3 | 3 | 9.03 | 2 | 0.61 | 3 | 0.1 | 2 |
| SUPAUL | BIHAR | 30.95 | 8 | 2.86 | 8 | 31.6 | 3 | 7.93 | 4 | 0.69 | 3 | 0.2 | 4 |
| ARARIA | BIHAR | 32.16 | 9 | 3.06 | 9 | 39.2 | 6 | 9.54 | 9 | 1.17 | 6 | 1.0 | 9 |
| KISHANGANJ | BIHAR | 28.67 | 7 | 2.30 | 7 | 34.7 | 8 | 10.07 | 9 | 1.63 | 8 | 1.2 | 9 |
| PURNIA | BIHAR | 28.10 | 6 | 2.04 | 6 | 40.7 | 4 | 7.99 | 4 | 0.83 | 4 | 0.3 | 4 |
| KATIHAR | BIHAR | 28.50 | 6 | 2.36 | 6 | 38.4 | 5 | 11.28 | 5 | 0.98 | 5 | 0.3 | 5 |
| MADHEPURA | BIHAR | 27.72 | 6 | 2.40 | 6 | 40.6 | 6 | 10.66 | 6 | 1.12 | 6 | 0.4 | 6 |
| SAHARSA | BIHAR | 29.87 | 8 | 2.71 | 8 | 37.9 | 7 | 9.34 | 7 | 1.36 | 7 | 0.6 | 7 |
| DARBHANGA | BIHAR | 30.07 | 8 | 2.17 | 8 | 36.2 | 6 | 6.96 | 7 | 1.25 | 6 | 0.7 | 7 |
| MUZAFFARPUR | BIHAR | 29.48 | 7 | 2.30 | 7 | 33.6 | 6 | 5.90 | 7 | 1.27 | 6 | 0.6 | 7 |
| GOPALGANJ | BIHAR | 28.81 | 7 | 2.23 | 7 | 28.3 | 5 | 8.31 | 7 | 1.06 | 5 | 0.6 | 7 |
| SIWAN | BIHAR | 29.46 | 7 | 1.83 | 7 | 34.9 | 6 | 5.99 | 6 | 1.21 | 6 | 0.4 | 6 |
| SARAN | BIHAR | 29.00 | 7 | 2.39 | 7 | 39.8 | 6 | 9.43 | 7 | 1.26 | 6 | 0.6 | 7 |
| VAISHALI | BIHAR | 28.50 | 6 | 1.80 | 6 | 47.2 | 5 | 7.94 | 5 | 0.93 | 5 | 0.3 | 5 |
| SAMASTIPUR | BIHAR | 29.59 | 7 | 2.08 | 7 | 36.8 | 3 | 7.57 | 3 | 0.70 | 3 | 0.2 | 3 |
| BEGUSARAI | BIHAR | 30.86 | 8 | 2.11 | 8 | 40.6 | 6 | 8.32 | 6 | 1.14 | 6 | 0.5 | 6 |
| KHAGARIA | BIHAR | 28.58 | 6 | 2.15 | 6 | 38.2 | 4 | 10.04 | 5 | 0.80 | 4 | 0.3 | 5 |
| BHAGALPUR | BIHAR | 29.20 | 7 | 2.47 | 7 | 45.0 | 5 | 8.99 | 6 | 0.91 | 5 | 0.5 | 6 |
| BANKA | BIHAR | 31.31 | 9 | 2.26 | 9 | 42.8 | 6 | 8.36 | 7 | 1.30 | 6 | 0.6 | 7 |
| MUNGER | BIHAR | 29.15 | 7 | 2.10 | 7 | 42.6 | 6 | 7.90 | 8 | 1.25 | 6 | 0.9 | 8 |
| LAKHISARAI | BIHAR | 31.02 | 8 | 2.24 | 8 | 45.7 | 6 | 10.10 | 6 | 1.18 | 6 | 0.6 | 6 |
| SHEIKHPURA | BIHAR | 33.80 | 10 | 2.77 | 10 | 41.9 | 5 | 9.31 | 5 | 0.94 | 5 | 0.3 | 5 |
| NALANDA | BIHAR | 33.92 | 10 | 1.98 | 10 | 41.6 | 4 | 9.12 | 7 | 0.82 | 4 | 0.7 | 7 |
| PATNA | BIHAR | 29.96 | 8 | 2.07 | 8 | 31.3 | 7 | 6.88 | 8 | 1.43 | 7 | 0.9 | 8 |
| BHOJPUR | BIHAR | 33.67 | 10 | 2.95 | 10 | 30.6 | 3 | 8.00 | 3 | 0.68 | 3 | 0.2 | 3 |
| BUXAR | BIHAR | 33.02 | 9 | 2.52 | 9 | 32.8 | 3 | 10.59 | 3 | 0.71 | 3 | 0.2 | 3 |
| KAIMUR (BHABUA) | BIHAR | 32.29 | 9 | 2.77 | 9 | 36.1 | 7 | 9.02 | 7 | 1.34 | 7 | 0.6 | 7 |
| ROHTAS | BIHAR | 29.17 | 7 | 2.49 | 7 | 39.5 | 6 | 7.38 | 6 | 1.31 | 6 | 0.5 | 6 |
| AURANGABAD | BIHAR | 33.69 | 10 | 2.05 | 10 | 34.0 | 3 | 8.20 | 2 | 0.57 | 3 | 0.1 | 2 |
| GAYA | BIHAR | 35.11 | 10 | 2.86 | 10 | 38.2 | 5 | 8.98 | 7 | 1.04 | 5 | 0.7 | 7 |
| NAWADA | BIHAR | 28.87 | 7 | 2.59 | 7 | 44.6 | 7 | 9.74 | 9 | 1.48 | 7 | 1.5 | 9 |
| JAMUI | BIHAR | 31.78 | 9 | 2.87 | 9 | 45.4 | 6 | 9.38 | 7 | 1.26 | 6 | 0.6 | 7 |
| JEHANABAD | BIHAR | 28.38 | 6 | 1.94 | 6 | 37.8 | 3 | 7.80 | 3 | 0.69 | 3 | 0.2 | 3 |
| ARWAL | BIHAR | 34.43 | 10 | 2.82 | 10 | 33.8 | 2 | 7.10 | 2 | 0.55 | 2 | 0.1 | 2 |
| NORTH DISTRICT | SIKKIM | 23.81 | 2 | 1.35 | 2 | 15.2 | 1 | 3.40 | 1 | 0.28 | 1 | 0.0 | 1 |
| WEST DISTRICT | SIKKIM | 27.93 | 6 | 1.49 | 6 | 23.1 | 1 | 6.51 | 1 | 0.29 | 1 | 0.0 | 1 |
| SOUTH DISTRICT | SIKKIM | 25.35 | 3 | 1.25 | 3 | 21.2 | 1 | 5.58 | 2 | 0.35 | 1 | 0.1 | 2 |
| EAST DISTRICT | SIKKIM | 24.07 | 2 | 1.10 | 2 | 20.3 | 1 | 3.91 | 1 | 0.30 | 1 | 0.0 | 1 |
| TAWANG | ARUNACHAL PRADESH | 29.54 | 7 | 2.12 | 7 | 22.1 | 3 | 6.38 | 5 | 0.57 | 3 | 0.3 | 5 |
| WEST KAMENG | ARUNACHAL PRADESH | 25.09 | 3 | 1.60 | 3 | 22.1 | 1 | 6.08 | 1 | 0.35 | 1 | 0.0 | 1 |
| EAST KAMENG | ARUNACHAL PRADESH | 26.96 | 5 | 2.52 | 5 | 18.9 | 3 | 7.86 | 7 | 0.66 | 3 | 0.6 | 7 |
| PAPUM PARE | ARUNACHAL PRADESH | 27.41 | 5 | 1.73 | 5 | 16.8 | 3 | 5.20 | 6 | 0.60 | 3 | 0.4 | 6 |
| UPPER SUBANSIRI | ARUNACHAL PRADESH | 24.98 | 3 | 2.50 | 3 | 22.4 | 2 | 7.75 | 3 | 0.54 | 2 | 0.2 | 3 |
| UPPER SIANG | ARUNACHAL PRADESH | 29.39 | 7 | 2.21 | 7 | 15.5 | 1 | 2.92 | 2 | 0.40 | 1 | 0.1 | 2 |
| CHANGLANG | ARUNACHAL PRADESH | 35.05 | 10 | 2.90 | 10 | 16.2 | 1 | 3.97 | 1 | 0.32 | 1 | 0.0 | 1 |
| LOWER SUBANSIRI | ARUNACHAL PRADESH | 26.74 | 5 | 1.80 | 5 | 20.7 | 2 | 5.03 | 2 | 0.47 | 2 | 0.1 | 2 |
| DIBANG VALLEY | ARUNACHAL PRADESH | 27.70 | 6 | 1.65 | 6 | 19.8 | 1 | 5.64 | 1 | 0.35 | 1 | 0.0 | 1 |
| LOWER DIBANG VALLEY | ARUNACHAL PRADESH | 27.74 | 6 | 1.81 | 6 | 16.9 | 2 | 4.55 | 2 | 0.45 | 2 | 0.1 | 2 |
| ANJAW | ARUNACHAL PRADESH | 23.66 | 2 | 1.34 | 2 | 17.5 | 1 | 4.65 | 2 | 0.40 | 1 | 0.1 | 2 |
| MON | NAGALAND | 21.11 | 1 | 1.24 | 1 | 9.3 | 1 | 2.62 | 1 | 0.30 | 1 | 0.0 | 1 |
| MOKOKCHUNG | NAGALAND | 21.14 | 1 | 1.52 | 1 | 10.1 | 1 | 3.52 | 1 | 0.31 | 1 | 0.0 | 1 |
| ZUNHEBOTO | NAGALAND | 17.66 | 1 | 1.10 | 1 | 7.8 | 1 | 2.15 | 2 | 0.35 | 1 | 0.1 | 2 |
| WOKHA | NAGALAND | 24.31 | 2 | 1.36 | 2 | 9.2 | 1 | 1.99 | 2 | 0.39 | 1 | 0.1 | 2 |
| DIMAPUR | NAGALAND | 25.32 | 3 | 1.96 | 3 | 12.3 | 1 | 3.67 | 2 | 0.36 | 1 | 0.1 | 2 |
| PHEK | NAGALAND | 22.06 | 1 | 1.72 | 1 | 8.8 | 1 | 3.64 | 2 | 0.34 | 1 | 0.1 | 2 |
| TUENSANG | NAGALAND | 17.97 | 1 | 1.37 | 1 | 7.2 | 1 | 1.84 | 2 | 0.31 | 1 | 0.1 | 2 |
| LONGLENG | NAGALAND | 25.75 | 3 | 2.42 | 3 | 9.3 | 2 | 3.05 | 4 | 0.43 | 2 | 0.2 | 4 |
| KIPHIRE | NAGALAND | 22.31 | 1 | 1.93 | 1 | 13.8 | 1 | 6.28 | 2 | 0.40 | 1 | 0.1 | 2 |
| KOHIMA | NAGALAND | 21.46 | 1 | 1.54 | 1 | 6.0 | 1 | 1.12 | 2 | 0.37 | 1 | 0.1 | 2 |
| PEREN | NAGALAND | 23.58 | 2 | 2.19 | 2 | 12.8 | 2 | 3.77 | 4 | 0.48 | 2 | 0.3 | 4 |
| SENAPATI | MANIPUR | 25.23 | 3 | 2.11 | 3 | 14.5 | 1 | 4.37 | 1 | 0.23 | 1 | 0.0 | 1 |
| TAMENGLONG | MANIPUR | 22.04 | 1 | 1.47 | 1 | 12.5 | 1 | 3.43 | 3 | 0.31 | 1 | 0.2 | 3 |
| CHURACHANDPUR | MANIPUR | 21.23 | 1 | 1.80 | 1 | 15.4 | 1 | 3.97 | 2 | 0.32 | 1 | 0.1 | 2 |
| BISHNUPUR | MANIPUR | 22.28 | 1 | 1.69 | 1 | 16.6 | 1 | 4.44 | 3 | 0.39 | 1 | 0.1 | 3 |
| THOUBAL | MANIPUR | 23.41 | 1 | 1.79 | 1 | 14.0 | 1 | 3.66 | 4 | 0.36 | 1 | 0.2 | 4 |
| IMPHAL WEST | MANIPUR | 25.88 | 4 | 2.10 | 4 | 11.9 | 1 | 2.88 | 1 | 0.24 | 1 | 0.0 | 1 |
| IMPHAL EAST | MANIPUR | 26.02 | 4 | 1.92 | 4 | 18.7 | 1 | 5.26 | 1 | 0.24 | 1 | 0.0 | 1 |
| UKHRUL | MANIPUR | 18.17 | 1 | 1.11 | 1 | 9.9 | 1 | 1.99 | 2 | 0.32 | 1 | 0.1 | 2 |
| CHANDEL | MANIPUR | 21.34 | 1 | 1.60 | 1 | 15.9 | 1 | 4.75 | 1 | 0.24 | 1 | 0.0 | 1 |
| MAMIT | MIZORAM | 27.26 | 5 | 1.90 | 5 | 18.4 | 1 | 6.16 | 2 | 0.27 | 1 | 0.1 | 2 |
| KOLASIB | MIZORAM | 28.38 | 6 | 2.02 | 6 | 18.3 | 1 | 5.16 | 1 | 0.26 | 1 | 0.1 | 1 |
| AIZAWL | MIZORAM | 22.77 | 1 | 1.60 | 1 | 21.1 | 1 | 5.90 | 1 | 0.24 | 1 | 0.0 | 1 |
| CHAMPHAI | MIZORAM | 23.31 | 1 | 1.69 | 1 | 10.6 | 1 | 2.83 | 2 | 0.27 | 1 | 0.1 | 2 |
| SERCHHIP | MIZORAM | 25.01 | 3 | 1.60 | 3 | 15.9 | 1 | 4.30 | 1 | 0.26 | 1 | 0.1 | 1 |
| LUNGLEI | MIZORAM | 26.22 | 4 | 1.43 | 4 | 16.4 | 1 | 4.85 | 1 | 0.24 | 1 | 0.0 | 1 |
| LAWNGTLAI | MIZORAM | 26.97 | 5 | 2.15 | 5 | 24.1 | 1 | 7.44 | 1 | 0.25 | 1 | 0.1 | 1 |
| SAIHA | MIZORAM | 23.77 | 2 | 1.91 | 2 | 19.2 | 1 | 4.06 | 2 | 0.30 | 1 | 0.1 | 2 |
| DHALAI | TRIPURA | 38.05 | 10 | 2.60 | 10 | 35.9 | 1 | 8.06 | 2 | 0.39 | 1 | 0.1 | 2 |
| SOUTH GARO HILLS | MEGHALAYA | 25.35 | 3 | 2.47 | 3 | 6.5 | 1 | 1.45 | 1 | 0.23 | 1 | 0.0 | 1 |
| RIBHOI | MEGHALAYA | 22.01 | 1 | 2.18 | 1 | 17.7 | 2 | 8.66 | 5 | 0.47 | 2 | 0.3 | 5 |
| EAST KHASI HILLS | MEGHALAYA | 18.59 | 1 | 1.60 | 1 | 11.2 | 2 | 3.51 | 3 | 0.42 | 2 | 0.1 | 3 |
| KOKRAJHAR | ASSAM | 43.58 | 10 | 3.13 | 10 | 28.0 | 2 | 7.32 | 3 | 0.50 | 2 | 0.1 | 3 |
| GOALPARA | ASSAM | 33.26 | 9 | 3.14 | 9 | 30.7 | 2 | 9.18 | 3 | 0.48 | 2 | 0.1 | 3 |
| BARPETA | ASSAM | 36.45 | 10 | 2.95 | 10 | 28.4 | 1 | 8.11 | 2 | 0.41 | 1 | 0.1 | 2 |
| MORIGAON | ASSAM | 32.22 | 9 | 1.91 | 9 | 26.9 | 1 | 9.23 | 1 | 0.35 | 1 | 0.0 | 1 |
| LAKHIMPUR | ASSAM | 36.07 | 10 | 2.72 | 10 | 35.3 | 2 | 9.90 | 2 | 0.43 | 2 | 0.1 | 2 |
| DHEMAJI | ASSAM | 35.88 | 10 | 2.43 | 10 | 33.9 | 3 | 7.46 | 4 | 0.67 | 3 | 0.2 | 4 |
| TINSUKIA | ASSAM | 36.09 | 10 | 2.35 | 10 | 29.8 | 3 | 7.35 | 3 | 0.59 | 3 | 0.2 | 3 |
| DIBRUGARH | ASSAM | 37.22 | 10 | 2.51 | 10 | 27.8 | 3 | 7.06 | 4 | 0.67 | 3 | 0.2 | 4 |
| GOLAGHAT | ASSAM | 31.29 | 9 | 2.00 | 9 | 46.2 | 2 | 7.92 | 2 | 0.47 | 2 | 0.1 | 2 |
| DIMA HASAO | ASSAM | 33.17 | 9 | 2.02 | 9 | 38.6 | 3 | 9.81 | 5 | 0.71 | 3 | 0.3 | 5 |
| CACHAR | ASSAM | 31.53 | 9 | 2.42 | 9 | 32.2 | 1 | 8.76 | 1 | 0.35 | 1 | 0.0 | 1 |
| KARIMGANJ | ASSAM | 34.60 | 10 | 2.50 | 10 | 27.8 | 1 | 9.22 | 1 | 0.33 | 1 | 0.0 | 1 |
| HAILAKANDI | ASSAM | 31.91 | 9 | 2.40 | 9 | 27.8 | 3 | 8.21 | 5 | 0.66 | 3 | 0.3 | 5 |
| BONGAIGAON | ASSAM | 36.60 | 10 | 2.31 | 10 | 36.9 | 2 | 8.65 | 3 | 0.49 | 2 | 0.1 | 3 |
| CHIRANG | ASSAM | 29.04 | 7 | 1.97 | 7 | 46.8 | 2 | 12.40 | 3 | 0.56 | 2 | 0.2 | 3 |
| KAMRUP | ASSAM | 38.16 | 10 | 2.01 | 10 | 27.8 | 3 | 6.04 | 4 | 0.61 | 3 | 0.2 | 4 |
| KAMRUP METROPOLITAN | ASSAM | 36.62 | 10 | 2.21 | 10 | 37.6 | 4 | 8.10 | 6 | 0.86 | 4 | 0.4 | 6 |
| NALBARI | ASSAM | 37.03 | 10 | 2.44 | 10 | 29.3 | 3 | 7.01 | 5 | 0.60 | 3 | 0.3 | 5 |
| BAKSA | ASSAM | 33.61 | 9 | 2.60 | 9 | 43.2 | 2 | 10.56 | 3 | 0.54 | 2 | 0.2 | 3 |
| DARRANG | ASSAM | 39.86 | 10 | 2.62 | 10 | 24.4 | 3 | 6.83 | 3 | 0.57 | 3 | 0.2 | 3 |
| UDALGURI | ASSAM | 35.41 | 10 | 2.09 | 10 | 39.3 | 1 | 8.25 | 1 | 0.38 | 1 | 0.0 | 1 |
| DARJILING | WEST BENGAL | 35.93 | 10 | 2.37 | 10 | 25.7 | 3 | 7.61 | 3 | 0.61 | 3 | 0.2 | 3 |
| JALPAIGURI | WEST BENGAL | 34.77 | 10 | 2.46 | 10 | 29.4 | 2 | 7.20 | 1 | 0.43 | 2 | 0.0 | 1 |
| KOCH BIHAR | WEST BENGAL | 33.85 | 10 | 1.99 | 10 | 29.5 | 2 | 6.69 | 3 | 0.53 | 2 | 0.1 | 3 |
| UTTAR DINAJPUR | WEST BENGAL | 35.73 | 10 | 2.78 | 10 | 37.1 | 3 | 9.34 | 3 | 0.57 | 3 | 0.2 | 3 |
| DAKSHIN DINAJPUR | WEST BENGAL | 34.49 | 10 | 2.04 | 10 | 35.9 | 3 | 7.21 | 4 | 0.72 | 3 | 0.2 | 4 |
| MALDAH | WEST BENGAL | 30.68 | 8 | 2.53 | 8 | 38.7 | 5 | 9.05 | 6 | 0.96 | 5 | 0.4 | 6 |
| MURSHIDABAD | WEST BENGAL | 34.40 | 10 | 2.56 | 10 | 34.4 | 2 | 8.35 | 3 | 0.52 | 2 | 0.2 | 3 |
| BIRBHUM | WEST BENGAL | 34.65 | 10 | 2.75 | 10 | 37.6 | 3 | 9.83 | 3 | 0.59 | 3 | 0.2 | 3 |
| NADIA | WEST BENGAL | 30.18 | 8 | 1.87 | 8 | 34.3 | 3 | 8.94 | 3 | 0.61 | 3 | 0.2 | 3 |
| NORTH TWENTY FOUR PARGANAS | WEST BENGAL | 36.76 | 10 | 2.93 | 10 | 18.4 | 2 | 3.73 | 2 | 0.51 | 2 | 0.1 | 2 |
| HUGLI | WEST BENGAL | 32.61 | 9 | 2.12 | 9 | 34.0 | 4 | 6.65 | 7 | 0.87 | 4 | 0.6 | 7 |
| BANKURA | WEST BENGAL | 31.50 | 9 | 1.86 | 9 | 35.5 | 2 | 8.06 | 2 | 0.53 | 2 | 0.1 | 2 |
| PURULIYA | WEST BENGAL | 32.66 | 9 | 2.15 | 9 | 41.4 | 3 | 8.86 | 4 | 0.73 | 3 | 0.3 | 4 |
| HAORA | WEST BENGAL | 34.65 | 10 | 1.94 | 10 | 30.7 | 2 | 7.32 | 2 | 0.51 | 2 | 0.1 | 2 |
| KOLKATA | WEST BENGAL | 39.26 | 10 | 1.65 | 10 | 26.6 | 2 | 4.35 | 1 | 0.49 | 2 | 0.0 | 1 |
| SOUTH TWENTY FOUR PARGANAS | WEST BENGAL | 36.68 | 10 | 2.51 | 10 | 28.9 | 1 | 6.45 | 1 | 0.41 | 1 | 0.0 | 1 |
| PASCHIM MEDINIPUR | WEST BENGAL | 32.03 | 9 | 1.98 | 9 | 35.7 | 2 | 8.49 | 1 | 0.44 | 2 | 0.0 | 1 |
| PURBA MEDINIPUR | WEST BENGAL | 32.34 | 9 | 1.91 | 9 | 31.1 | 3 | 6.12 | 3 | 0.61 | 3 | 0.1 | 3 |
| GARHWA | JHARKHAND | 30.90 | 8 | 2.18 | 8 | 30.7 | 3 | 6.91 | 4 | 0.71 | 3 | 0.2 | 4 |
| CHATRA | JHARKHAND | 32.33 | 9 | 2.73 | 9 | 29.0 | 2 | 7.65 | 3 | 0.56 | 2 | 0.2 | 3 |
| KODARMA | JHARKHAND | 34.14 | 10 | 2.36 | 10 | 24.5 | 2 | 6.87 | 2 | 0.48 | 2 | 0.1 | 2 |
| GIRIDIH | JHARKHAND | 27.57 | 6 | 2.07 | 6 | 39.2 | 2 | 9.93 | 2 | 0.42 | 2 | 0.1 | 2 |
| DEOGHAR | JHARKHAND | 28.75 | 7 | 2.08 | 7 | 43.8 | 2 | 10.36 | 3 | 0.54 | 2 | 0.2 | 3 |
| GODDA | JHARKHAND | 34.39 | 10 | 2.79 | 10 | 36.0 | 4 | 8.81 | 5 | 0.78 | 4 | 0.3 | 5 |
| SAHIBGANJ | JHARKHAND | 33.59 | 9 | 2.80 | 9 | 36.2 | 4 | 9.30 | 5 | 0.76 | 4 | 0.3 | 5 |
| PAKUR | JHARKHAND | 35.14 | 10 | 1.62 | 10 | 35.0 | 5 | 6.74 | 5 | 0.91 | 5 | 0.4 | 5 |
| DHANBAD | JHARKHAND | 27.57 | 6 | 1.79 | 6 | 37.5 | 6 | 7.78 | 8 | 1.16 | 6 | 0.9 | 8 |
| BOKARO | JHARKHAND | 30.09 | 8 | 1.93 | 8 | 33.6 | 3 | 6.59 | 3 | 0.61 | 3 | 0.2 | 3 |
| LOHARDAGA | JHARKHAND | 33.54 | 9 | 2.54 | 9 | 33.7 | 1 | 9.45 | 1 | 0.39 | 1 | 0.0 | 1 |
| PURBI SINGHBHUM | JHARKHAND | 31.70 | 9 | 2.18 | 9 | 35.2 | 1 | 8.10 | 1 | 0.41 | 1 | 0.0 | 1 |
| PALAMU | JHARKHAND | 32.16 | 9 | 2.20 | 9 | 34.7 | 4 | 8.54 | 5 | 0.80 | 4 | 0.3 | 5 |
| LATEHAR | JHARKHAND | 32.38 | 9 | 2.12 | 9 | 36.1 | 2 | 8.82 | 2 | 0.42 | 2 | 0.1 | 2 |
| HAZARIBAGH | JHARKHAND | 30.01 | 8 | 2.74 | 8 | 30.8 | 2 | 8.59 | 3 | 0.55 | 2 | 0.2 | 3 |
| RAMGARH | JHARKHAND | 31.85 | 9 | 2.24 | 9 | 28.9 | 1 | 7.95 | 1 | 0.41 | 1 | 0.0 | 1 |
| DUMKA | JHARKHAND | 34.13 | 10 | 2.09 | 10 | 38.1 | 3 | 8.20 | 5 | 0.65 | 3 | 0.3 | 5 |
| JAMTARA | JHARKHAND | 28.65 | 7 | 1.98 | 7 | 43.0 | 3 | 9.95 | 6 | 0.64 | 3 | 0.4 | 6 |
| RANCHI | JHARKHAND | 33.70 | 10 | 2.36 | 10 | 27.0 | 4 | 7.70 | 5 | 0.81 | 4 | 0.3 | 5 |
| KHUNTI | JHARKHAND | 28.81 | 7 | 2.48 | 7 | 38.5 | 1 | 9.84 | 1 | 0.41 | 1 | 0.0 | 1 |
| GUMLA | JHARKHAND | 34.24 | 10 | 2.16 | 10 | 30.2 | 2 | 8.10 | 3 | 0.52 | 2 | 0.1 | 3 |
| SIMDEGA | JHARKHAND | 30.41 | 8 | 1.91 | 8 | 43.2 | 3 | 9.45 | 3 | 0.59 | 3 | 0.2 | 3 |
| PASHCHIMI SINGHBHUM | JHARKHAND | 31.41 | 9 | 2.07 | 9 | 41.5 | 2 | 9.41 | 3 | 0.54 | 2 | 0.2 | 3 |
| SARAIKELA-KHARSAWAN | JHARKHAND | 33.29 | 9 | 1.90 | 9 | 37.6 | 3 | 7.87 | 5 | 0.62 | 3 | 0.3 | 5 |
| BARGARH | ODISHA | 27.05 | 5 | 1.51 | 5 | 33.3 | 3 | 7.12 | 3 | 0.57 | 3 | 0.2 | 3 |
| JHARSUGUDA | ODISHA | 33.94 | 10 | 1.89 | 10 | 27.3 | 2 | 5.71 | 2 | 0.51 | 2 | 0.1 | 2 |
| SAMBALPUR | ODISHA | 32.20 | 9 | 1.89 | 9 | 29.3 | 2 | 6.27 | 1 | 0.43 | 2 | 0.0 | 1 |
| DEBAGARH | ODISHA | 31.00 | 8 | 1.76 | 8 | 29.1 | 4 | 6.34 | 5 | 0.76 | 4 | 0.3 | 5 |
| SUNDARGARH | ODISHA | 29.26 | 7 | 1.68 | 7 | 42.6 | 4 | 6.26 | 5 | 0.83 | 4 | 0.3 | 5 |
| KENDUJHAR | ODISHA | 31.27 | 8 | 1.80 | 8 | 33.8 | 2 | 6.67 | 2 | 0.52 | 2 | 0.1 | 2 |
| MAYURBHANJ | ODISHA | 28.39 | 6 | 1.52 | 6 | 40.4 | 3 | 8.24 | 4 | 0.64 | 3 | 0.2 | 4 |
| BALESHWAR | ODISHA | 25.69 | 3 | 1.67 | 3 | 23.7 | 3 | 5.36 | 4 | 0.67 | 3 | 0.2 | 4 |
| BHADRAK | ODISHA | 32.50 | 9 | 2.10 | 9 | 32.3 | 2 | 7.91 | 2 | 0.49 | 2 | 0.1 | 2 |
| KENDRAPARA | ODISHA | 34.14 | 10 | 1.67 | 10 | 22.4 | 3 | 5.50 | 3 | 0.60 | 3 | 0.2 | 3 |
| JAGATSINGHAPUR | ODISHA | 30.29 | 8 | 1.66 | 8 | 25.8 | 3 | 5.43 | 4 | 0.72 | 3 | 0.2 | 4 |
| CUTTACK | ODISHA | 31.06 | 8 | 1.79 | 8 | 29.6 | 3 | 5.35 | 4 | 0.70 | 3 | 0.2 | 4 |
| JAJAPUR | ODISHA | 28.53 | 6 | 1.90 | 6 | 34.5 | 5 | 7.86 | 5 | 0.91 | 5 | 0.4 | 5 |
| DHENKANAL | ODISHA | 29.36 | 7 | 1.70 | 7 | 32.0 | 3 | 6.17 | 4 | 0.68 | 3 | 0.2 | 4 |
| ANUGUL | ODISHA | 28.05 | 6 | 1.75 | 6 | 44.3 | 2 | 8.73 | 2 | 0.48 | 2 | 0.1 | 2 |
| NAYAGARH | ODISHA | 34.34 | 10 | 2.34 | 10 | 19.9 | 1 | 5.13 | 1 | 0.41 | 1 | 0.0 | 1 |
| KHORDHA | ODISHA | 33.19 | 9 | 2.03 | 9 | 22.0 | 2 | 4.61 | 2 | 0.52 | 2 | 0.1 | 2 |
| PURI | ODISHA | 31.36 | 9 | 1.66 | 9 | 20.0 | 2 | 4.03 | 1 | 0.43 | 2 | 0.0 | 1 |
| GANJAM | ODISHA | 33.86 | 10 | 2.39 | 10 | 26.2 | 1 | 5.04 | 1 | 0.41 | 1 | 0.0 | 1 |
| GAJAPATI | ODISHA | 34.24 | 10 | 2.53 | 10 | 27.0 | 2 | 5.77 | 2 | 0.47 | 2 | 0.1 | 2 |
| KANDHAMAL | ODISHA | 31.92 | 9 | 2.90 | 9 | 23.8 | 2 | 6.05 | 2 | 0.43 | 2 | 0.1 | 2 |
| BAUDH | ODISHA | 31.35 | 9 | 1.59 | 9 | 24.3 | 2 | 5.21 | 2 | 0.47 | 2 | 0.1 | 2 |
| SUBARNAPUR | ODISHA | 40.56 | 10 | 2.52 | 10 | 24.9 | 1 | 6.49 | 1 | 0.41 | 1 | 0.0 | 1 |
| BALANGIR | ODISHA | 33.99 | 10 | 2.13 | 10 | 33.5 | 3 | 7.92 | 4 | 0.64 | 3 | 0.2 | 4 |
| NUAPADA | ODISHA | 31.71 | 9 | 2.14 | 9 | 38.0 | 2 | 7.24 | 3 | 0.54 | 2 | 0.1 | 3 |
| KALAHANDI | ODISHA | 32.68 | 9 | 1.90 | 9 | 33.5 | 4 | 7.72 | 8 | 0.74 | 4 | 0.8 | 8 |
| RAYAGADA | ODISHA | 30.86 | 8 | 2.22 | 8 | 36.0 | 2 | 8.86 | 5 | 0.56 | 2 | 0.3 | 5 |
| NABARANGAPUR | ODISHA | 36.49 | 10 | 3.26 | 10 | 32.6 | 3 | 8.11 | 4 | 0.61 | 3 | 0.2 | 4 |
| KORAPUT | ODISHA | 32.34 | 9 | 1.96 | 9 | 30.7 | 2 | 6.74 | 2 | 0.47 | 2 | 0.1 | 2 |
| MALKANGIRI | ODISHA | 35.20 | 10 | 3.11 | 10 | 36.1 | 4 | 9.89 | 6 | 0.80 | 4 | 0.5 | 6 |
| KORIYA | CHHATTISGARH | 27.69 | 6 | 1.95 | 6 | 28.7 | 4 | 7.46 | 4 | 0.89 | 4 | 0.3 | 4 |
| JASHPUR | CHHATTISGARH | 28.33 | 6 | 1.99 | 6 | 25.2 | 4 | 6.64 | 4 | 0.78 | 4 | 0.2 | 4 |
| RAIGARH | CHHATTISGARH | 26.29 | 4 | 1.34 | 4 | 36.8 | 4 | 7.49 | 3 | 0.74 | 4 | 0.2 | 3 |
| KORBA | CHHATTISGARH | 24.95 | 3 | 1.29 | 3 | 36.8 | 7 | 7.88 | 8 | 1.47 | 7 | 0.8 | 8 |
| JANJGIR - CHAMPA | CHHATTISGARH | 26.52 | 4 | 1.70 | 4 | 42.9 | 8 | 8.13 | 7 | 1.57 | 8 | 0.7 | 7 |
| KABEERDHAM | CHHATTISGARH | 29.17 | 7 | 2.26 | 7 | 29.2 | 4 | 8.63 | 3 | 0.77 | 4 | 0.2 | 3 |
| RAJNANDGAON | CHHATTISGARH | 34.70 | 10 | 1.61 | 10 | 37.8 | 3 | 5.34 | 3 | 0.70 | 3 | 0.1 | 3 |
| MAHASAMUND | CHHATTISGARH | 28.73 | 7 | 1.98 | 7 | 42.0 | 3 | 8.67 | 3 | 0.73 | 3 | 0.2 | 3 |
| DHAMTARI | CHHATTISGARH | 28.85 | 7 | 1.71 | 7 | 35.6 | 5 | 7.39 | 5 | 1.06 | 5 | 0.4 | 5 |
| UTTAR BASTAR KANKER | CHHATTISGARH | 28.74 | 7 | 1.77 | 7 | 34.1 | 5 | 7.97 | 5 | 0.99 | 5 | 0.3 | 5 |
| NARAYANPUR | CHHATTISGARH | 29.23 | 7 | 2.25 | 7 | 50.8 | 4 | 10.25 | 4 | 0.76 | 4 | 0.2 | 4 |
| BIJAPUR | CHHATTISGARH | 27.02 | 5 | 1.75 | 5 | 46.1 | 4 | 9.50 | 3 | 0.74 | 4 | 0.2 | 3 |
| SHEOPUR | MADHYA PRADESH | 26.86 | 5 | 1.90 | 5 | 41.5 | 9 | 9.87 | 9 | 2.14 | 9 | 1.2 | 9 |
| MORENA | MADHYA PRADESH | 25.54 | 3 | 2.59 | 3 | 45.1 | 8 | 8.04 | 8 | 1.66 | 8 | 0.8 | 8 |
| BHIND | MADHYA PRADESH | 27.69 | 6 | 1.70 | 6 | 40.7 | 10 | 8.19 | 9 | 2.51 | 10 | 1.0 | 9 |
| GWALIOR | MADHYA PRADESH | 27.11 | 5 | 1.63 | 5 | 42.3 | 10 | 7.75 | 10 | 3.38 | 10 | 1.9 | 10 |
| DATIA | MADHYA PRADESH | 29.63 | 7 | 1.81 | 7 | 39.3 | 8 | 5.71 | 8 | 1.56 | 8 | 0.8 | 8 |
| SHIVPURI | MADHYA PRADESH | 26.48 | 4 | 1.60 | 4 | 42.1 | 7 | 7.65 | 6 | 1.45 | 7 | 0.4 | 6 |
| TIKAMGARH | MADHYA PRADESH | 29.68 | 8 | 1.74 | 8 | 34.7 | 6 | 5.36 | 1 | 1.21 | 6 | 0.1 | 1 |
| CHHATARPUR | MADHYA PRADESH | 23.05 | 1 | 1.47 | 1 | 57.7 | 10 | 7.39 | 10 | 2.88 | 10 | 2.3 | 10 |
| PANNA | MADHYA PRADESH | 25.99 | 4 | 1.48 | 4 | 45.6 | 8 | 7.54 | 9 | 1.75 | 8 | 1.5 | 9 |
| SAGAR | MADHYA PRADESH | 23.34 | 1 | 1.68 | 1 | 56.2 | 9 | 7.95 | 10 | 2.24 | 9 | 2.4 | 10 |
| DAMOH | MADHYA PRADESH | 26.51 | 4 | 1.63 | 4 | 45.4 | 9 | 8.65 | 10 | 2.32 | 9 | 2.8 | 10 |
| SATNA | MADHYA PRADESH | 26.47 | 4 | 1.55 | 4 | 50.9 | 8 | 7.76 | 7 | 1.71 | 8 | 0.6 | 7 |
| REWA | MADHYA PRADESH | 31.56 | 9 | 2.24 | 9 | 39.5 | 8 | 9.40 | 6 | 1.56 | 8 | 0.5 | 6 |
| UMARIA | MADHYA PRADESH | 28.48 | 6 | 2.12 | 6 | 40.5 | 7 | 7.61 | 6 | 1.34 | 7 | 0.4 | 6 |
| NEEMUCH | MADHYA PRADESH | 29.92 | 8 | 2.08 | 8 | 41.5 | 8 | 10.03 | 9 | 1.56 | 8 | 1.3 | 9 |
| MANDSAUR | MADHYA PRADESH | 27.08 | 5 | 1.60 | 5 | 34.0 | 9 | 7.92 | 9 | 2.32 | 9 | 1.1 | 9 |
| RATLAM | MADHYA PRADESH | 26.73 | 5 | 1.70 | 5 | 42.5 | 10 | 8.02 | 10 | 2.77 | 10 | 1.6 | 10 |
| UJJAIN | MADHYA PRADESH | 26.05 | 4 | 2.00 | 4 | 52.4 | 9 | 9.14 | 10 | 2.29 | 9 | 1.7 | 10 |
| DEWAS | MADHYA PRADESH | 25.76 | 3 | 2.01 | 3 | 46.9 | 10 | 9.06 | 10 | 3.38 | 10 | 1.6 | 10 |
| DHAR | MADHYA PRADESH | 27.02 | 5 | 1.99 | 5 | 38.4 | 10 | 7.85 | 10 | 2.78 | 10 | 4.1 | 10 |
| INDORE | MADHYA PRADESH | 28.20 | 6 | 1.81 | 6 | 44.6 | 9 | 7.44 | 8 | 2.06 | 9 | 0.9 | 8 |
| KHARGONE (WEST NIMAR) | MADHYA PRADESH | 30.27 | 8 | 2.20 | 8 | 36.4 | 10 | 9.44 | 10 | 2.47 | 10 | 1.9 | 10 |
| BARWANI | MADHYA PRADESH | 30.17 | 8 | 2.91 | 8 | 40.8 | 10 | 10.45 | 10 | 4.09 | 10 | 8.0 | 10 |
| RAJGARH | MADHYA PRADESH | 24.17 | 2 | 1.72 | 2 | 47.3 | 9 | 7.88 | 10 | 2.46 | 9 | 1.8 | 10 |
| VIDISHA | MADHYA PRADESH | 27.14 | 5 | 1.65 | 5 | 25.6 | 8 | 6.98 | 7 | 1.54 | 8 | 0.6 | 7 |
| BHOPAL | MADHYA PRADESH | 29.88 | 8 | 2.30 | 8 | 34.8 | 8 | 6.98 | 5 | 1.58 | 8 | 0.4 | 5 |
| SEHORE | MADHYA PRADESH | 23.32 | 1 | 1.26 | 1 | 53.0 | 9 | 7.80 | 9 | 2.40 | 9 | 1.1 | 9 |
| RAISEN | MADHYA PRADESH | 28.65 | 7 | 1.80 | 7 | 30.6 | 6 | 5.37 | 2 | 1.29 | 6 | 0.1 | 2 |
| BETUL | MADHYA PRADESH | 27.21 | 5 | 1.79 | 5 | 34.1 | 5 | 8.61 | 3 | 0.99 | 5 | 0.2 | 3 |
| HARDA | MADHYA PRADESH | 21.49 | 1 | 1.38 | 1 | 60.1 | 10 | 7.34 | 10 | 2.51 | 10 | 1.7 | 10 |
| HOSHANGABAD | MADHYA PRADESH | 25.91 | 4 | 1.53 | 4 | 46.5 | 10 | 6.90 | 10 | 2.61 | 10 | 1.7 | 10 |
| KATNI | MADHYA PRADESH | 31.57 | 9 | 1.88 | 9 | 41.7 | 6 | 7.81 | 6 | 1.31 | 6 | 0.4 | 6 |
| JABALPUR | MADHYA PRADESH | 26.51 | 4 | 0.91 | 4 | 28.2 | 7 | 5.22 | 2 | 1.45 | 7 | 0.1 | 2 |
| NARSIMHAPUR | MADHYA PRADESH | 24.90 | 3 | 1.51 | 3 | 44.3 | 9 | 8.66 | 9 | 1.99 | 9 | 1.5 | 9 |
| DINDORI | MADHYA PRADESH | 27.25 | 5 | 2.04 | 5 | 45.5 | 7 | 10.02 | 7 | 1.48 | 7 | 0.7 | 7 |
| MANDLA | MADHYA PRADESH | 28.29 | 6 | 1.52 | 6 | 39.0 | 5 | 7.70 | 3 | 0.99 | 5 | 0.2 | 3 |
| CHHINDWARA | MADHYA PRADESH | 25.37 | 3 | 1.45 | 3 | 28.8 | 8 | 5.75 | 6 | 1.54 | 8 | 0.5 | 6 |
| SEONI | MADHYA PRADESH | 28.18 | 6 | 1.86 | 6 | 39.6 | 6 | 8.80 | 6 | 1.29 | 6 | 0.4 | 6 |
| BALAGHAT | MADHYA PRADESH | 27.49 | 5 | 1.77 | 5 | 32.9 | 6 | 6.33 | 3 | 1.20 | 6 | 0.2 | 3 |
| GUNA | MADHYA PRADESH | 30.89 | 8 | 2.16 | 8 | 37.9 | 7 | 8.89 | 8 | 1.49 | 7 | 0.9 | 8 |
| ASHOKNAGAR | MADHYA PRADESH | 29.34 | 7 | 1.85 | 7 | 27.7 | 6 | 6.33 | 5 | 1.28 | 6 | 0.4 | 5 |
| SHAHDOL | MADHYA PRADESH | 27.31 | 5 | 1.30 | 5 | 29.1 | 6 | 5.91 | 5 | 1.21 | 6 | 0.3 | 5 |
| ANUPPUR | MADHYA PRADESH | 26.32 | 4 | 1.40 | 4 | 24.7 | 5 | 5.06 | 1 | 0.92 | 5 | 0.0 | 1 |
| SIDHI | MADHYA PRADESH | 31.67 | 9 | 2.66 | 9 | 37.1 | 7 | 9.12 | 8 | 1.42 | 7 | 0.7 | 8 |
| SINGRAULI | MADHYA PRADESH | 30.34 | 8 | 1.70 | 8 | 24.8 | 5 | 5.16 | 1 | 1.08 | 5 | 0.0 | 1 |
| JHABUA | MADHYA PRADESH | 30.64 | 8 | 2.28 | 8 | 45.0 | 7 | 9.61 | 9 | 1.53 | 7 | 1.5 | 9 |
| ALIRAJPUR | MADHYA PRADESH | 28.32 | 6 | 2.66 | 6 | 44.9 | 6 | 12.90 | 6 | 1.10 | 6 | 0.5 | 6 |
| KHANDWA (EAST NIMAR) | MADHYA PRADESH | 26.50 | 4 | 1.45 | 4 | 49.6 | 9 | 7.95 | 10 | 2.16 | 9 | 2.2 | 10 |
| BURHANPUR | MADHYA PRADESH | 25.43 | 3 | 1.91 | 3 | 49.8 | 8 | 9.93 | 8 | 1.59 | 8 | 0.8 | 8 |
| KACHCHH | GUJARAT | 29.97 | 8 | 2.34 | 8 | 37.9 | 6 | 7.70 | 6 | 1.26 | 6 | 0.6 | 6 |
| BANAS KANTHA | GUJARAT | 29.19 | 7 | 2.22 | 7 | 48.5 | 8 | 9.05 | 9 | 1.74 | 8 | 1.5 | 9 |
| PATAN | GUJARAT | 25.88 | 4 | 1.80 | 4 | 48.7 | 8 | 8.68 | 7 | 1.57 | 8 | 0.6 | 7 |
| MAHESANA | GUJARAT | 21.96 | 1 | 1.21 | 1 | 58.6 | 10 | 5.77 | 10 | 3.74 | 10 | 2.8 | 10 |
| GANDHINAGAR | GUJARAT | 25.71 | 3 | 1.64 | 3 | 46.4 | 10 | 7.57 | 10 | 4.65 | 10 | 2.9 | 10 |
| PORBANDAR | GUJARAT | 31.34 | 9 | 1.67 | 9 | 41.0 | 7 | 7.02 | 7 | 1.48 | 7 | 0.7 | 7 |
| AMRELI | GUJARAT | 28.74 | 7 | 1.83 | 7 | 41.2 | 5 | 8.48 | 3 | 1.06 | 5 | 0.2 | 3 |
| ANAND | GUJARAT | 28.87 | 7 | 1.62 | 7 | 45.4 | 9 | 8.17 | 8 | 1.94 | 9 | 1.0 | 8 |
| DOHAD | GUJARAT | 27.63 | 6 | 2.17 | 6 | 56.8 | 8 | 8.64 | 8 | 1.60 | 8 | 0.8 | 8 |
| NARMADA | GUJARAT | 30.12 | 8 | 2.27 | 8 | 54.2 | 8 | 8.85 | 9 | 1.70 | 8 | 1.1 | 9 |
| BHARUCH | GUJARAT | 23.25 | 1 | 1.23 | 1 | 56.9 | 9 | 7.67 | 9 | 2.20 | 9 | 1.1 | 9 |
| THE DANGS | GUJARAT | 30.79 | 8 | 2.53 | 8 | 50.5 | 5 | 10.68 | 3 | 0.94 | 5 | 0.1 | 3 |
| NAVSARI | GUJARAT | 32.64 | 9 | 2.42 | 9 | 37.3 | 7 | 7.69 | 6 | 1.47 | 7 | 0.4 | 6 |
| VALSAD | GUJARAT | 30.43 | 8 | 2.38 | 8 | 54.0 | 8 | 8.88 | 8 | 1.55 | 8 | 0.8 | 8 |
| SURAT | GUJARAT | 25.92 | 4 | 1.49 | 4 | 55.2 | 8 | 8.55 | 7 | 1.71 | 8 | 0.6 | 7 |
| TAPI | GUJARAT | 24.94 | 3 | 1.63 | 3 | 57.8 | 8 | 7.13 | 8 | 1.62 | 8 | 0.8 | 8 |
| DIU | DADRA & NAGAR HAVELI AND DAMAN & DIU | 32.18 | 9 | 2.14 | 9 | 27.3 | 3 | 5.52 | 3 | 0.70 | 3 | 0.1 | 3 |
| DAMAN | DADRA & NAGAR HAVELI AND DAMAN & DIU | 29.97 | 8 | 2.19 | 8 | 44.0 | 8 | 8.77 | 8 | 1.55 | 8 | 0.9 | 8 |
| DADRA & NAGAR HAVELI | DADRA & NAGAR HAVELI AND DAMAN & DIU | 31.23 | 8 | 2.06 | 8 | 42.4 | 4 | 6.50 | 4 | 0.80 | 4 | 0.2 | 4 |
| NANDURBAR | MAHARASHTRA | 28.32 | 6 | 2.00 | 6 | 45.4 | 10 | 8.58 | 10 | 2.81 | 10 | 1.8 | 10 |
| DHULE | MAHARASHTRA | 27.71 | 6 | 1.68 | 6 | 43.2 | 10 | 9.37 | 9 | 2.51 | 10 | 1.4 | 9 |
| JALGAON | MAHARASHTRA | 25.84 | 4 | 2.09 | 4 | 50.0 | 10 | 8.69 | 10 | 3.54 | 10 | 3.0 | 10 |
| BULDANA | MAHARASHTRA | 27.66 | 6 | 1.67 | 6 | 47.5 | 8 | 9.53 | 8 | 1.75 | 8 | 0.8 | 8 |
| AKOLA | MAHARASHTRA | 28.68 | 7 | 2.14 | 7 | 45.9 | 6 | 8.45 | 6 | 1.17 | 6 | 0.5 | 6 |
| WASHIM | MAHARASHTRA | 30.59 | 8 | 2.28 | 8 | 37.2 | 9 | 10.02 | 10 | 2.30 | 9 | 1.6 | 10 |
| AMRAVATI | MAHARASHTRA | 34.80 | 10 | 2.14 | 10 | 32.7 | 7 | 6.67 | 6 | 1.49 | 7 | 0.5 | 6 |
| WARDHA | MAHARASHTRA | 26.69 | 5 | 1.46 | 5 | 42.8 | 7 | 7.35 | 6 | 1.51 | 7 | 0.5 | 6 |
| NAGPUR | MAHARASHTRA | 31.62 | 9 | 2.29 | 9 | 34.8 | 7 | 9.65 | 6 | 1.40 | 7 | 0.6 | 6 |
| BHANDARA | MAHARASHTRA | 31.13 | 8 | 1.88 | 8 | 37.9 | 6 | 7.58 | 5 | 1.19 | 6 | 0.3 | 5 |
| GONDIYA | MAHARASHTRA | 30.26 | 8 | 1.57 | 8 | 43.1 | 8 | 7.40 | 7 | 1.60 | 8 | 0.6 | 7 |
| GADCHIROLI | MAHARASHTRA | 33.57 | 9 | 2.15 | 9 | 36.6 | 8 | 7.12 | 6 | 1.63 | 8 | 0.5 | 6 |
| CHANDRAPUR | MAHARASHTRA | 28.04 | 6 | 1.32 | 6 | 43.6 | 8 | 6.98 | 8 | 1.56 | 8 | 0.7 | 8 |
| YAVATMAL | MAHARASHTRA | 29.03 | 7 | 1.67 | 7 | 38.8 | 10 | 7.55 | 10 | 2.63 | 10 | 2.2 | 10 |
| NANDED | MAHARASHTRA | 27.16 | 5 | 1.60 | 5 | 44.8 | 9 | 8.04 | 8 | 2.06 | 9 | 0.9 | 8 |
| HINGOLI | MAHARASHTRA | 31.30 | 9 | 2.00 | 9 | 35.6 | 9 | 8.62 | 9 | 1.95 | 9 | 1.0 | 9 |
| PARBHANI | MAHARASHTRA | 24.94 | 3 | 1.49 | 3 | 48.2 | 9 | 8.28 | 8 | 1.99 | 9 | 0.9 | 8 |
| JALNA | MAHARASHTRA | 26.81 | 5 | 1.84 | 5 | 39.6 | 9 | 6.66 | 8 | 2.26 | 9 | 1.0 | 8 |
| AURANGABAD | MAHARASHTRA | 27.19 | 5 | 1.62 | 5 | 36.9 | 7 | 6.75 | 8 | 1.53 | 7 | 1.0 | 8 |
| NASHIK | MAHARASHTRA | 29.47 | 7 | 2.21 | 7 | 36.9 | 7 | 7.95 | 7 | 1.36 | 7 | 0.7 | 7 |
| MUMBAI SUBURBAN | MAHARASHTRA | 30.10 | 8 | 1.30 | 8 | 32.9 | 6 | 5.25 | 1 | 1.26 | 6 | 0.1 | 1 |
| MUMBAI | MAHARASHTRA | 32.40 | 9 | 2.03 | 9 | 33.9 | 7 | 7.21 | 5 | 1.35 | 7 | 0.3 | 5 |
| RAIGARH | MAHARASHTRA | 28.14 | 6 | 1.79 | 6 | 32.3 | 8 | 8.95 | 7 | 1.69 | 8 | 0.6 | 7 |
| PUNE | MAHARASHTRA | 27.75 | 6 | 1.39 | 6 | 30.5 | 5 | 6.20 | 3 | 1.05 | 5 | 0.2 | 3 |
| AHMADNAGAR | MAHARASHTRA | 30.13 | 8 | 1.70 | 8 | 28.2 | 10 | 5.65 | 10 | 2.59 | 10 | 6.2 | 10 |
| BID | MAHARASHTRA | 29.26 | 7 | 2.30 | 7 | 32.6 | 7 | 6.88 | 6 | 1.39 | 7 | 0.4 | 6 |
| LATUR | MAHARASHTRA | 33.19 | 9 | 2.50 | 9 | 24.2 | 5 | 6.28 | 5 | 1.09 | 5 | 0.3 | 5 |
| OSMANABAD | MAHARASHTRA | 28.05 | 6 | 2.19 | 6 | 38.2 | 6 | 8.93 | 5 | 1.15 | 6 | 0.3 | 5 |
| SOLAPUR | MAHARASHTRA | 26.34 | 4 | 1.84 | 4 | 43.1 | 7 | 9.21 | 8 | 1.46 | 7 | 0.8 | 8 |
| SATARA | MAHARASHTRA | 26.21 | 4 | 2.03 | 4 | 38.4 | 10 | 6.83 | 9 | 2.47 | 10 | 1.0 | 9 |
| RATNAGIRI | MAHARASHTRA | 26.60 | 5 | 1.35 | 5 | 40.8 | 6 | 8.14 | 4 | 1.22 | 6 | 0.3 | 4 |
| SINDHUDURG | MAHARASHTRA | 31.33 | 9 | 1.69 | 9 | 26.0 | 5 | 4.96 | 1 | 1.04 | 5 | 0.0 | 1 |
| KOLHAPUR | MAHARASHTRA | 27.59 | 6 | 1.86 | 6 | 34.2 | 8 | 7.50 | 9 | 1.84 | 8 | 1.0 | 9 |
| SANGLI | MAHARASHTRA | 27.29 | 5 | 1.81 | 5 | 31.5 | 9 | 6.98 | 9 | 2.07 | 9 | 1.0 | 9 |
| SRIKAKULAM | ANDHRA PRADESH | 25.49 | 3 | 1.17 | 3 | 33.0 | 5 | 5.44 | 1 | 0.91 | 5 | 0.0 | 1 |
| VIZIANAGARAM | ANDHRA PRADESH | 29.44 | 7 | 1.50 | 7 | 34.2 | 5 | 6.02 | 4 | 1.01 | 5 | 0.2 | 4 |
| VISAKHAPATNAM | ANDHRA PRADESH | 27.64 | 6 | 1.57 | 6 | 38.5 | 6 | 7.85 | 5 | 1.20 | 6 | 0.3 | 5 |
| EAST GODAVARI | ANDHRA PRADESH | 24.99 | 3 | 1.73 | 3 | 37.6 | 8 | 6.88 | 7 | 1.64 | 8 | 0.6 | 7 |
| WEST GODAVARI | ANDHRA PRADESH | 26.52 | 4 | 1.55 | 4 | 33.0 | 7 | 4.81 | 6 | 1.41 | 7 | 0.4 | 6 |
| KRISHNA | ANDHRA PRADESH | 29.29 | 7 | 1.79 | 7 | 29.7 | 6 | 6.05 | 5 | 1.19 | 6 | 0.3 | 5 |
| GUNTUR | ANDHRA PRADESH | 23.77 | 2 | 1.05 | 2 | 35.8 | 5 | 7.46 | 3 | 0.98 | 5 | 0.2 | 3 |
| PRAKASAM | ANDHRA PRADESH | 24.52 | 2 | 1.13 | 2 | 36.5 | 8 | 6.49 | 8 | 1.58 | 8 | 0.9 | 8 |
| SRI POTTI SRIRAMULU NELLORE | ANDHRA PRADESH | 26.80 | 5 | 1.89 | 5 | 33.7 | 9 | 7.74 | 9 | 2.22 | 9 | 1.1 | 9 |
| Y.S.R. | ANDHRA PRADESH | 28.59 | 7 | 2.15 | 7 | 27.2 | 7 | 5.07 | 8 | 1.34 | 7 | 0.9 | 8 |
| KURNOOL | ANDHRA PRADESH | 25.20 | 3 | 1.61 | 3 | 39.6 | 9 | 6.72 | 10 | 2.27 | 9 | 2.1 | 10 |
| ANANTAPUR | ANDHRA PRADESH | 25.73 | 3 | 1.77 | 3 | 30.1 | 5 | 6.66 | 4 | 1.04 | 5 | 0.2 | 4 |
| CHITTOOR | ANDHRA PRADESH | 27.61 | 6 | 1.33 | 6 | 25.5 | 7 | 5.02 | 6 | 1.34 | 7 | 0.5 | 6 |
| BELGAUM | KARNATAKA | 29.53 | 7 | 1.54 | 7 | 36.9 | 7 | 7.29 | 7 | 1.42 | 7 | 0.6 | 7 |
| BAGALKOT | KARNATAKA | 22.33 | 1 | 1.32 | 1 | 43.4 | 6 | 8.52 | 7 | 1.26 | 6 | 0.7 | 7 |
| BIJAPUR | KARNATAKA | 24.45 | 2 | 1.93 | 2 | 36.9 | 9 | 9.75 | 9 | 2.20 | 9 | 1.1 | 9 |
| BIDAR | KARNATAKA | 27.59 | 6 | 2.13 | 6 | 36.8 | 7 | 7.97 | 6 | 1.47 | 7 | 0.5 | 6 |
| RAICHUR | KARNATAKA | 24.97 | 3 | 1.64 | 3 | 43.3 | 10 | 8.73 | 10 | 3.10 | 10 | 2.4 | 10 |
| KOPPAL | KARNATAKA | 23.84 | 2 | 1.58 | 2 | 42.3 | 9 | 8.75 | 9 | 2.28 | 9 | 1.3 | 9 |
| GADAG | KARNATAKA | 27.18 | 5 | 2.02 | 5 | 38.2 | 7 | 9.20 | 7 | 1.44 | 7 | 0.6 | 7 |
| DHARWAD | KARNATAKA | 24.89 | 2 | 1.49 | 2 | 35.4 | 10 | 6.69 | 9 | 2.65 | 10 | 1.3 | 9 |
| UTTARA KANNADA | KARNATAKA | 29.61 | 7 | 1.34 | 7 | 33.9 | 6 | 6.80 | 5 | 1.24 | 6 | 0.3 | 5 |
| HAVERI | KARNATAKA | 26.42 | 4 | 1.70 | 4 | 34.8 | 9 | 7.33 | 9 | 2.23 | 9 | 1.3 | 9 |
| BELLARY | KARNATAKA | 32.14 | 9 | 2.14 | 9 | 30.1 | 6 | 8.26 | 5 | 1.24 | 6 | 0.4 | 5 |
| CHITRADURGA | KARNATAKA | 29.00 | 7 | 2.15 | 7 | 25.1 | 10 | 4.78 | 10 | 2.56 | 10 | 1.7 | 10 |
| DAVANAGERE | KARNATAKA | 25.31 | 3 | 1.89 | 3 | 38.3 | 10 | 6.49 | 10 | 2.63 | 10 | 2.0 | 10 |
| SHIMOGA | KARNATAKA | 29.60 | 7 | 2.24 | 7 | 35.2 | 6 | 6.99 | 5 | 1.15 | 6 | 0.3 | 5 |
| UDUPI | KARNATAKA | 28.66 | 7 | 2.08 | 7 | 27.6 | 5 | 6.08 | 3 | 1.00 | 5 | 0.2 | 3 |
| CHIKMAGALUR | KARNATAKA | 26.18 | 4 | 1.37 | 4 | 25.2 | 7 | 4.90 | 7 | 1.51 | 7 | 0.7 | 7 |
| TUMKUR | KARNATAKA | 29.32 | 7 | 1.72 | 7 | 32.1 | 8 | 6.25 | 7 | 1.79 | 8 | 0.7 | 7 |
| BANGALORE | KARNATAKA | 27.50 | 6 | 1.91 | 6 | 27.6 | 8 | 5.44 | 6 | 1.59 | 8 | 0.5 | 6 |
| MANDYA | KARNATAKA | 25.61 | 3 | 1.11 | 3 | 30.7 | 6 | 5.74 | 4 | 1.21 | 6 | 0.2 | 4 |
| HASSAN | KARNATAKA | 26.21 | 4 | 1.77 | 4 | 33.3 | 5 | 7.98 | 1 | 0.99 | 5 | 0.0 | 1 |
| DAKSHINA KANNADA | KARNATAKA | 24.00 | 2 | 1.37 | 2 | 28.2 | 8 | 7.18 | 9 | 1.77 | 8 | 1.3 | 9 |
| KODAGU | KARNATAKA | 28.74 | 7 | 1.96 | 7 | 22.8 | 6 | 5.10 | 5 | 1.17 | 6 | 0.3 | 5 |
| MYSORE | KARNATAKA | 26.34 | 4 | 1.69 | 4 | 29.6 | 8 | 7.06 | 7 | 1.68 | 8 | 0.6 | 7 |
| CHAMARAJANAGAR | KARNATAKA | 28.35 | 6 | 1.67 | 6 | 33.9 | 8 | 6.14 | 7 | 1.78 | 8 | 0.6 | 7 |
| GULBARGA | KARNATAKA | 23.47 | 1 | 1.61 | 1 | 45.8 | 10 | 8.93 | 10 | 2.48 | 10 | 2.4 | 10 |
| YADGIR | KARNATAKA | 26.47 | 4 | 2.17 | 4 | 44.5 | 10 | 8.93 | 9 | 2.57 | 10 | 1.4 | 9 |
| KOLAR | KARNATAKA | 26.02 | 4 | 1.52 | 4 | 30.6 | 6 | 7.63 | 5 | 1.25 | 6 | 0.3 | 5 |
| CHIKKABALLAPURA | KARNATAKA | 26.18 | 4 | 1.40 | 4 | 30.9 | 6 | 6.36 | 5 | 1.22 | 6 | 0.3 | 5 |
| BANGALORE RURAL | KARNATAKA | 28.33 | 6 | 1.54 | 6 | 29.1 | 5 | 5.76 | 4 | 1.07 | 5 | 0.2 | 4 |
| RAMANAGARA | KARNATAKA | 30.01 | 8 | 1.53 | 8 | 26.0 | 5 | 4.76 | 1 | 1.04 | 5 | 0.0 | 1 |
| NORTH GOA | GOA | 25.59 | 3 | 1.36 | 3 | 27.0 | 6 | 5.99 | 6 | 1.21 | 6 | 0.5 | 6 |
| SOUTH GOA | GOA | 27.03 | 5 | 1.40 | 5 | 21.2 | 4 | 3.88 | 3 | 0.81 | 4 | 0.2 | 3 |
| LAKSHADWEEP | LAKSHADWEEP | 30.32 | 8 | 2.46 | 8 | 10.8 | 2 | 3.60 | 2 | 0.47 | 2 | 0.1 | 2 |
| KASARAGOD | KERALA | 20.58 | 1 | 1.24 | 1 | 13.4 | 2 | 4.54 | 4 | 0.56 | 2 | 0.2 | 4 |
| KANNUR | KERALA | 22.51 | 1 | 1.24 | 1 | 12.5 | 2 | 3.41 | 3 | 0.47 | 2 | 0.1 | 3 |
| WAYANAD | KERALA | 23.49 | 1 | 1.57 | 1 | 13.0 | 2 | 2.31 | 2 | 0.45 | 2 | 0.1 | 2 |
| KOZHIKODE | KERALA | 20.10 | 1 | 1.21 | 1 | 12.5 | 2 | 4.16 | 2 | 0.42 | 2 | 0.1 | 2 |
| MALAPPURAM | KERALA | 28.27 | 6 | 2.29 | 6 | 11.1 | 1 | 2.95 | 2 | 0.39 | 1 | 0.1 | 2 |
| PALAKKAD | KERALA | 26.00 | 4 | 1.41 | 4 | 15.6 | 2 | 4.14 | 2 | 0.43 | 2 | 0.1 | 2 |
| THRISSUR | KERALA | 23.80 | 2 | 1.64 | 2 | 15.7 | 2 | 4.81 | 2 | 0.45 | 2 | 0.1 | 2 |
| ERNAKULAM | KERALA | 21.92 | 1 | 1.04 | 1 | 13.3 | 1 | 4.39 | 1 | 0.38 | 1 | 0.0 | 1 |
| IDUKKI | KERALA | 21.29 | 1 | 1.12 | 1 | 10.1 | 2 | 2.33 | 3 | 0.52 | 2 | 0.1 | 3 |
| KOTTAYAM | KERALA | 22.80 | 1 | 1.16 | 1 | 10.6 | 3 | 1.79 | 3 | 0.57 | 3 | 0.2 | 3 |
| ALAPPUZHA | KERALA | 21.82 | 1 | 0.97 | 1 | 10.4 | 1 | 2.36 | 1 | 0.39 | 1 | 0.0 | 1 |
| PATHANAMTHITTA | KERALA | 24.11 | 2 | 1.28 | 2 | 13.0 | 2 | 2.19 | 2 | 0.48 | 2 | 0.1 | 2 |
| KOLLAM | KERALA | 19.52 | 1 | 0.92 | 1 | 15.3 | 1 | 3.48 | 1 | 0.38 | 1 | 0.0 | 1 |
| THIRUVANANTHAPURAM | KERALA | 21.85 | 1 | 1.03 | 1 | 12.5 | 2 | 2.53 | 2 | 0.47 | 2 | 0.1 | 2 |
| THIRUVALLUR | TAMIL NADU | 27.07 | 5 | 1.46 | 5 | 28.3 | 4 | 5.19 | 3 | 0.85 | 4 | 0.2 | 3 |
| CHENNAI | TAMIL NADU | 29.58 | 7 | 1.59 | 7 | 18.2 | 4 | 3.13 | 1 | 0.84 | 4 | 0.0 | 1 |
| KANCHEEPURAM | TAMIL NADU | 29.20 | 7 | 1.72 | 7 | 30.4 | 5 | 5.23 | 3 | 0.90 | 5 | 0.2 | 3 |
| VELLORE | TAMIL NADU | 27.59 | 6 | 1.95 | 6 | 26.7 | 6 | 6.15 | 5 | 1.16 | 6 | 0.4 | 5 |
| TIRUVANNAMALAI | TAMIL NADU | 26.17 | 4 | 1.46 | 4 | 31.2 | 6 | 7.06 | 7 | 1.12 | 6 | 0.6 | 7 |
| VILUPPURAM | TAMIL NADU | 26.16 | 4 | 1.47 | 4 | 35.0 | 9 | 6.38 | 9 | 2.14 | 9 | 1.3 | 9 |
| SALEM | TAMIL NADU | 24.70 | 2 | 1.21 | 2 | 24.3 | 5 | 4.31 | 5 | 1.07 | 5 | 0.3 | 5 |
| NAMAKKAL | TAMIL NADU | 26.64 | 5 | 1.32 | 5 | 31.0 | 8 | 5.76 | 8 | 1.66 | 8 | 0.8 | 8 |
| ERODE | TAMIL NADU | 23.88 | 2 | 1.26 | 2 | 22.1 | 5 | 4.14 | 5 | 1.05 | 5 | 0.3 | 5 |
| THE NILGIRIS | TAMIL NADU | 24.25 | 2 | 1.07 | 2 | 16.6 | 4 | 3.76 | 1 | 0.80 | 4 | 0.0 | 1 |
| DINDIGUL | TAMIL NADU | 24.61 | 2 | 1.60 | 2 | 23.1 | 5 | 6.35 | 5 | 0.96 | 5 | 0.3 | 5 |
| KARUR | TAMIL NADU | 24.42 | 2 | 1.00 | 2 | 39.0 | 7 | 5.90 | 6 | 1.47 | 7 | 0.5 | 6 |
| TIRUCHIRAPPALLI | TAMIL NADU | 26.00 | 4 | 1.67 | 4 | 43.8 | 7 | 8.53 | 6 | 1.46 | 7 | 0.5 | 6 |
| PERAMBALUR | TAMIL NADU | 24.33 | 2 | 1.46 | 2 | 38.1 | 7 | 6.49 | 6 | 1.37 | 7 | 0.5 | 6 |
| ARIYALUR | TAMIL NADU | 29.41 | 7 | 1.67 | 7 | 26.8 | 4 | 5.81 | 3 | 0.87 | 4 | 0.2 | 3 |
| CUDDALORE | TAMIL NADU | 25.44 | 3 | 1.27 | 3 | 33.6 | 7 | 8.15 | 6 | 1.49 | 7 | 0.5 | 6 |
| NAGAPATTINAM | TAMIL NADU | 23.90 | 2 | 1.59 | 2 | 35.0 | 5 | 7.14 | 4 | 0.95 | 5 | 0.2 | 4 |
| THIRUVARUR | TAMIL NADU | 25.70 | 3 | 1.32 | 3 | 18.0 | 4 | 4.28 | 1 | 0.78 | 4 | 0.0 | 1 |
| THANJAVUR | TAMIL NADU | 25.02 | 3 | 1.24 | 3 | 33.1 | 6 | 5.67 | 8 | 1.24 | 6 | 0.7 | 8 |
| PUDUKKOTTAI | TAMIL NADU | 22.88 | 1 | 1.28 | 1 | 41.6 | 6 | 9.05 | 8 | 1.26 | 6 | 0.8 | 8 |
| SIVAGANGA | TAMIL NADU | 25.48 | 3 | 1.26 | 3 | 29.7 | 5 | 5.53 | 4 | 0.92 | 5 | 0.2 | 4 |
| MADURAI | TAMIL NADU | 24.24 | 2 | 1.49 | 2 | 25.6 | 8 | 6.23 | 8 | 1.66 | 8 | 0.9 | 8 |
| THENI | TAMIL NADU | 23.16 | 1 | 1.58 | 1 | 21.9 | 7 | 5.51 | 7 | 1.49 | 7 | 0.7 | 7 |
| VIRUDHUNAGAR | TAMIL NADU | 30.21 | 8 | 1.83 | 8 | 22.5 | 4 | 5.01 | 3 | 0.84 | 4 | 0.1 | 3 |
| RAMANATHAPURAM | TAMIL NADU | 26.78 | 5 | 1.80 | 5 | 22.7 | 8 | 6.03 | 10 | 1.84 | 8 | 1.6 | 10 |
| THOOTHUKKUDI | TAMIL NADU | 28.18 | 6 | 1.90 | 6 | 22.1 | 5 | 4.54 | 5 | 1.04 | 5 | 0.3 | 5 |
| TIRUNELVELI | TAMIL NADU | 25.37 | 3 | 1.31 | 3 | 21.4 | 6 | 5.88 | 9 | 1.12 | 6 | 1.3 | 9 |
| KANNIYAKUMARI | TAMIL NADU | 25.23 | 3 | 1.22 | 3 | 15.2 | 5 | 3.17 | 5 | 1.03 | 5 | 0.3 | 5 |
| DHARMAPURI | TAMIL NADU | 24.73 | 2 | 1.38 | 2 | 23.4 | 7 | 6.67 | 6 | 1.49 | 7 | 0.6 | 6 |
| KRISHNAGIRI | TAMIL NADU | 26.44 | 4 | 1.71 | 4 | 20.2 | 4 | 3.75 | 4 | 0.89 | 4 | 0.2 | 4 |
| COIMBATORE | TAMIL NADU | 24.08 | 2 | 1.08 | 2 | 18.8 | 5 | 4.30 | 4 | 0.93 | 5 | 0.2 | 4 |
| TIRUPPUR | TAMIL NADU | 25.78 | 4 | 1.51 | 4 | 18.5 | 4 | 3.10 | 1 | 0.79 | 4 | 0.0 | 1 |
| YANAM | PUDUCHERRY | 24.72 | 2 | 1.53 | 2 | 16.2 | 3 | 3.76 | 3 | 0.69 | 3 | 0.2 | 3 |
| PUDUCHERRY | PUDUCHERRY | 24.79 | 2 | 1.09 | 2 | 35.3 | 3 | 6.93 | 2 | 0.63 | 3 | 0.1 | 2 |
| MAHE | PUDUCHERRY | 26.21 | 4 | 1.59 | 4 | 13.5 | 2 | 2.11 | 1 | 0.54 | 2 | 0.0 | 1 |
| KARAIKAL | PUDUCHERRY | 26.74 | 5 | 1.26 | 5 | 32.6 | 5 | 7.05 | 9 | 1.01 | 5 | 1.2 | 9 |
| NICOBARS | ANDAMAN & NICOBAR ISLANDS | 25.91 | 4 | 1.40 | 4 | 11.2 | 1 | 2.06 | 1 | 0.26 | 1 | 0.0 | 1 |
| NORTH & MIDDLE ANDAMAN | ANDAMAN & NICOBAR ISLANDS | 22.71 | 1 | 1.11 | 1 | 13.3 | 1 | 2.15 | 1 | 0.27 | 1 | 0.0 | 1 |
| SOUTH ANDAMAN | ANDAMAN & NICOBAR ISLANDS | 26.88 | 5 | 1.65 | 5 | 11.6 | 1 | 2.69 | 1 | 0.26 | 1 | 0.0 | 1 |
| EAST SIANG | ARUNACHAL PRADESH | 24.98 | 3 | 1.30 | 3 | 25.1 | 1 | 5.34 | 2 | 0.40 | 1 | 0.1 | 2 |
| KRA DAADI | ARUNACHAL PRADESH | 32.19 | 9 | 2.97 | 9 | 12.7 | 1 | 3.25 | 1 | 0.34 | 1 | 0.0 | 1 |
| KURUNG KUMEY | ARUNACHAL PRADESH | 24.58 | 2 | 1.82 | 2 | 28.0 | 1 | 8.93 | 1 | 0.34 | 1 | 0.0 | 1 |
| LOHIT | ARUNACHAL PRADESH | 31.46 | 9 | 2.38 | 9 | 14.9 | 2 | 4.90 | 3 | 0.50 | 2 | 0.2 | 3 |
| LONGDING | ARUNACHAL PRADESH | 25.41 | 3 | 1.68 | 3 | 12.7 | 1 | 3.23 | 2 | 0.38 | 1 | 0.1 | 2 |
| NAMSAI | ARUNACHAL PRADESH | 30.97 | 8 | 2.07 | 8 | 21.9 | 1 | 7.09 | 2 | 0.41 | 1 | 0.1 | 2 |
| SIANG | ARUNACHAL PRADESH | 20.64 | 1 | 1.04 | 1 | 11.2 | 2 | 3.46 | 4 | 0.45 | 2 | 0.3 | 4 |
| TIRAP | ARUNACHAL PRADESH | 30.91 | 8 | 2.43 | 8 | 13.1 | 1 | 2.94 | 2 | 0.37 | 1 | 0.1 | 2 |
| WEST SIANG | ARUNACHAL PRADESH | 26.75 | 5 | 2.51 | 5 | 13.3 | 1 | 4.96 | 1 | 0.32 | 1 | 0.0 | 1 |
| BISWANATH | ASSAM | 36.66 | 10 | 2.30 | 10 | 39.2 | 2 | 9.38 | 3 | 0.51 | 2 | 0.1 | 3 |
| CHARAIDEO | ASSAM | 37.27 | 10 | 3.03 | 10 | 31.1 | 2 | 8.61 | 3 | 0.51 | 2 | 0.2 | 3 |
| DHUBRI | ASSAM | 35.41 | 10 | 2.95 | 10 | 34.1 | 2 | 11.13 | 3 | 0.53 | 2 | 0.2 | 3 |
| HOJAI | ASSAM | 32.42 | 9 | 2.21 | 9 | 28.2 | 2 | 6.65 | 2 | 0.42 | 2 | 0.1 | 2 |
| JORHAT | ASSAM | 37.62 | 10 | 2.63 | 10 | 31.7 | 3 | 7.81 | 6 | 0.70 | 3 | 0.4 | 6 |
| KARBI ANGLONG | ASSAM | 37.41 | 10 | 2.85 | 10 | 27.7 | 1 | 9.49 | 1 | 0.36 | 1 | 0.0 | 1 |
| MAJULI | ASSAM | 40.75 | 10 | 2.38 | 10 | 24.9 | 4 | 6.35 | 6 | 0.85 | 4 | 0.5 | 6 |
| NAGAON | ASSAM | 37.39 | 10 | 2.70 | 10 | 31.2 | 2 | 8.36 | 2 | 0.45 | 2 | 0.1 | 2 |
| SIVASAGAR | ASSAM | 36.51 | 10 | 1.88 | 10 | 31.1 | 1 | 6.30 | 1 | 0.38 | 1 | 0.0 | 1 |
| SONITPUR | ASSAM | 38.08 | 10 | 2.41 | 10 | 28.7 | 2 | 7.82 | 3 | 0.51 | 2 | 0.1 | 3 |
| SOUTH SALMARA MANCACHAR | ASSAM | 38.49 | 10 | 3.15 | 10 | 32.2 | 1 | 11.56 | 2 | 0.39 | 1 | 0.1 | 2 |
| WEST KARBI ANGLONG | ASSAM | 38.41 | 10 | 3.26 | 10 | 37.4 | 1 | 9.89 | 2 | 0.41 | 1 | 0.1 | 2 |
| BALOD | CHHATTISGARH | 28.28 | 6 | 1.51 | 6 | 37.8 | 4 | 8.31 | 3 | 0.76 | 4 | 0.2 | 3 |
| BALODA BAZAR | CHHATTISGARH | 26.28 | 4 | 1.86 | 4 | 41.3 | 6 | 10.34 | 6 | 1.12 | 6 | 0.4 | 6 |
| BALRAMPUR | CHHATTISGARH | 25.00 | 3 | 1.89 | 3 | 27.7 | 3 | 6.57 | 2 | 0.67 | 3 | 0.1 | 2 |
| BASTAR | CHHATTISGARH | 21.44 | 1 | 1.32 | 1 | 58.5 | 6 | 9.76 | 6 | 1.13 | 6 | 0.4 | 6 |
| BEMETARA | CHHATTISGARH | 28.88 | 7 | 2.12 | 7 | 30.0 | 6 | 9.39 | 7 | 1.20 | 6 | 0.7 | 7 |
| BILASPUR | CHHATTISGARH | 26.54 | 4 | 1.48 | 4 | 44.0 | 8 | 8.51 | 10 | 1.56 | 8 | 1.6 | 10 |
| DANTEWADA | CHHATTISGARH | 22.67 | 1 | 1.76 | 1 | 59.6 | 10 | 8.64 | 10 | 2.96 | 10 | 3.7 | 10 |
| DURG | CHHATTISGARH | 23.75 | 2 | 1.59 | 2 | 33.1 | 6 | 8.66 | 9 | 1.23 | 6 | 1.1 | 9 |
| GARIYABAND | CHHATTISGARH | 27.05 | 5 | 2.11 | 5 | 42.0 | 5 | 9.42 | 6 | 1.08 | 5 | 0.5 | 6 |
| KODAGAON | CHHATTISGARH | 23.95 | 2 | 1.78 | 2 | 54.0 | 5 | 9.55 | 6 | 1.06 | 5 | 0.4 | 6 |
| MUNGELI | CHHATTISGARH | 30.15 | 8 | 2.24 | 8 | 31.9 | 3 | 7.29 | 1 | 0.65 | 3 | 0.0 | 1 |
| RAIPUR | CHHATTISGARH | 32.04 | 9 | 3.22 | 9 | 37.8 | 4 | 10.76 | 4 | 0.86 | 4 | 0.2 | 4 |
| SUKMA | CHHATTISGARH | 26.66 | 5 | 1.70 | 5 | 58.1 | 8 | 8.61 | 9 | 1.65 | 8 | 1.5 | 9 |
| SURAJPUR | CHHATTISGARH | 29.64 | 7 | 2.10 | 7 | 22.0 | 3 | 5.84 | 2 | 0.66 | 3 | 0.1 | 2 |
| SURGUJA | CHHATTISGARH | 26.57 | 5 | 1.63 | 5 | 24.7 | 6 | 5.51 | 8 | 1.24 | 6 | 0.9 | 8 |
| CENTRAL | NCT OF DELHI | 24.25 | 2 | 1.34 | 2 | 40.1 | 10 | 7.32 | 10 | 3.57 | 10 | 2.1 | 10 |
| EAST | NCT OF DELHI | 25.82 | 4 | 1.79 | 4 | 35.0 | 9 | 7.21 | 9 | 2.25 | 9 | 1.0 | 9 |
| NEW DELHI | NCT OF DELHI | 21.17 | 1 | 1.39 | 1 | 47.9 | 10 | 8.42 | 10 | 4.28 | 10 | 2.9 | 10 |
| NORTH | NCT OF DELHI | 23.49 | 1 | 1.45 | 1 | 39.4 | 10 | 8.20 | 8 | 2.53 | 10 | 0.9 | 8 |
| NORTH EAST | NCT OF DELHI | 22.25 | 1 | 1.24 | 1 | 38.5 | 10 | 7.65 | 10 | 4.17 | 10 | 2.4 | 10 |
| NORTH WEST | NCT OF DELHI | 24.91 | 3 | 1.54 | 3 | 42.8 | 10 | 7.68 | 9 | 2.76 | 10 | 1.3 | 9 |
| SHAHDARA | NCT OF DELHI | 29.17 | 7 | 1.84 | 7 | 34.9 | 10 | 7.07 | 9 | 2.84 | 10 | 1.1 | 9 |
| SOUTH | NCT OF DELHI | 26.86 | 5 | 1.82 | 5 | 34.6 | 8 | 7.90 | 6 | 1.74 | 8 | 0.4 | 6 |
| SOUTH EAST | NCT OF DELHI | 30.74 | 8 | 1.92 | 8 | 33.5 | 9 | 7.17 | 9 | 2.37 | 9 | 1.3 | 9 |
| SOUTH WEST | NCT OF DELHI | 26.56 | 5 | 1.98 | 5 | 38.4 | 9 | 6.75 | 7 | 2.16 | 9 | 0.7 | 7 |
| WEST | NCT OF DELHI | 27.50 | 6 | 2.18 | 6 | 31.1 | 10 | 6.78 | 10 | 3.64 | 10 | 3.0 | 10 |
| AHMADABAD | GUJARAT | 26.38 | 4 | 1.58 | 4 | 41.8 | 9 | 7.14 | 9 | 2.42 | 9 | 1.1 | 9 |
| ARAVALI | GUJARAT | 22.92 | 1 | 1.67 | 1 | 60.3 | 10 | 7.74 | 10 | 3.93 | 10 | 2.1 | 10 |
| BHAVNAGAR | GUJARAT | 26.94 | 5 | 1.69 | 5 | 42.8 | 8 | 6.64 | 7 | 1.76 | 8 | 0.6 | 7 |
| BOTAD | GUJARAT | 25.16 | 3 | 1.30 | 3 | 51.4 | 9 | 8.22 | 8 | 1.97 | 9 | 0.9 | 8 |
| CHHOTA UDAIPUR | GUJARAT | 26.14 | 4 | 1.77 | 4 | 57.0 | 7 | 9.21 | 10 | 1.49 | 7 | 1.8 | 10 |
| DEVBHUMI DWARKA | GUJARAT | 29.56 | 7 | 2.33 | 7 | 33.9 | 5 | 8.32 | 4 | 0.95 | 5 | 0.2 | 4 |
| GIR SOMNATH | GUJARAT | 28.75 | 7 | 2.01 | 7 | 36.7 | 8 | 7.57 | 7 | 1.60 | 8 | 0.7 | 7 |
| JAMNAGAR | GUJARAT | 31.11 | 8 | 1.77 | 8 | 41.4 | 7 | 7.66 | 5 | 1.39 | 7 | 0.3 | 5 |
| JUNAGADH | GUJARAT | 25.10 | 3 | 1.69 | 3 | 47.2 | 9 | 7.69 | 8 | 2.06 | 9 | 0.8 | 8 |
| KHEDA | GUJARAT | 27.43 | 5 | 1.97 | 5 | 51.4 | 9 | 8.23 | 8 | 2.09 | 9 | 0.8 | 8 |
| MAHISAGAR | GUJARAT | 22.89 | 1 | 1.75 | 1 | 62.1 | 7 | 9.37 | 9 | 1.47 | 7 | 1.0 | 9 |
| MORBI | GUJARAT | 33.18 | 9 | 1.96 | 9 | 38.7 | 6 | 8.58 | 7 | 1.31 | 6 | 0.6 | 7 |
| PANCH MAHALS | GUJARAT | 26.04 | 4 | 2.05 | 4 | 60.0 | 9 | 8.26 | 10 | 1.99 | 9 | 1.6 | 10 |
| RAJKOT | GUJARAT | 27.20 | 5 | 1.97 | 5 | 48.4 | 7 | 9.96 | 8 | 1.49 | 7 | 0.9 | 8 |
| SABAR KANTHA | GUJARAT | 30.79 | 8 | 2.13 | 8 | 47.9 | 7 | 7.76 | 6 | 1.51 | 7 | 0.6 | 6 |
| SURENDRANAGAR | GUJARAT | 31.28 | 9 | 2.32 | 9 | 45.2 | 6 | 8.27 | 4 | 1.11 | 6 | 0.2 | 4 |
| VADODARA | GUJARAT | 27.73 | 6 | 1.46 | 6 | 53.5 | 8 | 8.23 | 7 | 1.80 | 8 | 0.6 | 7 |
| BHIWANI | HARYANA | 22.12 | 1 | 1.37 | 1 | 45.4 | 10 | 7.00 | 9 | 2.56 | 10 | 1.0 | 9 |
| CHARKHI DADRI | HARYANA | 27.72 | 6 | 1.44 | 6 | 35.8329 | 8 | 7.13 | 6 | 1.80 | 8 | 0.5 | 6 |
| AGAR MALWA | MADHYA PRADESH | 29.74 | 8 | 1.46 | 8 | 35.6443 | 8 | 5.13 | 6 | 1.65 | 8 | 0.5 | 6 |
| SHAJAPUR | MADHYA PRADESH | 25.59 | 3 | 2.07 | 3 | 44.8495 | 10 | 7.68 | 10 | 2.72 | 10 | 2.3 | 10 |
| PALGHAR | MAHARASHTRA | 29.88 | 8 | 1.79 | 8 | 38.7665 | 6 | 7.79 | 5 | 1.24 | 6 | 0.3 | 5 |
| THANE | MAHARASHTRA | 29.70 | 8 | 1.43 | 8 | 35.4886 | 7 | 5.68 | 5 | 1.33 | 7 | 0.3 | 5 |
| EAST GARO HILLS | MEGHALAYA | 19.13 | 1 | 1.98 | 1 | 6.69884 | 1 | 1.99 | 1 | 0.22 | 1 | 0.0 | 1 |
| EAST JANTIA HILLS | MEGHALAYA | 24.34 | 2 | 2.32 | 2 | 16.5646 | 1 | 6.68 | 2 | 0.29 | 1 | 0.1 | 2 |
| NORTH GARO HILLS | MEGHALAYA | 20.61 | 1 | 2.43 | 1 | 9.04456 | 1 | 3.47 | 1 | 0.24 | 1 | 0.0 | 1 |
| SOUTH WEST GARO HILLS | MEGHALAYA | 23.75 | 2 | 1.98 | 2 | 11.8429 | 1 | 4.75 | 1 | 0.23 | 1 | 0.0 | 1 |
| SOUTH WEST KHASI HILLS | MEGHALAYA | 25.05 | 3 | 3.01 | 3 | 21.781 | 1 | 11.34 | 3 | 0.30 | 1 | 0.2 | 3 |
| WEST GARO HILLS | MEGHALAYA | 23.88 | 2 | 2.30 | 2 | 8.51363 | 1 | 2.07 | 1 | 0.24 | 1 | 0.0 | 1 |
| WEST JAINTIA HILLS | MEGHALAYA | 26.21 | 4 | 2.75 | 4 | 19.6393 | 1 | 7.81 | 2 | 0.29 | 1 | 0.1 | 2 |
| WEST KHASI HILLS | MEGHALAYA | 24.25 | 2 | 2.80 | 2 | 17.8796 | 1 | 7.48 | 2 | 0.29 | 1 | 0.1 | 2 |
| FAZILKA | PUNJAB | 23.33 | 1 | 1.30 | 1 | 39.5611 | 10 | 6.83 | 10 | 4.79 | 10 | 2.9 | 10 |
| FIROZPUR | PUNJAB | 26.38 | 4 | 1.78 | 4 | 36.5003 | 10 | 6.83 | 10 | 5.25 | 10 | 3.0 | 10 |
| GURDASPUR | PUNJAB | 26.13 | 4 | 1.85 | 4 | 39.4616 | 10 | 8.45 | 9 | 2.52 | 10 | 1.0 | 9 |
| PATHANKOT | PUNJAB | 24.97 | 3 | 1.52 | 3 | 40.946 | 9 | 9.01 | 7 | 2.08 | 9 | 0.6 | 7 |
| ADILABAD | TELANGANA | 25.24 | 3 | 1.55 | 3 | 42.2552 | 10 | 7.58 | 10 | 3.40 | 10 | 2.1 | 10 |
| BHADRADRI KOTHAGUDEM | TELANGANA | 23.21 | 1 | 1.36 | 1 | 45.2644 | 7 | 7.79 | 5 | 1.36 | 7 | 0.3 | 5 |
| HYDERABAD | TELANGANA | 23.88 | 2 | 1.03 | 2 | 39.7117 | 9 | 7.50 | 8 | 2.35 | 9 | 0.8 | 8 |
| JAGITIAL | TELANGANA | 24.29 | 2 | 1.83 | 2 | 35.6833 | 8 | 6.67 | 6 | 1.61 | 8 | 0.5 | 6 |
| JANGOAN | TELANGANA | 26.63 | 5 | 1.29 | 5 | 37.9637 | 7 | 7.31 | 4 | 1.37 | 7 | 0.2 | 4 |
| JAYASHANKAR BHUPALAPALLY | TELANGANA | 24.60 | 2 | 1.38 | 2 | 42.3442 | 8 | 7.85 | 6 | 1.77 | 8 | 0.6 | 6 |
| JOGULAMBA GADWAL | TELANGANA | 22.74 | 1 | 1.33 | 1 | 51.3711 | 10 | 6.42 | 10 | 4.05 | 10 | 2.6 | 10 |
| KAMAREDDY | TELANGANA | 25.18 | 3 | 1.98 | 3 | 42.2895 | 10 | 8.70 | 9 | 2.63 | 10 | 1.2 | 9 |
| KARIMNAGAR | TELANGANA | 24.84 | 2 | 1.21 | 2 | 39.4014 | 10 | 6.41 | 9 | 3.20 | 10 | 1.2 | 9 |
| KHAMMAM | TELANGANA | 25.74 | 3 | 1.48 | 3 | 33.6656 | 9 | 6.74 | 8 | 2.15 | 9 | 1.0 | 8 |
| KOMARAM BHEEM ASIFABAD | TELANGANA | 24.66 | 2 | 1.72 | 2 | 40.1152 | 9 | 8.40 | 10 | 2.43 | 9 | 1.6 | 10 |
| MAHABUBABAD | TELANGANA | 25.86 | 4 | 1.26 | 4 | 48.24 | 9 | 6.89 | 7 | 1.96 | 9 | 0.6 | 7 |
| MAHABUBNAGAR | TELANGANA | 26.43 | 4 | 1.85 | 4 | 48.1175 | 9 | 7.56 | 9 | 2.37 | 9 | 1.0 | 9 |
| MANCHERIAL | TELANGANA | 24.39 | 2 | 1.20 | 2 | 35.4133 | 8 | 6.22 | 8 | 1.58 | 8 | 0.7 | 8 |
| MEDAK | TELANGANA | 26.84 | 5 | 1.31 | 5 | 39.1303 | 9 | 7.56 | 7 | 2.07 | 9 | 0.7 | 7 |
| MEDCHAL-MALKAJGIRI | TELANGANA | 27.23 | 5 | 1.50 | 5 | 34.964 | 10 | 5.21 | 10 | 3.09 | 10 | 1.8 | 10 |
| NAGARKURNOOL | TELANGANA | 29.06 | 7 | 1.84 | 7 | 40.9664 | 8 | 7.01 | 6 | 1.58 | 8 | 0.4 | 6 |
| NALGONDA | TELANGANA | 25.75 | 3 | 1.34 | 3 | 42.9499 | 9 | 7.43 | 8 | 2.20 | 9 | 0.8 | 8 |
| NIRMAL | TELANGANA | 23.05 | 1 | 1.22 | 1 | 40.7069 | 10 | 6.60 | 10 | 3.42 | 10 | 1.8 | 10 |
| NIZAMABAD | TELANGANA | 26.34 | 4 | 1.61 | 4 | 37.4496 | 8 | 6.82 | 6 | 1.63 | 8 | 0.5 | 6 |
| PEDDAPALLI | TELANGANA | 24.38 | 2 | 1.11 | 2 | 40.569 | 9 | 6.66 | 8 | 2.15 | 9 | 0.7 | 8 |
| RAJANNA SIRCILLA | TELANGANA | 24.69 | 2 | 1.30 | 2 | 36.6039 | 10 | 5.48 | 9 | 2.74 | 10 | 1.5 | 9 |
| RANGA REDDY | TELANGANA | 23.98 | 2 | 1.55 | 2 | 47.7445 | 9 | 7.85 | 8 | 2.08 | 9 | 0.8 | 8 |
| SANGAREDDY | TELANGANA | 25.57 | 3 | 1.87 | 3 | 36.1031 | 9 | 7.25 | 8 | 2.14 | 9 | 0.8 | 8 |
| SIDDIPET | TELANGANA | 24.74 | 2 | 1.61 | 2 | 35.041 | 10 | 7.34 | 10 | 2.84 | 10 | 1.6 | 10 |
| SURYAPET | TELANGANA | 26.33 | 4 | 1.26 | 4 | 39.9863 | 10 | 5.83 | 10 | 3.60 | 10 | 1.9 | 10 |
| VIKARABAD | TELANGANA | 21.88 | 1 | 1.45 | 1 | 45.0523 | 10 | 6.78 | 9 | 2.91 | 10 | 1.1 | 9 |
| WANAPARTHY | TELANGANA | 24.63 | 2 | 1.61 | 2 | 46.0719 | 9 | 8.85 | 9 | 2.27 | 9 | 1.2 | 9 |
| WARANGAL RURAL | TELANGANA | 22.29 | 1 | 1.19 | 1 | 46.0388 | 9 | 7.94 | 9 | 2.00 | 9 | 1.0 | 9 |
| WARANGAL URBAN | TELANGANA | 24.37 | 2 | 1.44 | 2 | 37.361 | 9 | 6.88 | 8 | 2.37 | 9 | 0.9 | 8 |
| YADADRI BHUVANAGIRI | TELANGANA | 22.66 | 1 | 1.53 | 1 | 42.1187 | 9 | 8.38 | 7 | 2.12 | 9 | 0.7 | 7 |
| GOMATI | TRIPURA | 34.24 | 10 | 2.43 | 10 | 31.0966 | 2 | 7.96 | 2 | 0.43 | 2 | 0.1 | 2 |
| KHOWAI | TRIPURA | 35.65 | 10 | 1.94 | 10 | 29.8697 | 1 | 6.98 | 1 | 0.37 | 1 | 0.0 | 1 |
| NORTH TRIPURA | TRIPURA | 32.66 | 9 | 2.01 | 9 | 20.2629 | 1 | 6.22 | 2 | 0.39 | 1 | 0.1 | 2 |
| SEPAHIJALA | TRIPURA | 32.36 | 9 | 2.27 | 9 | 33.7655 | 3 | 6.47 | 5 | 0.58 | 3 | 0.3 | 5 |
| SOUTH TRIPURA | TRIPURA | 32.27 | 9 | 2.19 | 9 | 25.6858 | 3 | 7.16 | 3 | 0.57 | 3 | 0.2 | 3 |
| UNAKOTI | TRIPURA | 30.78 | 8 | 1.82 | 8 | 29.4769 | 3 | 6.57 | 5 | 0.61 | 3 | 0.4 | 5 |
| WEST TRIPURA | TRIPURA | 32.23 | 9 | 2.01 | 9 | 22.0508 | 1 | 6.57 | 1 | 0.38 | 1 | 0.0 | 1 |
| AMETHI | UTTAR PRADESH | 28.68 | 7 | 2.16 | 7 | 21.7461 | 5 | 5.75 | 5 | 1.00 | 5 | 0.3 | 5 |
| BUDAUN | UTTAR PRADESH | 27.17 | 5 | 2.30 | 5 | 42.7828 | 6 | 11.15 | 7 | 1.11 | 6 | 0.7 | 7 |
| GHAZIABAD | UTTAR PRADESH | 27.43 | 5 | 1.52 | 5 | 31.2627 | 7 | 5.17 | 6 | 1.36 | 7 | 0.4 | 6 |
| HAPUR | UTTAR PRADESH | 27.02 | 5 | 2.13 | 5 | 39.7599 | 4 | 11.68 | 4 | 0.77 | 4 | 0.2 | 4 |
| MORADABAD | UTTAR PRADESH | 27.71 | 6 | 1.96 | 6 | 35.5494 | 4 | 11.34 | 3 | 0.75 | 4 | 0.2 | 3 |
| MUZAFFARNAGAR | UTTAR PRADESH | 27.84 | 6 | 1.89 | 6 | 34.2141 | 6 | 9.44 | 7 | 1.21 | 6 | 0.6 | 7 |
| RAE BARELI | UTTAR PRADESH | 27.81 | 6 | 2.07 | 6 | 43.2068 | 6 | 8.70 | 6 | 1.11 | 6 | 0.5 | 6 |
| SAMBHAL | UTTAR PRADESH | 29.07 | 7 | 2.37 | 7 | 37.9803 | 7 | 11.49 | 9 | 1.41 | 7 | 1.4 | 9 |
| SHAMLI | UTTAR PRADESH | 31.67 | 9 | 2.16 | 9 | 31.9427 | 6 | 8.73 | 7 | 1.17 | 6 | 0.6 | 7 |
| SULTANPUR | UTTAR PRADESH | 25.09 | 3 | 1.75 | 3 | 35.7038 | 6 | 6.83 | 5 | 1.15 | 6 | 0.4 | 5 |
| PASCHIM BARDDHAMAN | WEST BENGAL | 35.35 | 10 | 2.78 | 10 | 34.7459 | 4 | 9.00 | 4 | 0.74 | 4 | 0.3 | 4 |
| PURBA BARDDHAMAN | WEST BENGAL | 31.70 | 9 | 1.62 | 9 | 36.0735 | 3 | 6.98 | 4 | 0.67 | 3 | 0.2 | 4 |

Table S7: Mean Prevalence and Standard Deviation of Anemia (Mild/Moderate and Severe) among Women (15-49 Years) by Districts, India, NFHS, 2021

| **DISTRICT NAME** | **STATE NAME** | **Mild Anemia - Prevalence (%)** | **Decile Position (Mild Anemia)** | **Mild Anemia - SD** | **Decile Position (Mild Anemia SD)** | **Moderate Anemia - Prevalence (%)** | **Decile Position (Moderate Anemia)** | **Moderate Anemia - SD** | **Decile Position (Moderate Anemia SD)** | **Severe Anemia -Prevalence (%)** | **Decile Position (Severe Anemia)** | **Severe Anemia - SD** | **Decile Position (Severe Anemia SD)** |
| --- | --- | --- | --- | --- | --- | --- | --- | --- | --- | --- | --- | --- | --- |
| KUPWARA | JAMMU & KASHMIR | 25.38 | 6 | 2.48 | 10 | 42.27 | 8 | 9.60 | 10 | 2.50 | 8.0 | 3.87 | 10 |
| BADGAM | JAMMU & KASHMIR | 24.50 | 5 | 2.38 | 10 | 46.17 | 4 | 11.45 | 6 | 1.66 | 4.0 | 0.76 | 6 |
| LEH(LADAKH) | LADAKH | 12.48 | 1 | 1.33 | 2 | 44.13 | 10 | 16.00 | 10 | 32.13 | 10.0 | 28.80 | 10 |
| KARGIL | LADAKH | 14.96 | 1 | 1.81 | 7 | 53.23 | 10 | 12.19 | 10 | 18.46 | 10.0 | 19.54 | 10 |
| PUNCH | JAMMU & KASHMIR | 22.44 | 3 | 1.75 | 6 | 29.91 | 2 | 9.72 | 8 | 1.16 | 2.0 | 1.06 | 8 |
| RAJOURI | JAMMU & KASHMIR | 25.53 | 6 | 2.01 | 9 | 32.35 | 3 | 11.80 | 5 | 1.43 | 3.0 | 0.66 | 5 |
| KATHUA | JAMMU & KASHMIR | 22.27 | 3 | 2.23 | 10 | 30.80 | 1 | 9.01 | 1 | 0.82 | 1.0 | 0.18 | 1 |
| BARAMULA | JAMMU & KASHMIR | 25.84 | 7 | 2.14 | 10 | 39.59 | 10 | 8.54 | 10 | 4.45 | 10.0 | 2.87 | 10 |
| BANDIPORE | JAMMU & KASHMIR | 23.40 | 4 | 2.07 | 9 | 41.12 | 10 | 10.12 | 10 | 5.44 | 10.0 | 7.06 | 10 |
| SRINAGAR | JAMMU & KASHMIR | 21.57 | 2 | 1.62 | 5 | 30.76 | 3 | 8.55 | 6 | 1.42 | 3.0 | 0.71 | 6 |
| GANDERBAL | JAMMU & KASHMIR | 22.75 | 3 | 1.73 | 6 | 45.05 | 10 | 9.17 | 10 | 5.77 | 10.0 | 3.74 | 10 |
| PULWAMA | JAMMU & KASHMIR | 25.61 | 6 | 1.79 | 7 | 43.93 | 3 | 10.91 | 4 | 1.43 | 3.0 | 0.57 | 4 |
| SHUPIYAN | JAMMU & KASHMIR | 28.15 | 8 | 2.71 | 10 | 32.85 | 1 | 8.69 | 1 | 0.87 | 1.0 | 0.19 | 1 |
| ANANTNAG | JAMMU & KASHMIR | 26.36 | 7 | 2.58 | 10 | 42.83 | 5 | 9.25 | 7 | 1.83 | 5.0 | 0.94 | 7 |
| KULGAM | JAMMU & KASHMIR | 27.81 | 8 | 2.42 | 10 | 43.21 | 6 | 10.65 | 10 | 1.97 | 6.0 | 1.94 | 10 |
| DODA | JAMMU & KASHMIR | 23.21 | 3 | 1.86 | 8 | 38.28 | 10 | 7.40 | 10 | 3.50 | 10.0 | 3.51 | 10 |
| RAMBAN | JAMMU & KASHMIR | 23.91 | 5 | 2.12 | 9 | 33.64 | 2 | 9.37 | 2 | 1.09 | 2.0 | 0.29 | 2 |
| KISHTWAR | JAMMU & KASHMIR | 20.28 | 1 | 2.01 | 9 | 51.83 | 10 | 12.24 | 10 | 7.43 | 10.0 | 18.16 | 10 |
| UDHAMPUR | JAMMU & KASHMIR | 26.05 | 7 | 1.80 | 7 | 27.55 | 3 | 7.18 | 5 | 1.32 | 3.0 | 0.63 | 5 |
| REASI | JAMMU & KASHMIR | 22.50 | 3 | 1.71 | 6 | 37.04 | 6 | 10.26 | 10 | 2.00 | 6.0 | 4.94 | 10 |
| JAMMU | JAMMU & KASHMIR | 27.57 | 8 | 2.01 | 9 | 35.46 | 2 | 8.49 | 3 | 1.24 | 2.0 | 0.44 | 3 |
| SAMBA | JAMMU & KASHMIR | 25.19 | 6 | 1.73 | 6 | 35.69 | 2 | 7.71 | 2 | 1.20 | 2.0 | 0.35 | 2 |
| CHAMBA | HIMACHAL PRADESH | 24.57 | 5 | 1.98 | 9 | 18.03 | 2 | 4.47 | 2 | 1.13 | 2.0 | 0.26 | 2 |
| KANGRA | HIMACHAL PRADESH | 28.16 | 8 | 1.64 | 5 | 29.82 | 4 | 6.13 | 4 | 1.66 | 4.0 | 0.54 | 4 |
| LAHUL & SPITI | HIMACHAL PRADESH | 22.19 | 3 | 1.35 | 2 | 47.43 | 10 | 7.97 | 10 | 7.34 | 10.0 | 8.90 | 10 |
| KULLU | HIMACHAL PRADESH | 27.46 | 8 | 1.89 | 8 | 21.29 | 3 | 6.94 | 5 | 1.47 | 3.0 | 0.61 | 5 |
| MANDI | HIMACHAL PRADESH | 26.61 | 7 | 1.66 | 5 | 20.26 | 2 | 5.09 | 1 | 1.18 | 2.0 | 0.24 | 1 |
| HAMIRPUR | HIMACHAL PRADESH | 25.54 | 6 | 1.69 | 5 | 21.07 | 4 | 3.18 | 4 | 1.60 | 4.0 | 0.50 | 4 |
| UNA | HIMACHAL PRADESH | 27.00 | 8 | 1.29 | 1 | 28.48 | 6 | 5.94 | 5 | 1.86 | 6.0 | 0.66 | 5 |
| BILASPUR | HIMACHAL PRADESH | 25.68 | 6 | 1.52 | 4 | 24.15 | 4 | 5.09 | 6 | 1.62 | 4.0 | 0.76 | 6 |
| SOLAN | HIMACHAL PRADESH | 24.18 | 5 | 2.01 | 9 | 20.30 | 5 | 5.05 | 5 | 1.79 | 5.0 | 0.62 | 5 |
| SIRMAUR | HIMACHAL PRADESH | 25.24 | 6 | 2.08 | 9 | 21.67 | 3 | 5.40 | 3 | 1.32 | 3.0 | 0.46 | 3 |
| SHIMLA | HIMACHAL PRADESH | 25.04 | 6 | 1.97 | 9 | 19.06 | 3 | 5.14 | 3 | 1.47 | 3.0 | 0.47 | 3 |
| KINNAUR | HIMACHAL PRADESH | 23.53 | 4 | 1.69 | 5 | 25.85 | 7 | 7.94 | 10 | 2.25 | 7.0 | 2.51 | 10 |
| KAPURTHALA | PUNJAB | 23.74 | 4 | 1.51 | 3 | 27.09 | 8 | 5.53 | 6 | 2.38 | 8.0 | 0.80 | 6 |
| JALANDHAR | PUNJAB | 26.09 | 7 | 1.74 | 6 | 27.59 | 6 | 4.73 | 6 | 1.96 | 6.0 | 0.68 | 6 |
| HOSHIARPUR | PUNJAB | 24.61 | 5 | 1.83 | 7 | 27.46 | 3 | 5.21 | 2 | 1.37 | 3.0 | 0.32 | 2 |
| SHAHID BHAGAT SINGH NAGAR | PUNJAB | 23.91 | 5 | 1.55 | 4 | 24.35 | 3 | 4.77 | 3 | 1.34 | 3.0 | 0.45 | 3 |
| FATEHGARH SAHIB | PUNJAB | 25.62 | 6 | 1.36 | 2 | 32.76 | 9 | 4.85 | 8 | 3.02 | 9.0 | 1.06 | 8 |
| LUDHIANA | PUNJAB | 23.72 | 4 | 1.06 | 1 | 34.36 | 9 | 6.97 | 9 | 2.98 | 9.0 | 1.48 | 9 |
| MOGA | PUNJAB | 23.56 | 4 | 1.67 | 5 | 26.82 | 8 | 6.35 | 7 | 2.41 | 8.0 | 0.86 | 7 |
| MUKTSAR | PUNJAB | 23.52 | 4 | 1.67 | 5 | 33.17 | 9 | 5.82 | 8 | 3.23 | 9.0 | 1.10 | 8 |
| FARIDKOT | PUNJAB | 23.77 | 4 | 1.68 | 5 | 34.51 | 9 | 7.00 | 6 | 2.72 | 9.0 | 0.76 | 6 |
| BATHINDA | PUNJAB | 25.34 | 6 | 1.64 | 5 | 28.33 | 8 | 4.72 | 8 | 2.68 | 8.0 | 0.99 | 8 |
| MANSA | PUNJAB | 23.63 | 4 | 1.85 | 7 | 33.14 | 8 | 7.16 | 5 | 2.34 | 8.0 | 0.66 | 5 |
| PATIALA | PUNJAB | 25.33 | 6 | 1.62 | 5 | 33.12 | 9 | 8.38 | 8 | 2.81 | 9.0 | 1.05 | 8 |
| AMRITSAR | PUNJAB | 23.55 | 4 | 1.69 | 5 | 26.56 | 4 | 5.67 | 3 | 1.53 | 4.0 | 0.44 | 3 |
| TARN TARAN | PUNJAB | 24.37 | 5 | 1.63 | 5 | 34.70 | 5 | 8.69 | 4 | 1.80 | 5.0 | 0.53 | 4 |
| RUPNAGAR | PUNJAB | 26.16 | 7 | 1.53 | 4 | 32.86 | 8 | 8.64 | 7 | 2.59 | 8.0 | 0.83 | 7 |
| SAHIBZADA AJIT SINGH NAGAR | PUNJAB | 26.15 | 7 | 1.58 | 4 | 29.12 | 7 | 4.41 | 4 | 2.05 | 7.0 | 0.55 | 4 |
| SANGRUR | PUNJAB | 21.63 | 2 | 1.42 | 2 | 27.74 | 9 | 5.58 | 6 | 2.77 | 9.0 | 0.77 | 6 |
| BARNALA | PUNJAB | 23.15 | 3 | 1.48 | 3 | 28.36 | 6 | 6.21 | 6 | 2.00 | 6.0 | 0.73 | 6 |
| CHANDIGARH | CHANDIGARH | 27.62 | 8 | 1.76 | 6 | 27.96 | 6 | 4.27 | 5 | 1.94 | 6.0 | 0.63 | 5 |
| UTTARKASHI | UTTARAKHAND | 21.00 | 2 | 1.91 | 8 | 25.85 | 10 | 9.52 | 10 | 4.11 | 10.0 | 9.14 | 10 |
| CHAMOLI | UTTARAKHAND | 19.46 | 1 | 1.46 | 3 | 17.16 | 6 | 5.34 | 10 | 1.96 | 6.0 | 3.52 | 10 |
| RUDRAPRAYAG | UTTARAKHAND | 19.59 | 1 | 1.59 | 4 | 15.72 | 4 | 4.36 | 10 | 1.66 | 4.0 | 4.63 | 10 |
| TEHRI GARHWAL | UTTARAKHAND | 20.35 | 1 | 1.44 | 3 | 17.96 | 2 | 6.64 | 2 | 1.30 | 2.0 | 0.39 | 2 |
| DEHRADUN | UTTARAKHAND | 21.56 | 2 | 1.62 | 5 | 19.36 | 6 | 4.75 | 5 | 1.87 | 6.0 | 0.61 | 5 |
| GARHWAL | UTTARAKHAND | 19.98 | 1 | 1.23 | 1 | 16.83 | 3 | 4.53 | 3 | 1.45 | 3.0 | 0.42 | 3 |
| PITHORAGARH | UTTARAKHAND | 17.90 | 1 | 1.35 | 2 | 9.21 | 1 | 2.24 | 1 | 0.86 | 1.0 | 0.23 | 1 |
| BAGESHWAR | UTTARAKHAND | 17.88 | 1 | 1.34 | 2 | 10.17 | 1 | 2.73 | 1 | 0.76 | 1.0 | 0.12 | 1 |
| ALMORA | UTTARAKHAND | 18.85 | 1 | 0.83 | 1 | 14.01 | 1 | 3.95 | 1 | 0.64 | 1.0 | 0.08 | 1 |
| CHAMPAWAT | UTTARAKHAND | 19.31 | 1 | 1.89 | 8 | 10.92 | 1 | 2.62 | 1 | 0.77 | 1.0 | 0.16 | 1 |
| NAINITAL | UTTARAKHAND | 18.72 | 1 | 1.40 | 2 | 14.82 | 3 | 5.10 | 3 | 1.39 | 3.0 | 0.43 | 3 |
| UDHAM SINGH NAGAR | UTTARAKHAND | 23.51 | 4 | 1.66 | 5 | 21.12 | 6 | 4.83 | 5 | 1.95 | 6.0 | 0.64 | 5 |
| HARDWAR | UTTARAKHAND | 19.35 | 1 | 1.34 | 2 | 18.75 | 6 | 4.24 | 6 | 1.86 | 6.0 | 0.79 | 6 |
| PANCHKULA | HARYANA | 25.43 | 6 | 1.34 | 2 | 28.00 | 7 | 5.13 | 6 | 2.25 | 7.0 | 0.68 | 6 |
| AMBALA | HARYANA | 22.50 | 3 | 1.65 | 5 | 22.35 | 7 | 5.56 | 6 | 2.15 | 7.0 | 0.69 | 6 |
| YAMUNANAGAR | HARYANA | 23.44 | 4 | 1.75 | 6 | 30.18 | 8 | 6.10 | 8 | 2.59 | 8.0 | 1.05 | 8 |
| KURUKSHETRA | HARYANA | 25.05 | 6 | 1.67 | 5 | 28.66 | 8 | 5.99 | 8 | 2.54 | 8.0 | 1.14 | 8 |
| KAITHAL | HARYANA | 23.96 | 5 | 1.53 | 4 | 34.57 | 8 | 7.90 | 8 | 2.33 | 8.0 | 1.02 | 8 |
| KARNAL | HARYANA | 24.18 | 5 | 2.11 | 9 | 32.49 | 10 | 6.56 | 9 | 3.55 | 10.0 | 1.30 | 9 |
| PANIPAT | HARYANA | 27.35 | 8 | 2.00 | 9 | 32.07 | 10 | 6.19 | 9 | 3.96 | 10.0 | 1.52 | 9 |
| SONIPAT | HARYANA | 23.38 | 4 | 1.58 | 4 | 26.85 | 10 | 4.53 | 9 | 3.47 | 10.0 | 1.16 | 9 |
| JIND | HARYANA | 23.37 | 4 | 1.53 | 4 | 31.84 | 10 | 6.08 | 10 | 3.50 | 10.0 | 1.54 | 10 |
| FATEHABAD | HARYANA | 24.36 | 5 | 1.58 | 4 | 33.27 | 9 | 5.44 | 7 | 2.76 | 9.0 | 0.89 | 7 |
| SIRSA | HARYANA | 26.30 | 7 | 1.72 | 6 | 27.28 | 10 | 4.47 | 10 | 4.66 | 10.0 | 1.95 | 10 |
| HISAR | HARYANA | 23.63 | 4 | 1.56 | 4 | 32.44 | 10 | 7.38 | 10 | 4.60 | 10.0 | 1.90 | 10 |
| ROHTAK | HARYANA | 23.86 | 4 | 1.58 | 4 | 35.49 | 10 | 7.45 | 10 | 4.11 | 10.0 | 1.81 | 10 |
| JHAJJAR | HARYANA | 24.57 | 5 | 1.61 | 5 | 31.87 | 9 | 6.11 | 8 | 2.77 | 9.0 | 1.08 | 8 |
| MAHENDRAGARH | HARYANA | 22.91 | 3 | 2.00 | 9 | 32.83 | 10 | 6.16 | 10 | 3.75 | 10.0 | 1.72 | 10 |
| REWARI | HARYANA | 24.32 | 5 | 1.56 | 4 | 32.40 | 9 | 6.49 | 10 | 3.29 | 9.0 | 1.72 | 10 |
| GURGAON | HARYANA | 28.52 | 9 | 1.57 | 4 | 33.37 | 6 | 6.25 | 4 | 1.98 | 6.0 | 0.49 | 4 |
| MEWAT | HARYANA | 25.22 | 6 | 2.19 | 10 | 29.86 | 8 | 5.87 | 10 | 2.45 | 8.0 | 1.55 | 10 |
| FARIDABAD | HARYANA | 26.35 | 7 | 1.85 | 7 | 24.54 | 7 | 6.03 | 5 | 2.11 | 7.0 | 0.65 | 5 |
| PALWAL | HARYANA | 26.59 | 7 | 2.13 | 9 | 27.09 | 6 | 6.03 | 6 | 1.91 | 6.0 | 0.76 | 6 |
| GANGANAGAR | RAJASTHAN | 23.71 | 4 | 1.89 | 8 | 30.52 | 8 | 6.00 | 8 | 2.46 | 8.0 | 1.08 | 8 |
| HANUMANGARH | RAJASTHAN | 23.40 | 4 | 1.61 | 5 | 32.18 | 8 | 6.52 | 8 | 2.66 | 8.0 | 1.04 | 8 |
| BIKANER | RAJASTHAN | 24.60 | 5 | 1.74 | 6 | 29.92 | 6 | 8.07 | 7 | 2.00 | 6.0 | 0.81 | 7 |
| CHURU | RAJASTHAN | 21.30 | 2 | 1.63 | 5 | 28.19 | 8 | 6.32 | 8 | 2.38 | 8.0 | 1.10 | 8 |
| JHUNJHUNUN | RAJASTHAN | 22.38 | 3 | 1.73 | 6 | 27.90 | 9 | 6.29 | 10 | 3.15 | 9.0 | 1.64 | 10 |
| ALWAR | RAJASTHAN | 24.17 | 5 | 1.77 | 7 | 24.79 | 8 | 5.91 | 9 | 2.50 | 8.0 | 1.34 | 9 |
| BHARATPUR | RAJASTHAN | 24.01 | 5 | 1.80 | 7 | 31.10 | 9 | 8.02 | 8 | 3.08 | 9.0 | 1.06 | 8 |
| DHAULPUR | RAJASTHAN | 25.11 | 6 | 1.72 | 6 | 37.34 | 9 | 8.51 | 9 | 2.88 | 9.0 | 1.21 | 9 |
| KARAULI | RAJASTHAN | 26.02 | 7 | 1.95 | 8 | 30.57 | 6 | 7.98 | 4 | 1.91 | 6.0 | 0.54 | 4 |
| SAWAI MADHOPUR | RAJASTHAN | 26.18 | 7 | 2.40 | 10 | 26.23 | 7 | 5.75 | 7 | 2.07 | 7.0 | 0.87 | 7 |
| DAUSA | RAJASTHAN | 24.73 | 6 | 2.28 | 10 | 31.17 | 7 | 8.14 | 5 | 2.29 | 7.0 | 0.61 | 5 |
| JAIPUR | RAJASTHAN | 23.35 | 4 | 1.88 | 8 | 26.30 | 7 | 7.67 | 7 | 2.17 | 7.0 | 0.81 | 7 |
| SIKAR | RAJASTHAN | 22.18 | 3 | 1.71 | 6 | 19.93 | 6 | 4.71 | 7 | 1.96 | 6.0 | 0.83 | 7 |
| NAGAUR | RAJASTHAN | 22.64 | 3 | 2.07 | 9 | 20.95 | 3 | 5.62 | 4 | 1.40 | 3.0 | 0.53 | 4 |
| JODHPUR | RAJASTHAN | 21.36 | 2 | 1.65 | 5 | 20.57 | 2 | 6.29 | 2 | 1.29 | 2.0 | 0.39 | 2 |
| JAISALMER | RAJASTHAN | 21.93 | 2 | 1.34 | 2 | 21.43 | 1 | 6.64 | 2 | 0.91 | 1.0 | 0.25 | 2 |
| BARMER | RAJASTHAN | 22.83 | 3 | 2.42 | 10 | 24.12 | 1 | 12.48 | 2 | 0.93 | 1.0 | 0.28 | 2 |
| JALOR | RAJASTHAN | 25.44 | 6 | 2.47 | 10 | 31.61 | 1 | 11.24 | 2 | 0.77 | 1.0 | 0.34 | 2 |
| SIROHI | RAJASTHAN | 25.99 | 7 | 1.94 | 8 | 32.81 | 6 | 9.09 | 8 | 1.88 | 6.0 | 1.03 | 8 |
| PALI | RAJASTHAN | 24.53 | 5 | 1.59 | 4 | 29.62 | 6 | 7.18 | 7 | 2.03 | 6.0 | 0.89 | 7 |
| AJMER | RAJASTHAN | 23.12 | 3 | 1.85 | 7 | 26.98 | 5 | 6.46 | 5 | 1.73 | 5.0 | 0.65 | 5 |
| TONK | RAJASTHAN | 24.06 | 5 | 2.06 | 9 | 25.51 | 6 | 5.58 | 7 | 1.90 | 6.0 | 0.85 | 7 |
| BUNDI | RAJASTHAN | 24.84 | 6 | 1.79 | 7 | 27.41 | 6 | 5.71 | 4 | 1.88 | 6.0 | 0.58 | 4 |
| BHILWARA | RAJASTHAN | 24.87 | 6 | 1.56 | 4 | 22.32 | 3 | 4.44 | 2 | 1.31 | 3.0 | 0.32 | 2 |
| RAJSAMAND | RAJASTHAN | 24.95 | 6 | 1.62 | 5 | 29.47 | 4 | 7.53 | 3 | 1.57 | 4.0 | 0.46 | 3 |
| DUNGARPUR | RAJASTHAN | 27.82 | 8 | 2.46 | 10 | 39.95 | 3 | 7.93 | 3 | 1.36 | 3.0 | 0.43 | 3 |
| BANSWARA | RAJASTHAN | 24.84 | 6 | 1.80 | 7 | 25.33 | 2 | 8.31 | 3 | 1.23 | 2.0 | 0.45 | 3 |
| CHITTAURGARH | RAJASTHAN | 22.95 | 3 | 1.55 | 4 | 20.17 | 7 | 4.46 | 7 | 2.09 | 7.0 | 0.88 | 7 |
| KOTA | RAJASTHAN | 26.16 | 7 | 1.80 | 7 | 22.15 | 3 | 5.94 | 4 | 1.33 | 3.0 | 0.55 | 4 |
| BARAN | RAJASTHAN | 27.76 | 8 | 2.03 | 9 | 27.72 | 3 | 7.08 | 4 | 1.31 | 3.0 | 0.54 | 4 |
| JHALAWAR | RAJASTHAN | 23.60 | 4 | 1.91 | 8 | 25.23 | 3 | 7.68 | 3 | 1.33 | 3.0 | 0.44 | 3 |
| UDAIPUR | RAJASTHAN | 24.67 | 5 | 1.81 | 7 | 32.67 | 2 | 9.34 | 2 | 1.10 | 2.0 | 0.37 | 2 |
| PRATAPGARH | RAJASTHAN | 25.09 | 6 | 1.79 | 7 | 25.60 | 2 | 7.92 | 3 | 1.16 | 2.0 | 0.44 | 3 |
| SAHARANPUR | UTTAR PRADESH | 21.05 | 2 | 1.31 | 2 | 19.54 | 2 | 6.11 | 2 | 1.13 | 2.0 | 0.33 | 2 |
| BIJNOR | UTTAR PRADESH | 23.46 | 4 | 1.77 | 7 | 21.09 | 2 | 4.53 | 3 | 1.25 | 2.0 | 0.44 | 3 |
| RAMPUR | UTTAR PRADESH | 24.57 | 5 | 1.42 | 2 | 25.85 | 4 | 8.14 | 4 | 1.67 | 4.0 | 0.50 | 4 |
| JYOTIBA PHULE NAGAR | UTTAR PRADESH | 23.14 | 3 | 1.55 | 4 | 21.98 | 3 | 5.45 | 7 | 1.31 | 3.0 | 0.84 | 7 |
| MEERUT | UTTAR PRADESH | 21.89 | 2 | 1.54 | 4 | 21.35 | 7 | 4.63 | 8 | 2.30 | 7.0 | 1.14 | 8 |
| BAGHPAT | UTTAR PRADESH | 24.59 | 5 | 1.66 | 5 | 24.47 | 7 | 5.05 | 6 | 2.11 | 7.0 | 0.80 | 6 |
| GAUTAM BUDDHA NAGAR | UTTAR PRADESH | 27.36 | 8 | 1.80 | 7 | 26.22 | 4 | 4.95 | 3 | 1.61 | 4.0 | 0.43 | 3 |
| BULANDSHAHR | UTTAR PRADESH | 22.66 | 3 | 1.49 | 3 | 26.05 | 6 | 6.26 | 6 | 1.94 | 6.0 | 0.75 | 6 |
| ALIGARH | UTTAR PRADESH | 22.93 | 3 | 1.46 | 3 | 29.59 | 4 | 5.40 | 5 | 1.65 | 4.0 | 0.64 | 5 |
| MAHAMAYA NAGAR | UTTAR PRADESH | 23.47 | 4 | 1.79 | 7 | 29.33 | 9 | 6.32 | 9 | 2.80 | 9.0 | 1.22 | 9 |
| MATHURA | UTTAR PRADESH | 25.81 | 7 | 1.49 | 3 | 29.94 | 9 | 7.04 | 10 | 2.99 | 9.0 | 2.07 | 10 |
| AGRA | UTTAR PRADESH | 23.46 | 4 | 1.58 | 4 | 33.15 | 8 | 6.12 | 7 | 2.64 | 8.0 | 0.93 | 7 |
| FIROZABAD | UTTAR PRADESH | 24.10 | 5 | 1.68 | 5 | 28.02 | 6 | 5.96 | 8 | 2.03 | 6.0 | 0.98 | 8 |
| MAINPURI | UTTAR PRADESH | 24.56 | 5 | 1.66 | 5 | 28.14 | 7 | 6.02 | 7 | 2.21 | 7.0 | 0.86 | 7 |
| BAREILLY | UTTAR PRADESH | 28.07 | 8 | 1.82 | 7 | 26.49 | 3 | 5.87 | 4 | 1.37 | 3.0 | 0.50 | 4 |
| PILIBHIT | UTTAR PRADESH | 24.03 | 5 | 1.31 | 2 | 23.95 | 6 | 5.70 | 5 | 1.91 | 6.0 | 0.60 | 5 |
| SHAHJAHANPUR | UTTAR PRADESH | 25.68 | 6 | 1.92 | 8 | 29.33 | 6 | 5.70 | 6 | 1.98 | 6.0 | 0.70 | 6 |
| KHERI | UTTAR PRADESH | 24.17 | 5 | 1.73 | 6 | 24.31 | 8 | 4.48 | 9 | 2.38 | 8.0 | 1.34 | 9 |
| SITAPUR | UTTAR PRADESH | 25.82 | 7 | 1.70 | 6 | 24.24 | 8 | 4.85 | 7 | 2.48 | 8.0 | 0.88 | 7 |
| HARDOI | UTTAR PRADESH | 25.01 | 6 | 1.93 | 8 | 29.74 | 6 | 6.20 | 6 | 1.95 | 6.0 | 0.78 | 6 |
| UNNAO | UTTAR PRADESH | 22.81 | 3 | 1.55 | 4 | 23.64 | 5 | 5.16 | 5 | 1.75 | 5.0 | 0.67 | 5 |
| LUCKNOW | UTTAR PRADESH | 26.42 | 7 | 1.46 | 3 | 23.24 | 5 | 4.73 | 5 | 1.79 | 5.0 | 0.65 | 5 |
| FARRUKHABAD | UTTAR PRADESH | 25.13 | 6 | 1.64 | 5 | 27.94 | 4 | 6.16 | 3 | 1.48 | 4.0 | 0.46 | 3 |
| KANNAUJ | UTTAR PRADESH | 26.18 | 7 | 1.81 | 7 | 32.39 | 8 | 7.30 | 9 | 2.59 | 8.0 | 1.27 | 9 |
| ETAWAH | UTTAR PRADESH | 26.65 | 7 | 2.34 | 10 | 25.66 | 5 | 6.04 | 6 | 1.77 | 5.0 | 0.72 | 6 |
| AURAIYA | UTTAR PRADESH | 21.95 | 2 | 1.80 | 7 | 15.94 | 2 | 4.41 | 3 | 1.28 | 2.0 | 0.46 | 3 |
| KANPUR DEHAT | UTTAR PRADESH | 25.49 | 6 | 2.13 | 9 | 25.39 | 5 | 5.39 | 5 | 1.80 | 5.0 | 0.62 | 5 |
| KANPUR NAGAR | UTTAR PRADESH | 26.06 | 7 | 2.07 | 9 | 25.80 | 7 | 6.32 | 8 | 2.13 | 7.0 | 0.96 | 8 |
| JALAUN | UTTAR PRADESH | 21.71 | 2 | 1.45 | 3 | 19.12 | 7 | 5.99 | 8 | 2.15 | 7.0 | 1.05 | 8 |
| JHANSI | UTTAR PRADESH | 23.70 | 4 | 1.56 | 4 | 16.10 | 3 | 4.38 | 4 | 1.32 | 3.0 | 0.54 | 4 |
| LALITPUR | UTTAR PRADESH | 21.53 | 2 | 1.72 | 6 | 16.10 | 2 | 3.88 | 2 | 1.17 | 2.0 | 0.39 | 2 |
| HAMIRPUR | UTTAR PRADESH | 24.62 | 5 | 1.99 | 9 | 18.70 | 4 | 4.54 | 7 | 1.62 | 4.0 | 0.81 | 7 |
| MAHOBA | UTTAR PRADESH | 26.17 | 7 | 1.94 | 8 | 19.92 | 2 | 5.04 | 2 | 1.22 | 2.0 | 0.39 | 2 |
| BANDA | UTTAR PRADESH | 23.91 | 5 | 1.85 | 7 | 24.94 | 3 | 7.04 | 7 | 1.40 | 3.0 | 0.89 | 7 |
| CHITRAKOOT | UTTAR PRADESH | 23.18 | 3 | 1.86 | 8 | 21.86 | 3 | 4.20 | 3 | 1.37 | 3.0 | 0.47 | 3 |
| FATEHPUR | UTTAR PRADESH | 25.21 | 6 | 2.05 | 9 | 31.53 | 8 | 4.73 | 9 | 2.71 | 8.0 | 1.35 | 9 |
| PRATAPGARH | UTTAR PRADESH | 26.72 | 8 | 1.63 | 5 | 18.53 | 1 | 4.87 | 1 | 0.84 | 1.0 | 0.20 | 1 |
| KAUSHAMBI | UTTAR PRADESH | 28.08 | 8 | 1.71 | 6 | 21.35 | 4 | 4.39 | 2 | 1.57 | 4.0 | 0.39 | 2 |
| ALLAHABAD | UTTAR PRADESH | 24.58 | 5 | 1.53 | 4 | 18.55 | 3 | 3.58 | 3 | 1.39 | 3.0 | 0.43 | 3 |
| BARA BANKI | UTTAR PRADESH | 22.96 | 3 | 1.42 | 2 | 26.10 | 9 | 6.39 | 9 | 3.12 | 9.0 | 1.35 | 9 |
| FAIZABAD | UTTAR PRADESH | 26.70 | 8 | 1.83 | 7 | 21.09 | 5 | 4.59 | 7 | 1.78 | 5.0 | 0.90 | 7 |
| AMBEDKAR NAGAR | UTTAR PRADESH | 25.35 | 6 | 1.64 | 5 | 23.01 | 4 | 6.02 | 4 | 1.51 | 4.0 | 0.53 | 4 |
| BAHRAICH | UTTAR PRADESH | 22.66 | 3 | 1.79 | 7 | 23.75 | 4 | 4.93 | 5 | 1.56 | 4.0 | 0.63 | 5 |
| SHRAWASTI | UTTAR PRADESH | 23.65 | 4 | 1.41 | 2 | 17.73 | 7 | 4.25 | 6 | 2.09 | 7.0 | 0.80 | 6 |
| BALRAMPUR | UTTAR PRADESH | 25.18 | 6 | 1.62 | 5 | 24.41 | 5 | 5.72 | 5 | 1.70 | 5.0 | 0.63 | 5 |
| GONDA | UTTAR PRADESH | 22.51 | 3 | 1.79 | 7 | 23.46 | 6 | 4.60 | 8 | 2.02 | 6.0 | 1.14 | 8 |
| SIDDHARTHNAGAR | UTTAR PRADESH | 25.01 | 6 | 1.97 | 9 | 22.90 | 4 | 6.39 | 5 | 1.58 | 4.0 | 0.67 | 5 |
| BASTI | UTTAR PRADESH | 22.52 | 3 | 1.63 | 5 | 16.78 | 2 | 4.55 | 2 | 1.20 | 2.0 | 0.30 | 2 |
| SANT KABIR NAGAR | UTTAR PRADESH | 23.10 | 3 | 1.86 | 8 | 18.12 | 4 | 4.80 | 5 | 1.49 | 4.0 | 0.64 | 5 |
| MAHRAJGANJ | UTTAR PRADESH | 26.71 | 8 | 2.10 | 9 | 17.78 | 2 | 4.73 | 2 | 1.05 | 2.0 | 0.30 | 2 |
| GORAKHPUR | UTTAR PRADESH | 25.89 | 7 | 2.04 | 9 | 22.86 | 2 | 5.98 | 4 | 1.27 | 2.0 | 0.51 | 4 |
| KUSHINAGAR | UTTAR PRADESH | 23.26 | 4 | 1.81 | 7 | 17.91 | 2 | 4.34 | 2 | 1.08 | 2.0 | 0.33 | 2 |
| DEORIA | UTTAR PRADESH | 20.75 | 2 | 1.88 | 8 | 13.83 | 1 | 4.41 | 1 | 0.75 | 1.0 | 0.19 | 1 |
| AZAMGARH | UTTAR PRADESH | 25.68 | 6 | 1.78 | 7 | 24.41 | 3 | 5.96 | 3 | 1.47 | 3.0 | 0.41 | 3 |
| MAU | UTTAR PRADESH | 23.84 | 4 | 1.70 | 6 | 18.71 | 1 | 5.49 | 1 | 0.77 | 1.0 | 0.14 | 1 |
| BALLIA | UTTAR PRADESH | 23.81 | 4 | 2.08 | 9 | 25.06 | 2 | 8.51 | 2 | 1.12 | 2.0 | 0.33 | 2 |
| JAUNPUR | UTTAR PRADESH | 19.56 | 1 | 1.61 | 5 | 14.61 | 1 | 4.55 | 1 | 0.79 | 1.0 | 0.22 | 1 |
| GHAZIPUR | UTTAR PRADESH | 24.47 | 5 | 1.79 | 7 | 19.89 | 1 | 5.69 | 1 | 0.73 | 1.0 | 0.13 | 1 |
| CHANDAULI | UTTAR PRADESH | 23.61 | 4 | 1.55 | 4 | 21.74 | 3 | 5.32 | 4 | 1.31 | 3.0 | 0.50 | 4 |
| VARANASI | UTTAR PRADESH | 22.09 | 2 | 1.96 | 8 | 15.29 | 3 | 4.39 | 3 | 1.31 | 3.0 | 0.42 | 3 |
| SANT RAVIDAS NAGAR (BHADOHI) | UTTAR PRADESH | 23.45 | 4 | 1.83 | 7 | 17.87 | 2 | 5.16 | 4 | 1.21 | 2.0 | 0.55 | 4 |
| MIRZAPUR | UTTAR PRADESH | 23.66 | 4 | 1.72 | 6 | 17.30 | 2 | 3.67 | 2 | 1.22 | 2.0 | 0.36 | 2 |
| SONBHADRA | UTTAR PRADESH | 25.79 | 7 | 1.89 | 8 | 15.80 | 3 | 4.49 | 2 | 1.36 | 3.0 | 0.38 | 2 |
| ETAH | UTTAR PRADESH | 24.64 | 5 | 1.83 | 7 | 27.66 | 4 | 6.61 | 4 | 1.64 | 4.0 | 0.53 | 4 |
| KANSHIRAM NAGAR | UTTAR PRADESH | 23.31 | 4 | 1.60 | 4 | 34.78 | 9 | 7.80 | 9 | 2.98 | 9.0 | 1.15 | 9 |
| PASHCHIM CHAMPARAN | BIHAR | 26.63 | 7 | 1.74 | 6 | 22.82 | 5 | 4.93 | 6 | 1.79 | 5.0 | 0.73 | 6 |
| PURBA CHAMPARAN | BIHAR | 28.71 | 9 | 1.70 | 6 | 27.35 | 4 | 7.08 | 3 | 1.56 | 4.0 | 0.41 | 3 |
| SHEOHAR | BIHAR | 31.32 | 10 | 2.36 | 10 | 27.09 | 2 | 6.90 | 3 | 1.28 | 2.0 | 0.40 | 3 |
| SITAMARHI | BIHAR | 29.47 | 10 | 2.07 | 9 | 29.82 | 4 | 6.70 | 3 | 1.49 | 4.0 | 0.43 | 3 |
| MADHUBANI | BIHAR | 28.85 | 9 | 1.79 | 7 | 29.63 | 6 | 5.56 | 5 | 1.92 | 6.0 | 0.62 | 5 |
| SUPAUL | BIHAR | 29.60 | 10 | 1.84 | 7 | 28.99 | 3 | 5.43 | 4 | 1.42 | 3.0 | 0.57 | 4 |
| ARARIA | BIHAR | 29.92 | 10 | 2.21 | 10 | 34.70 | 4 | 7.01 | 6 | 1.61 | 4.0 | 0.68 | 6 |
| KISHANGANJ | BIHAR | 29.83 | 10 | 1.76 | 6 | 31.52 | 7 | 7.43 | 7 | 2.19 | 7.0 | 0.94 | 7 |
| PURNIA | BIHAR | 28.91 | 9 | 2.21 | 10 | 33.13 | 7 | 6.25 | 7 | 2.25 | 7.0 | 0.85 | 7 |
| KATIHAR | BIHAR | 29.05 | 9 | 1.82 | 7 | 35.79 | 4 | 6.73 | 4 | 1.54 | 4.0 | 0.58 | 4 |
| MADHEPURA | BIHAR | 30.64 | 10 | 2.06 | 9 | 30.67 | 7 | 6.73 | 7 | 2.20 | 7.0 | 0.87 | 7 |
| SAHARSA | BIHAR | 29.11 | 9 | 1.58 | 4 | 32.50 | 5 | 6.33 | 4 | 1.80 | 5.0 | 0.57 | 4 |
| DARBHANGA | BIHAR | 27.82 | 8 | 1.42 | 2 | 30.13 | 5 | 5.81 | 8 | 1.79 | 5.0 | 1.05 | 8 |
| MUZAFFARPUR | BIHAR | 29.23 | 9 | 1.55 | 4 | 25.89 | 4 | 6.00 | 4 | 1.63 | 4.0 | 0.54 | 4 |
| GOPALGANJ | BIHAR | 26.31 | 7 | 1.91 | 8 | 24.94 | 3 | 7.43 | 2 | 1.42 | 3.0 | 0.36 | 2 |
| SIWAN | BIHAR | 28.61 | 9 | 1.80 | 7 | 22.02 | 7 | 4.68 | 8 | 2.11 | 7.0 | 1.11 | 8 |
| SARAN | BIHAR | 29.54 | 10 | 2.05 | 9 | 28.72 | 6 | 5.21 | 5 | 1.96 | 6.0 | 0.66 | 5 |
| VAISHALI | BIHAR | 28.01 | 8 | 1.80 | 7 | 32.55 | 4 | 7.90 | 4 | 1.61 | 4.0 | 0.49 | 4 |
| SAMASTIPUR | BIHAR | 29.77 | 10 | 1.56 | 4 | 27.98 | 3 | 6.09 | 7 | 1.40 | 3.0 | 0.95 | 7 |
| BEGUSARAI | BIHAR | 28.37 | 9 | 1.93 | 8 | 30.78 | 5 | 7.33 | 5 | 1.74 | 5.0 | 0.61 | 5 |
| KHAGARIA | BIHAR | 30.30 | 10 | 2.04 | 9 | 25.92 | 4 | 6.28 | 4 | 1.53 | 4.0 | 0.49 | 4 |
| BHAGALPUR | BIHAR | 29.95 | 10 | 2.63 | 10 | 38.04 | 5 | 7.54 | 6 | 1.80 | 5.0 | 0.79 | 6 |
| BANKA | BIHAR | 28.54 | 9 | 1.93 | 8 | 33.37 | 8 | 6.94 | 6 | 2.37 | 8.0 | 0.80 | 6 |
| MUNGER | BIHAR | 30.07 | 10 | 2.12 | 9 | 38.25 | 5 | 5.89 | 4 | 1.81 | 5.0 | 0.50 | 4 |
| LAKHISARAI | BIHAR | 29.54 | 10 | 2.16 | 10 | 39.36 | 5 | 6.03 | 7 | 1.83 | 5.0 | 0.95 | 7 |
| SHEIKHPURA | BIHAR | 29.11 | 9 | 1.78 | 7 | 37.29 | 4 | 6.14 | 3 | 1.53 | 4.0 | 0.46 | 3 |
| NALANDA | BIHAR | 31.55 | 10 | 2.23 | 10 | 35.58 | 4 | 6.08 | 3 | 1.48 | 4.0 | 0.48 | 3 |
| PATNA | BIHAR | 31.59 | 10 | 1.50 | 3 | 32.10 | 3 | 4.80 | 2 | 1.35 | 3.0 | 0.33 | 2 |
| BHOJPUR | BIHAR | 29.12 | 9 | 2.28 | 10 | 41.65 | 5 | 7.69 | 5 | 1.74 | 5.0 | 0.59 | 5 |
| BUXAR | BIHAR | 30.74 | 10 | 2.21 | 10 | 32.38 | 4 | 7.71 | 4 | 1.63 | 4.0 | 0.52 | 4 |
| KAIMUR (BHABUA) | BIHAR | 33.23 | 10 | 2.79 | 10 | 32.51 | 3 | 6.38 | 3 | 1.31 | 3.0 | 0.41 | 3 |
| ROHTAS | BIHAR | 30.74 | 10 | 2.69 | 10 | 32.10 | 2 | 7.33 | 2 | 1.20 | 2.0 | 0.28 | 2 |
| AURANGABAD | BIHAR | 30.28 | 10 | 1.72 | 6 | 28.04 | 2 | 6.62 | 3 | 1.18 | 2.0 | 0.41 | 3 |
| GAYA | BIHAR | 30.13 | 10 | 2.65 | 10 | 30.78 | 3 | 7.39 | 4 | 1.47 | 3.0 | 0.50 | 4 |
| NAWADA | BIHAR | 29.25 | 9 | 1.69 | 5 | 39.35 | 3 | 6.72 | 5 | 1.42 | 3.0 | 0.65 | 5 |
| JAMUI | BIHAR | 31.28 | 10 | 2.23 | 10 | 38.19 | 8 | 7.95 | 7 | 2.34 | 8.0 | 0.85 | 7 |
| JEHANABAD | BIHAR | 32.65 | 10 | 2.17 | 10 | 31.12 | 3 | 5.75 | 3 | 1.38 | 3.0 | 0.43 | 3 |
| ARWAL | BIHAR | 30.84 | 10 | 2.13 | 9 | 32.82 | 3 | 6.79 | 4 | 1.37 | 3.0 | 0.56 | 4 |
| NORTH DISTRICT | SIKKIM | 19.66 | 1 | 1.24 | 1 | 15.86 | 4 | 4.10 | 3 | 1.48 | 4.0 | 0.41 | 3 |
| WEST DISTRICT | SIKKIM | 19.67 | 1 | 1.72 | 6 | 19.27 | 2 | 6.50 | 4 | 1.11 | 2.0 | 0.55 | 4 |
| SOUTH DISTRICT | SIKKIM | 19.46 | 1 | 1.34 | 2 | 16.74 | 2 | 4.40 | 2 | 1.02 | 2.0 | 0.26 | 2 |
| EAST DISTRICT | SIKKIM | 19.93 | 1 | 1.28 | 1 | 18.86 | 8 | 3.71 | 7 | 2.34 | 8.0 | 0.90 | 7 |
| TAWANG | ARUNACHAL PRADESH | 19.47 | 1 | 1.30 | 1 | 28.19 | 6 | 13.39 | 9 | 1.89 | 6.0 | 1.30 | 9 |
| WEST KAMENG | ARUNACHAL PRADESH | 21.41 | 2 | 2.30 | 10 | 11.39 | 2 | 3.26 | 4 | 0.96 | 2.0 | 0.49 | 4 |
| EAST KAMENG | ARUNACHAL PRADESH | 18.81 | 1 | 1.09 | 1 | 7.96 | 1 | 1.94 | 1 | 0.49 | 1.0 | 0.06 | 1 |
| PAPUM PARE | ARUNACHAL PRADESH | 22.03 | 2 | 1.61 | 5 | 13.72 | 1 | 3.47 | 1 | 0.85 | 1.0 | 0.20 | 1 |
| UPPER SUBANSIRI | ARUNACHAL PRADESH | 17.11 | 1 | 1.51 | 3 | 16.93 | 2 | 11.53 | 10 | 1.10 | 2.0 | 1.75 | 10 |
| UPPER SIANG | ARUNACHAL PRADESH | 18.23 | 1 | 1.15 | 1 | 10.67 | 1 | 3.74 | 1 | 0.79 | 1.0 | 0.20 | 1 |
| CHANGLANG | ARUNACHAL PRADESH | 28.72 | 9 | 2.16 | 10 | 20.51 | 1 | 5.31 | 2 | 0.93 | 1.0 | 0.33 | 2 |
| LOWER SUBANSIRI | ARUNACHAL PRADESH | 20.89 | 2 | 2.17 | 10 | 12.77 | 1 | 5.63 | 1 | 0.76 | 1.0 | 0.15 | 1 |
| DIBANG VALLEY | ARUNACHAL PRADESH | 19.90 | 1 | 1.36 | 2 | 34.85 | 6 | 15.57 | 8 | 1.91 | 6.0 | 1.14 | 8 |
| LOWER DIBANG VALLEY | ARUNACHAL PRADESH | 20.50 | 2 | 1.34 | 2 | 11.08 | 2 | 2.92 | 4 | 1.06 | 2.0 | 0.55 | 4 |
| ANJAW | ARUNACHAL PRADESH | 18.92 | 1 | 1.43 | 3 | 14.88 | 1 | 7.79 | 2 | 0.76 | 1.0 | 0.31 | 2 |
| MON | NAGALAND | 18.13 | 1 | 1.35 | 2 | 10.04 | 1 | 3.67 | 1 | 0.49 | 1.0 | 0.12 | 1 |
| MOKOKCHUNG | NAGALAND | 15.99 | 1 | 1.39 | 2 | 8.34 | 1 | 2.40 | 1 | 0.68 | 1.0 | 0.21 | 1 |
| ZUNHEBOTO | NAGALAND | 18.73 | 1 | 1.22 | 1 | 9.78 | 1 | 2.64 | 2 | 0.93 | 1.0 | 0.39 | 2 |
| WOKHA | NAGALAND | 18.10 | 1 | 1.16 | 1 | 10.39 | 1 | 2.27 | 1 | 0.38 | 1.0 | 0.03 | 1 |
| DIMAPUR | NAGALAND | 19.20 | 1 | 1.97 | 9 | 10.63 | 1 | 3.23 | 1 | 0.71 | 1.0 | 0.22 | 1 |
| PHEK | NAGALAND | 17.83 | 1 | 1.70 | 6 | 11.17 | 1 | 2.91 | 1 | 0.35 | 1.0 | 0.03 | 1 |
| TUENSANG | NAGALAND | 17.53 | 1 | 1.37 | 2 | 9.67 | 1 | 2.17 | 1 | 0.45 | 1.0 | 0.07 | 1 |
| LONGLENG | NAGALAND | 20.41 | 1 | 1.28 | 1 | 11.64 | 1 | 2.71 | 1 | 0.79 | 1.0 | 0.22 | 1 |
| KIPHIRE | NAGALAND | 20.42 | 2 | 1.55 | 4 | 9.50 | 1 | 2.44 | 1 | 0.59 | 1.0 | 0.11 | 1 |
| KOHIMA | NAGALAND | 13.93 | 1 | 0.86 | 1 | 5.39 | 1 | 1.15 | 1 | 0.34 | 1.0 | 0.01 | 1 |
| PEREN | NAGALAND | 17.77 | 1 | 1.43 | 3 | 9.73 | 1 | 3.48 | 1 | 0.74 | 1.0 | 0.20 | 1 |
| SENAPATI | MANIPUR | 18.45 | 1 | 1.27 | 1 | 8.34 | 1 | 2.32 | 1 | 0.46 | 1.0 | 0.07 | 1 |
| TAMENGLONG | MANIPUR | 17.46 | 1 | 1.38 | 2 | 8.12 | 1 | 2.08 | 1 | 0.53 | 1.0 | 0.09 | 1 |
| CHURACHANDPUR | MANIPUR | 18.48 | 1 | 0.79 | 1 | 10.06 | 1 | 2.74 | 1 | 0.61 | 1.0 | 0.12 | 1 |
| BISHNUPUR | MANIPUR | 20.13 | 1 | 1.50 | 3 | 10.63 | 1 | 1.82 | 2 | 0.90 | 1.0 | 0.35 | 2 |
| THOUBAL | MANIPUR | 16.98 | 1 | 1.33 | 2 | 7.25 | 1 | 2.32 | 1 | 0.40 | 1.0 | 0.06 | 1 |
| IMPHAL WEST | MANIPUR | 17.80 | 1 | 1.26 | 1 | 7.87 | 1 | 1.50 | 2 | 0.75 | 1.0 | 0.28 | 2 |
| IMPHAL EAST | MANIPUR | 20.09 | 1 | 1.48 | 3 | 11.47 | 1 | 2.96 | 2 | 0.87 | 1.0 | 0.26 | 2 |
| UKHRUL | MANIPUR | 16.04 | 1 | 0.97 | 1 | 7.72 | 1 | 1.68 | 1 | 0.40 | 1.0 | 0.03 | 1 |
| CHANDEL | MANIPUR | 16.55 | 1 | 1.08 | 1 | 9.22 | 1 | 3.48 | 1 | 0.56 | 1.0 | 0.13 | 1 |
| MAMIT | MIZORAM | 21.89 | 2 | 1.59 | 4 | 11.82 | 1 | 3.68 | 1 | 0.82 | 1.0 | 0.24 | 1 |
| KOLASIB | MIZORAM | 20.12 | 1 | 1.45 | 3 | 15.07 | 2 | 4.41 | 1 | 1.07 | 2.0 | 0.24 | 1 |
| AIZAWL | MIZORAM | 18.87 | 1 | 1.26 | 1 | 11.67 | 2 | 3.04 | 2 | 1.11 | 2.0 | 0.28 | 2 |
| CHAMPHAI | MIZORAM | 18.10 | 1 | 1.35 | 2 | 9.25 | 1 | 2.17 | 1 | 0.52 | 1.0 | 0.05 | 1 |
| SERCHHIP | MIZORAM | 18.20 | 1 | 1.30 | 1 | 13.48 | 1 | 3.30 | 1 | 0.85 | 1.0 | 0.24 | 1 |
| LUNGLEI | MIZORAM | 19.18 | 1 | 1.55 | 4 | 13.61 | 1 | 4.18 | 2 | 0.93 | 1.0 | 0.27 | 2 |
| LAWNGTLAI | MIZORAM | 23.09 | 3 | 2.15 | 10 | 19.48 | 3 | 6.20 | 3 | 1.31 | 3.0 | 0.41 | 3 |
| SAIHA | MIZORAM | 19.68 | 1 | 1.51 | 3 | 18.98 | 5 | 5.14 | 6 | 1.68 | 5.0 | 0.71 | 6 |
| DHALAI | TRIPURA | 30.03 | 10 | 1.91 | 8 | 36.33 | 7 | 8.00 | 6 | 2.12 | 7.0 | 0.68 | 6 |
| SOUTH GARO HILLS | MEGHALAYA | 25.59 | 6 | 1.98 | 9 | 28.23 | 1 | 6.45 | 1 | 0.86 | 1.0 | 0.22 | 1 |
| RIBHOI | MEGHALAYA | 23.49 | 4 | 1.54 | 4 | 33.97 | 8 | 10.13 | 8 | 2.31 | 8.0 | 1.10 | 8 |
| EAST KHASI HILLS | MEGHALAYA | 21.54 | 2 | 0.94 | 1 | 24.02 | 8 | 5.62 | 9 | 2.31 | 8.0 | 1.19 | 9 |
| KOKRAJHAR | ASSAM | 26.81 | 8 | 1.77 | 7 | 31.06 | 3 | 8.72 | 2 | 1.36 | 3.0 | 0.34 | 2 |
| GOALPARA | ASSAM | 29.30 | 9 | 2.15 | 10 | 32.65 | 4 | 6.43 | 4 | 1.51 | 4.0 | 0.58 | 4 |
| BARPETA | ASSAM | 31.33 | 10 | 2.61 | 10 | 30.36 | 3 | 7.47 | 3 | 1.47 | 3.0 | 0.48 | 3 |
| MORIGAON | ASSAM | 30.74 | 10 | 2.36 | 10 | 30.35 | 4 | 6.37 | 4 | 1.61 | 4.0 | 0.49 | 4 |
| LAKHIMPUR | ASSAM | 31.26 | 10 | 2.05 | 9 | 30.80 | 3 | 6.96 | 3 | 1.39 | 3.0 | 0.43 | 3 |
| DHEMAJI | ASSAM | 28.90 | 9 | 1.85 | 7 | 31.10 | 6 | 6.62 | 6 | 2.03 | 6.0 | 0.71 | 6 |
| TINSUKIA | ASSAM | 27.80 | 8 | 1.83 | 7 | 40.52 | 7 | 6.86 | 7 | 2.14 | 7.0 | 0.92 | 7 |
| DIBRUGARH | ASSAM | 30.48 | 10 | 1.90 | 8 | 35.85 | 7 | 6.89 | 6 | 2.14 | 7.0 | 0.76 | 6 |
| GOLAGHAT | ASSAM | 29.37 | 9 | 2.09 | 9 | 42.99 | 7 | 9.67 | 7 | 2.04 | 7.0 | 0.92 | 7 |
| DIMA HASAO | ASSAM | 29.31 | 9 | 2.50 | 10 | 29.10 | 2 | 8.71 | 2 | 1.20 | 2.0 | 0.35 | 2 |
| CACHAR | ASSAM | 27.38 | 8 | 2.25 | 10 | 28.80 | 2 | 7.89 | 1 | 1.04 | 2.0 | 0.24 | 1 |
| KARIMGANJ | ASSAM | 26.96 | 8 | 1.66 | 5 | 24.06 | 2 | 5.46 | 3 | 1.29 | 2.0 | 0.43 | 3 |
| HAILAKANDI | ASSAM | 27.64 | 8 | 1.85 | 7 | 31.25 | 6 | 6.40 | 6 | 1.84 | 6.0 | 0.71 | 6 |
| BONGAIGAON | ASSAM | 28.36 | 9 | 2.19 | 10 | 38.06 | 7 | 8.41 | 8 | 2.09 | 7.0 | 1.09 | 8 |
| CHIRANG | ASSAM | 25.86 | 7 | 1.52 | 4 | 37.42 | 5 | 9.95 | 5 | 1.79 | 5.0 | 0.67 | 5 |
| KAMRUP | ASSAM | 28.26 | 9 | 2.02 | 9 | 38.69 | 7 | 8.09 | 9 | 2.26 | 7.0 | 1.27 | 9 |
| KAMRUP METROPOLITAN | ASSAM | 28.16 | 8 | 1.99 | 9 | 40.24 | 9 | 7.02 | 8 | 2.87 | 9.0 | 1.10 | 8 |
| NALBARI | ASSAM | 29.93 | 10 | 1.40 | 2 | 31.99 | 6 | 6.34 | 10 | 1.97 | 6.0 | 2.43 | 10 |
| BAKSA | ASSAM | 27.93 | 8 | 2.00 | 9 | 42.12 | 4 | 10.75 | 5 | 1.57 | 4.0 | 0.59 | 5 |
| DARRANG | ASSAM | 31.52 | 10 | 2.07 | 9 | 34.79 | 4 | 6.89 | 6 | 1.67 | 4.0 | 0.75 | 6 |
| UDALGURI | ASSAM | 25.67 | 6 | 1.87 | 8 | 52.17 | 6 | 8.77 | 7 | 1.91 | 6.0 | 0.91 | 7 |
| DARJILING | WEST BENGAL | 26.73 | 8 | 2.84 | 10 | 29.91 | 4 | 9.13 | 2 | 1.54 | 4.0 | 0.39 | 2 |
| JALPAIGURI | WEST BENGAL | 28.23 | 9 | 1.98 | 9 | 40.70 | 7 | 8.51 | 8 | 2.26 | 7.0 | 0.97 | 8 |
| KOCH BIHAR | WEST BENGAL | 30.36 | 10 | 1.84 | 7 | 39.82 | 8 | 7.91 | 7 | 2.37 | 8.0 | 0.93 | 7 |
| UTTAR DINAJPUR | WEST BENGAL | 28.68 | 9 | 2.29 | 10 | 40.97 | 3 | 9.63 | 4 | 1.44 | 3.0 | 0.51 | 4 |
| DAKSHIN DINAJPUR | WEST BENGAL | 28.37 | 9 | 1.89 | 8 | 49.39 | 8 | 7.22 | 9 | 2.71 | 8.0 | 1.32 | 9 |
| MALDAH | WEST BENGAL | 28.81 | 9 | 1.85 | 7 | 41.03 | 7 | 7.78 | 8 | 2.17 | 7.0 | 1.04 | 8 |
| MURSHIDABAD | WEST BENGAL | 32.18 | 10 | 1.89 | 8 | 41.11 | 5 | 8.71 | 5 | 1.83 | 5.0 | 0.66 | 5 |
| BIRBHUM | WEST BENGAL | 30.66 | 10 | 2.51 | 10 | 43.61 | 4 | 11.45 | 5 | 1.56 | 4.0 | 0.63 | 5 |
| NADIA | WEST BENGAL | 30.27 | 10 | 2.26 | 10 | 40.05 | 5 | 7.45 | 4 | 1.72 | 5.0 | 0.58 | 4 |
| NORTH TWENTY FOUR PARGANAS | WEST BENGAL | 30.64 | 10 | 1.96 | 8 | 32.52 | 1 | 9.28 | 1 | 0.95 | 1.0 | 0.18 | 1 |
| HUGLI | WEST BENGAL | 27.26 | 8 | 2.11 | 9 | 43.81 | 9 | 8.46 | 9 | 3.23 | 9.0 | 1.49 | 9 |
| BANKURA | WEST BENGAL | 27.93 | 8 | 1.90 | 8 | 45.19 | 8 | 8.48 | 8 | 2.61 | 8.0 | 1.02 | 8 |
| PURULIYA | WEST BENGAL | 28.81 | 9 | 2.50 | 10 | 44.46 | 8 | 9.17 | 9 | 2.46 | 8.0 | 1.31 | 9 |
| HAORA | WEST BENGAL | 31.39 | 10 | 2.41 | 10 | 31.57 | 1 | 8.09 | 1 | 0.94 | 1.0 | 0.21 | 1 |
| KOLKATA | WEST BENGAL | 29.74 | 10 | 2.22 | 10 | 25.85 | 3 | 6.93 | 4 | 1.47 | 3.0 | 0.51 | 4 |
| SOUTH TWENTY FOUR PARGANAS | WEST BENGAL | 28.61 | 9 | 1.83 | 7 | 31.60 | 4 | 7.01 | 7 | 1.62 | 4.0 | 0.94 | 7 |
| PASCHIM MEDINIPUR | WEST BENGAL | 29.24 | 9 | 1.97 | 9 | 48.78 | 5 | 8.20 | 4 | 1.81 | 5.0 | 0.56 | 4 |
| PURBA MEDINIPUR | WEST BENGAL | 31.76 | 10 | 2.06 | 9 | 34.85 | 5 | 8.07 | 5 | 1.82 | 5.0 | 0.67 | 5 |
| GARHWA | JHARKHAND | 28.44 | 9 | 1.70 | 6 | 31.95 | 3 | 5.96 | 3 | 1.40 | 3.0 | 0.40 | 3 |
| CHATRA | JHARKHAND | 29.29 | 9 | 1.59 | 4 | 25.75 | 2 | 6.08 | 2 | 1.29 | 2.0 | 0.36 | 2 |
| KODARMA | JHARKHAND | 31.09 | 10 | 1.91 | 8 | 30.94 | 3 | 6.90 | 3 | 1.42 | 3.0 | 0.41 | 3 |
| GIRIDIH | JHARKHAND | 28.76 | 9 | 1.74 | 6 | 32.45 | 4 | 6.40 | 4 | 1.54 | 4.0 | 0.55 | 4 |
| DEOGHAR | JHARKHAND | 29.44 | 10 | 1.71 | 6 | 37.45 | 4 | 6.56 | 3 | 1.52 | 4.0 | 0.44 | 3 |
| GODDA | JHARKHAND | 28.53 | 9 | 1.75 | 6 | 35.69 | 2 | 7.90 | 1 | 1.15 | 2.0 | 0.24 | 1 |
| SAHIBGANJ | JHARKHAND | 29.86 | 10 | 1.82 | 7 | 36.78 | 6 | 7.94 | 7 | 1.86 | 6.0 | 0.87 | 7 |
| PAKUR | JHARKHAND | 26.90 | 8 | 1.37 | 2 | 49.28 | 6 | 7.79 | 8 | 1.96 | 6.0 | 1.05 | 8 |
| DHANBAD | JHARKHAND | 29.05 | 9 | 1.90 | 8 | 31.71 | 5 | 7.17 | 6 | 1.69 | 5.0 | 0.75 | 6 |
| BOKARO | JHARKHAND | 29.85 | 10 | 1.67 | 5 | 35.53 | 4 | 6.32 | 4 | 1.65 | 4.0 | 0.55 | 4 |
| LOHARDAGA | JHARKHAND | 28.59 | 9 | 2.24 | 10 | 30.92 | 5 | 6.10 | 6 | 1.72 | 5.0 | 0.71 | 6 |
| PURBI SINGHBHUM | JHARKHAND | 28.38 | 9 | 1.96 | 8 | 31.48 | 7 | 8.60 | 8 | 2.17 | 7.0 | 1.04 | 8 |
| PALAMU | JHARKHAND | 28.42 | 9 | 2.05 | 9 | 30.38 | 4 | 4.96 | 3 | 1.55 | 4.0 | 0.44 | 3 |
| LATEHAR | JHARKHAND | 29.68 | 10 | 1.96 | 8 | 30.37 | 4 | 7.49 | 4 | 1.48 | 4.0 | 0.54 | 4 |
| HAZARIBAGH | JHARKHAND | 28.68 | 9 | 1.93 | 8 | 26.48 | 1 | 7.65 | 1 | 0.85 | 1.0 | 0.19 | 1 |
| RAMGARH | JHARKHAND | 26.71 | 8 | 1.70 | 6 | 34.44 | 6 | 8.59 | 7 | 1.99 | 6.0 | 0.91 | 7 |
| DUMKA | JHARKHAND | 30.14 | 10 | 2.05 | 9 | 39.26 | 6 | 9.43 | 6 | 1.91 | 6.0 | 0.73 | 6 |
| JAMTARA | JHARKHAND | 29.88 | 10 | 1.98 | 9 | 42.42 | 7 | 8.84 | 7 | 2.24 | 7.0 | 0.88 | 7 |
| RANCHI | JHARKHAND | 28.52 | 9 | 2.23 | 10 | 28.43 | 2 | 6.47 | 2 | 1.17 | 2.0 | 0.38 | 2 |
| KHUNTI | JHARKHAND | 29.66 | 10 | 2.12 | 9 | 37.30 | 2 | 11.19 | 4 | 0.98 | 2.0 | 0.49 | 4 |
| GUMLA | JHARKHAND | 30.22 | 10 | 2.42 | 10 | 27.19 | 1 | 10.46 | 1 | 0.90 | 1.0 | 0.18 | 1 |
| SIMDEGA | JHARKHAND | 31.20 | 10 | 2.06 | 9 | 35.21 | 3 | 8.06 | 3 | 1.39 | 3.0 | 0.48 | 3 |
| PASHCHIMI SINGHBHUM | JHARKHAND | 32.14 | 10 | 2.01 | 9 | 36.00 | 2 | 8.06 | 2 | 1.01 | 2.0 | 0.26 | 2 |
| SARAIKELA-KHARSAWAN | JHARKHAND | 28.87 | 9 | 2.17 | 10 | 39.31 | 2 | 10.98 | 3 | 1.11 | 2.0 | 0.46 | 3 |
| BARGARH | ODISHA | 29.30 | 9 | 1.73 | 6 | 29.79 | 6 | 5.57 | 6 | 1.89 | 6.0 | 0.68 | 6 |
| JHARSUGUDA | ODISHA | 30.76 | 10 | 2.16 | 10 | 33.44 | 3 | 7.50 | 2 | 1.43 | 3.0 | 0.36 | 2 |
| SAMBALPUR | ODISHA | 29.09 | 9 | 1.90 | 8 | 34.59 | 4 | 6.97 | 3 | 1.48 | 4.0 | 0.41 | 3 |
| DEBAGARH | ODISHA | 31.13 | 10 | 1.83 | 7 | 36.51 | 5 | 6.33 | 7 | 1.81 | 5.0 | 0.82 | 7 |
| SUNDARGARH | ODISHA | 29.36 | 9 | 1.95 | 8 | 36.84 | 5 | 6.30 | 4 | 1.77 | 5.0 | 0.52 | 4 |
| KENDUJHAR | ODISHA | 28.16 | 8 | 1.80 | 7 | 37.78 | 5 | 7.97 | 5 | 1.83 | 5.0 | 0.64 | 5 |
| MAYURBHANJ | ODISHA | 28.23 | 9 | 1.83 | 7 | 40.29 | 7 | 9.10 | 8 | 2.23 | 7.0 | 0.97 | 8 |
| BALESHWAR | ODISHA | 27.16 | 8 | 1.91 | 8 | 31.51 | 7 | 6.69 | 5 | 2.05 | 7.0 | 0.65 | 5 |
| BHADRAK | ODISHA | 29.01 | 9 | 1.93 | 8 | 30.78 | 7 | 6.49 | 6 | 2.06 | 7.0 | 0.76 | 6 |
| KENDRAPARA | ODISHA | 28.58 | 9 | 1.68 | 5 | 28.70 | 2 | 6.24 | 2 | 1.30 | 2.0 | 0.35 | 2 |
| JAGATSINGHAPUR | ODISHA | 26.48 | 7 | 1.76 | 6 | 31.07 | 6 | 5.80 | 5 | 1.95 | 6.0 | 0.67 | 5 |
| CUTTACK | ODISHA | 28.96 | 9 | 1.72 | 6 | 31.97 | 7 | 6.22 | 7 | 2.22 | 7.0 | 0.94 | 7 |
| JAJAPUR | ODISHA | 27.44 | 8 | 1.52 | 4 | 31.82 | 9 | 5.95 | 8 | 2.82 | 9.0 | 1.00 | 8 |
| DHENKANAL | ODISHA | 28.54 | 9 | 1.53 | 4 | 33.11 | 7 | 5.87 | 6 | 2.28 | 7.0 | 0.68 | 6 |
| ANUGUL | ODISHA | 28.44 | 9 | 1.73 | 6 | 42.72 | 6 | 8.04 | 7 | 2.00 | 6.0 | 0.82 | 7 |
| NAYAGARH | ODISHA | 31.44 | 10 | 1.91 | 8 | 27.60 | 3 | 5.40 | 2 | 1.47 | 3.0 | 0.36 | 2 |
| KHORDHA | ODISHA | 32.49 | 10 | 2.00 | 9 | 31.46 | 2 | 6.22 | 1 | 0.96 | 2.0 | 0.20 | 1 |
| PURI | ODISHA | 31.54 | 10 | 2.21 | 10 | 28.19 | 3 | 6.79 | 2 | 1.41 | 3.0 | 0.39 | 2 |
| GANJAM | ODISHA | 27.20 | 8 | 1.98 | 9 | 27.54 | 7 | 5.56 | 6 | 2.27 | 7.0 | 0.74 | 6 |
| GAJAPATI | ODISHA | 31.86 | 10 | 1.57 | 4 | 30.17 | 3 | 7.16 | 3 | 1.39 | 3.0 | 0.44 | 3 |
| KANDHAMAL | ODISHA | 26.32 | 7 | 1.63 | 5 | 22.45 | 2 | 6.10 | 2 | 1.24 | 2.0 | 0.30 | 2 |
| BAUDH | ODISHA | 27.14 | 8 | 2.13 | 9 | 25.18 | 5 | 6.45 | 4 | 1.82 | 5.0 | 0.58 | 4 |
| SUBARNAPUR | ODISHA | 28.73 | 9 | 1.60 | 4 | 28.18 | 3 | 7.04 | 2 | 1.45 | 3.0 | 0.38 | 2 |
| BALANGIR | ODISHA | 29.02 | 9 | 2.24 | 10 | 26.36 | 8 | 7.06 | 9 | 2.41 | 8.0 | 1.28 | 9 |
| NUAPADA | ODISHA | 29.43 | 10 | 1.61 | 5 | 31.38 | 4 | 5.39 | 4 | 1.61 | 4.0 | 0.52 | 4 |
| KALAHANDI | ODISHA | 29.06 | 9 | 1.63 | 5 | 31.37 | 7 | 6.97 | 8 | 2.25 | 7.0 | 1.01 | 8 |
| RAYAGADA | ODISHA | 31.01 | 10 | 1.91 | 8 | 32.35 | 8 | 7.19 | 8 | 2.43 | 8.0 | 0.96 | 8 |
| NABARANGAPUR | ODISHA | 29.24 | 9 | 1.90 | 8 | 35.71 | 7 | 6.57 | 6 | 2.11 | 7.0 | 0.76 | 6 |
| KORAPUT | ODISHA | 27.57 | 8 | 2.23 | 10 | 29.13 | 5 | 6.22 | 4 | 1.69 | 5.0 | 0.54 | 4 |
| MALKANGIRI | ODISHA | 28.29 | 9 | 1.93 | 8 | 40.60 | 5 | 9.97 | 3 | 1.69 | 5.0 | 0.46 | 3 |
| KORIYA | CHHATTISGARH | 28.66 | 9 | 1.78 | 7 | 32.20 | 5 | 6.61 | 5 | 1.81 | 5.0 | 0.65 | 5 |
| JASHPUR | CHHATTISGARH | 27.67 | 8 | 2.02 | 9 | 30.28 | 7 | 5.94 | 7 | 2.06 | 7.0 | 0.83 | 7 |
| RAIGARH | CHHATTISGARH | 26.47 | 7 | 1.92 | 8 | 31.74 | 8 | 6.78 | 9 | 2.65 | 8.0 | 1.50 | 9 |
| KORBA | CHHATTISGARH | 27.12 | 8 | 1.67 | 5 | 35.22 | 8 | 5.99 | 9 | 2.61 | 8.0 | 1.34 | 9 |
| JANJGIR - CHAMPA | CHHATTISGARH | 25.78 | 7 | 1.53 | 4 | 36.30 | 9 | 5.92 | 8 | 2.93 | 9.0 | 1.05 | 8 |
| KABEERDHAM | CHHATTISGARH | 24.87 | 6 | 1.41 | 2 | 20.92 | 1 | 4.61 | 1 | 0.93 | 1.0 | 0.17 | 1 |
| RAJNANDGAON | CHHATTISGARH | 27.03 | 8 | 2.23 | 10 | 29.16 | 4 | 5.78 | 2 | 1.49 | 4.0 | 0.39 | 2 |
| MAHASAMUND | CHHATTISGARH | 27.28 | 8 | 2.07 | 9 | 32.21 | 7 | 7.06 | 7 | 2.20 | 7.0 | 0.90 | 7 |
| DHAMTARI | CHHATTISGARH | 27.02 | 8 | 1.83 | 7 | 30.90 | 5 | 6.83 | 5 | 1.74 | 5.0 | 0.65 | 5 |
| UTTAR BASTAR KANKER | CHHATTISGARH | 28.41 | 9 | 1.74 | 6 | 33.00 | 6 | 5.13 | 4 | 1.85 | 6.0 | 0.58 | 4 |
| NARAYANPUR | CHHATTISGARH | 31.19 | 10 | 2.18 | 10 | 37.59 | 2 | 7.38 | 2 | 1.17 | 2.0 | 0.34 | 2 |
| BIJAPUR | CHHATTISGARH | 29.01 | 9 | 1.81 | 7 | 38.86 | 4 | 7.20 | 5 | 1.61 | 4.0 | 0.65 | 5 |
| SHEOPUR | MADHYA PRADESH | 25.76 | 7 | 1.77 | 7 | 34.95 | 6 | 6.21 | 6 | 1.89 | 6.0 | 0.71 | 6 |
| MORENA | MADHYA PRADESH | 26.78 | 8 | 1.37 | 2 | 35.13 | 7 | 7.69 | 8 | 2.30 | 7.0 | 0.97 | 8 |
| BHIND | MADHYA PRADESH | 26.72 | 8 | 1.82 | 7 | 35.23 | 9 | 7.30 | 8 | 3.00 | 9.0 | 1.06 | 8 |
| GWALIOR | MADHYA PRADESH | 25.34 | 6 | 1.50 | 3 | 31.90 | 8 | 6.02 | 8 | 2.33 | 8.0 | 0.97 | 8 |
| DATIA | MADHYA PRADESH | 25.41 | 6 | 1.49 | 3 | 29.31 | 7 | 6.64 | 7 | 2.15 | 7.0 | 0.87 | 7 |
| SHIVPURI | MADHYA PRADESH | 25.82 | 7 | 1.78 | 7 | 22.66 | 7 | 4.74 | 6 | 2.13 | 7.0 | 0.69 | 6 |
| TIKAMGARH | MADHYA PRADESH | 25.85 | 7 | 1.09 | 1 | 21.66 | 3 | 4.93 | 2 | 1.31 | 3.0 | 0.31 | 2 |
| CHHATARPUR | MADHYA PRADESH | 25.14 | 6 | 1.60 | 4 | 32.76 | 9 | 5.73 | 9 | 3.27 | 9.0 | 1.44 | 9 |
| PANNA | MADHYA PRADESH | 27.90 | 8 | 1.79 | 7 | 26.19 | 6 | 6.11 | 6 | 1.92 | 6.0 | 0.75 | 6 |
| SAGAR | MADHYA PRADESH | 24.21 | 5 | 1.76 | 6 | 22.67 | 8 | 5.78 | 6 | 2.45 | 8.0 | 0.70 | 6 |
| DAMOH | MADHYA PRADESH | 22.64 | 3 | 1.39 | 2 | 23.41 | 7 | 5.66 | 5 | 2.07 | 7.0 | 0.63 | 5 |
| SATNA | MADHYA PRADESH | 24.86 | 6 | 1.73 | 6 | 29.50 | 5 | 8.10 | 5 | 1.83 | 5.0 | 0.62 | 5 |
| REWA | MADHYA PRADESH | 27.64 | 8 | 1.93 | 8 | 28.89 | 6 | 7.55 | 5 | 1.97 | 6.0 | 0.60 | 5 |
| UMARIA | MADHYA PRADESH | 25.33 | 6 | 1.72 | 6 | 24.05 | 4 | 6.22 | 3 | 1.53 | 4.0 | 0.46 | 3 |
| NEEMUCH | MADHYA PRADESH | 24.27 | 5 | 1.51 | 3 | 23.61 | 4 | 4.68 | 8 | 1.63 | 4.0 | 0.96 | 8 |
| MANDSAUR | MADHYA PRADESH | 23.31 | 4 | 1.36 | 2 | 30.40 | 6 | 5.78 | 7 | 2.03 | 6.0 | 0.91 | 7 |
| RATLAM | MADHYA PRADESH | 26.86 | 8 | 2.02 | 9 | 28.26 | 6 | 7.53 | 4 | 1.88 | 6.0 | 0.52 | 4 |
| UJJAIN | MADHYA PRADESH | 24.25 | 5 | 1.95 | 8 | 26.85 | 4 | 5.94 | 6 | 1.67 | 4.0 | 0.74 | 6 |
| DEWAS | MADHYA PRADESH | 23.45 | 4 | 1.70 | 6 | 24.75 | 5 | 6.90 | 6 | 1.80 | 5.0 | 0.70 | 6 |
| DHAR | MADHYA PRADESH | 28.62 | 9 | 2.49 | 10 | 20.36 | 5 | 6.81 | 5 | 1.72 | 5.0 | 0.67 | 5 |
| INDORE | MADHYA PRADESH | 26.04 | 7 | 2.11 | 9 | 19.58 | 4 | 5.01 | 4 | 1.58 | 4.0 | 0.55 | 4 |
| KHARGONE (WEST NIMAR) | MADHYA PRADESH | 24.08 | 5 | 1.52 | 4 | 23.92 | 5 | 7.92 | 5 | 1.74 | 5.0 | 0.61 | 5 |
| BARWANI | MADHYA PRADESH | 27.95 | 8 | 2.27 | 10 | 26.64 | 4 | 6.36 | 5 | 1.48 | 4.0 | 0.62 | 5 |
| RAJGARH | MADHYA PRADESH | 25.83 | 7 | 1.56 | 4 | 24.36 | 3 | 5.00 | 2 | 1.47 | 3.0 | 0.29 | 2 |
| VIDISHA | MADHYA PRADESH | 23.36 | 4 | 1.19 | 1 | 16.39 | 1 | 3.34 | 1 | 0.79 | 1.0 | 0.12 | 1 |
| BHOPAL | MADHYA PRADESH | 25.01 | 6 | 1.84 | 7 | 24.18 | 6 | 4.70 | 6 | 1.94 | 6.0 | 0.75 | 6 |
| SEHORE | MADHYA PRADESH | 22.08 | 2 | 1.34 | 2 | 22.26 | 6 | 5.46 | 7 | 1.92 | 6.0 | 0.95 | 7 |
| RAISEN | MADHYA PRADESH | 26.08 | 7 | 1.69 | 5 | 26.58 | 3 | 5.19 | 1 | 1.41 | 3.0 | 0.24 | 1 |
| BETUL | MADHYA PRADESH | 25.71 | 7 | 1.96 | 8 | 26.73 | 5 | 6.40 | 2 | 1.69 | 5.0 | 0.39 | 2 |
| HARDA | MADHYA PRADESH | 26.45 | 7 | 1.96 | 8 | 31.23 | 7 | 6.87 | 8 | 2.20 | 7.0 | 0.97 | 8 |
| HOSHANGABAD | MADHYA PRADESH | 23.76 | 4 | 1.46 | 3 | 25.12 | 8 | 4.93 | 8 | 2.51 | 8.0 | 1.12 | 8 |
| KATNI | MADHYA PRADESH | 26.02 | 7 | 1.68 | 5 | 28.64 | 6 | 5.43 | 5 | 2.03 | 6.0 | 0.59 | 5 |
| JABALPUR | MADHYA PRADESH | 24.80 | 6 | 0.80 | 1 | 22.28 | 5 | 5.31 | 4 | 1.82 | 5.0 | 0.54 | 4 |
| NARSIMHAPUR | MADHYA PRADESH | 21.38 | 2 | 1.44 | 3 | 25.19 | 3 | 5.23 | 3 | 1.44 | 3.0 | 0.45 | 3 |
| DINDORI | MADHYA PRADESH | 26.49 | 7 | 1.75 | 6 | 32.53 | 5 | 8.34 | 5 | 1.71 | 5.0 | 0.61 | 5 |
| MANDLA | MADHYA PRADESH | 26.35 | 7 | 1.62 | 5 | 29.87 | 6 | 7.68 | 5 | 1.94 | 6.0 | 0.66 | 5 |
| CHHINDWARA | MADHYA PRADESH | 21.43 | 2 | 1.48 | 3 | 20.61 | 3 | 5.78 | 2 | 1.43 | 3.0 | 0.39 | 2 |
| SEONI | MADHYA PRADESH | 27.24 | 8 | 2.13 | 9 | 28.01 | 5 | 6.71 | 6 | 1.73 | 5.0 | 0.69 | 6 |
| BALAGHAT | MADHYA PRADESH | 29.68 | 10 | 1.87 | 8 | 27.84 | 1 | 4.82 | 1 | 0.95 | 1.0 | 0.19 | 1 |
| GUNA | MADHYA PRADESH | 25.86 | 7 | 1.88 | 8 | 21.74 | 2 | 5.52 | 1 | 1.03 | 2.0 | 0.20 | 1 |
| ASHOKNAGAR | MADHYA PRADESH | 23.89 | 4 | 1.45 | 3 | 21.12 | 3 | 4.30 | 4 | 1.42 | 3.0 | 0.50 | 4 |
| SHAHDOL | MADHYA PRADESH | 25.38 | 6 | 1.34 | 2 | 27.72 | 4 | 6.73 | 2 | 1.65 | 4.0 | 0.39 | 2 |
| ANUPPUR | MADHYA PRADESH | 25.76 | 7 | 1.56 | 4 | 24.27 | 4 | 4.91 | 3 | 1.54 | 4.0 | 0.43 | 3 |
| SIDHI | MADHYA PRADESH | 25.14 | 6 | 1.86 | 8 | 27.85 | 4 | 6.00 | 5 | 1.56 | 4.0 | 0.59 | 5 |
| SINGRAULI | MADHYA PRADESH | 26.47 | 7 | 1.50 | 3 | 25.06 | 2 | 5.38 | 1 | 1.16 | 2.0 | 0.20 | 1 |
| JHABUA | MADHYA PRADESH | 25.88 | 7 | 1.60 | 4 | 29.57 | 3 | 5.63 | 2 | 1.33 | 3.0 | 0.35 | 2 |
| ALIRAJPUR | MADHYA PRADESH | 25.87 | 7 | 1.74 | 6 | 30.10 | 4 | 6.46 | 4 | 1.67 | 4.0 | 0.53 | 4 |
| KHANDWA (EAST NIMAR) | MADHYA PRADESH | 26.83 | 8 | 2.19 | 10 | 30.08 | 7 | 6.92 | 4 | 2.20 | 7.0 | 0.53 | 4 |
| BURHANPUR | MADHYA PRADESH | 26.36 | 7 | 2.10 | 9 | 27.43 | 6 | 7.66 | 8 | 1.99 | 6.0 | 0.98 | 8 |
| KACHCHH | GUJARAT | 24.68 | 5 | 1.65 | 5 | 28.79 | 8 | 6.46 | 9 | 2.59 | 8.0 | 1.25 | 9 |
| BANAS KANTHA | GUJARAT | 23.58 | 4 | 1.61 | 5 | 31.91 | 9 | 6.43 | 8 | 3.06 | 9.0 | 1.14 | 8 |
| PATAN | GUJARAT | 24.39 | 5 | 1.93 | 8 | 30.79 | 9 | 6.49 | 9 | 3.35 | 9.0 | 1.27 | 9 |
| MAHESANA | GUJARAT | 23.01 | 3 | 1.28 | 1 | 40.44 | 10 | 6.68 | 10 | 4.81 | 10.0 | 2.18 | 10 |
| GANDHINAGAR | GUJARAT | 23.24 | 4 | 1.18 | 1 | 37.41 | 10 | 6.47 | 10 | 4.81 | 10.0 | 2.66 | 10 |
| PORBANDAR | GUJARAT | 24.11 | 5 | 1.85 | 7 | 22.01 | 6 | 6.19 | 6 | 1.91 | 6.0 | 0.79 | 6 |
| AMRELI | GUJARAT | 24.14 | 5 | 1.42 | 2 | 25.37 | 5 | 4.60 | 3 | 1.78 | 5.0 | 0.47 | 3 |
| ANAND | GUJARAT | 24.56 | 5 | 1.55 | 4 | 35.26 | 10 | 5.85 | 10 | 4.45 | 10.0 | 2.04 | 10 |
| DOHAD | GUJARAT | 27.30 | 8 | 1.57 | 4 | 39.86 | 10 | 8.52 | 10 | 4.26 | 10.0 | 2.92 | 10 |
| NARMADA | GUJARAT | 28.85 | 9 | 1.83 | 7 | 39.85 | 9 | 7.06 | 10 | 3.09 | 9.0 | 1.54 | 10 |
| BHARUCH | GUJARAT | 25.92 | 7 | 1.47 | 3 | 39.30 | 10 | 5.71 | 10 | 4.21 | 10.0 | 2.28 | 10 |
| THE DANGS | GUJARAT | 30.29 | 10 | 1.94 | 8 | 40.22 | 7 | 7.30 | 7 | 2.30 | 7.0 | 0.87 | 7 |
| NAVSARI | GUJARAT | 27.32 | 8 | 1.77 | 7 | 35.49 | 8 | 8.76 | 8 | 2.71 | 8.0 | 1.14 | 8 |
| VALSAD | GUJARAT | 27.56 | 8 | 1.73 | 6 | 41.39 | 10 | 9.76 | 9 | 3.51 | 10.0 | 1.48 | 9 |
| SURAT | GUJARAT | 27.03 | 8 | 1.23 | 1 | 36.85 | 9 | 6.59 | 9 | 2.96 | 9.0 | 1.26 | 9 |
| TAPI | GUJARAT | 26.54 | 7 | 1.86 | 8 | 43.79 | 10 | 6.61 | 9 | 3.57 | 10.0 | 1.28 | 9 |
| DIU | DADRA & NAGAR HAVELI AND DAMAN & DIU | 28.16 | 8 | 1.72 | 6 | 20.48 | 2 | 3.76 | 2 | 1.08 | 2.0 | 0.27 | 2 |
| DAMAN | DADRA & NAGAR HAVELI AND DAMAN & DIU | 25.22 | 6 | 1.59 | 4 | 31.42 | 8 | 6.32 | 6 | 2.64 | 8.0 | 0.74 | 6 |
| DADRA & NAGAR HAVELI | DADRA & NAGAR HAVELI AND DAMAN & DIU | 29.55 | 10 | 2.20 | 10 | 30.82 | 3 | 5.94 | 4 | 1.45 | 3.0 | 0.53 | 4 |
| NANDURBAR | MAHARASHTRA | 25.14 | 6 | 2.02 | 9 | 33.52 | 8 | 7.56 | 8 | 2.36 | 8.0 | 1.03 | 8 |
| DHULE | MAHARASHTRA | 23.61 | 4 | 1.40 | 2 | 32.03 | 10 | 6.16 | 10 | 3.57 | 10.0 | 1.78 | 10 |
| JALGAON | MAHARASHTRA | 25.56 | 6 | 1.38 | 2 | 32.76 | 9 | 5.83 | 8 | 3.06 | 9.0 | 1.07 | 8 |
| BULDANA | MAHARASHTRA | 25.38 | 6 | 2.32 | 10 | 27.20 | 7 | 5.43 | 8 | 2.19 | 7.0 | 1.06 | 8 |
| AKOLA | MAHARASHTRA | 24.65 | 5 | 2.13 | 9 | 25.15 | 4 | 5.84 | 4 | 1.63 | 4.0 | 0.51 | 4 |
| WASHIM | MAHARASHTRA | 21.79 | 2 | 1.83 | 7 | 30.21 | 9 | 7.97 | 8 | 2.73 | 9.0 | 1.10 | 8 |
| AMRAVATI | MAHARASHTRA | 23.89 | 4 | 1.33 | 2 | 25.27 | 8 | 5.39 | 9 | 2.61 | 8.0 | 1.32 | 9 |
| WARDHA | MAHARASHTRA | 26.23 | 7 | 1.51 | 3 | 28.63 | 8 | 4.71 | 7 | 2.44 | 8.0 | 0.94 | 7 |
| NAGPUR | MAHARASHTRA | 25.42 | 6 | 1.93 | 8 | 24.67 | 4 | 6.32 | 4 | 1.64 | 4.0 | 0.57 | 4 |
| BHANDARA | MAHARASHTRA | 28.48 | 9 | 1.95 | 8 | 29.91 | 6 | 6.40 | 5 | 1.87 | 6.0 | 0.62 | 5 |
| GONDIYA | MAHARASHTRA | 25.19 | 6 | 1.58 | 4 | 31.76 | 3 | 6.73 | 3 | 1.42 | 3.0 | 0.45 | 3 |
| GADCHIROLI | MAHARASHTRA | 25.39 | 6 | 1.74 | 6 | 35.21 | 6 | 8.54 | 5 | 1.91 | 6.0 | 0.63 | 5 |
| CHANDRAPUR | MAHARASHTRA | 25.30 | 6 | 1.93 | 8 | 27.72 | 3 | 6.55 | 4 | 1.38 | 3.0 | 0.49 | 4 |
| YAVATMAL | MAHARASHTRA | 23.16 | 3 | 1.51 | 3 | 30.10 | 9 | 6.38 | 7 | 2.77 | 9.0 | 0.92 | 7 |
| NANDED | MAHARASHTRA | 21.63 | 2 | 1.56 | 4 | 31.31 | 9 | 7.82 | 8 | 2.77 | 9.0 | 1.08 | 8 |
| HINGOLI | MAHARASHTRA | 25.04 | 6 | 1.87 | 8 | 22.55 | 6 | 5.41 | 5 | 1.91 | 6.0 | 0.64 | 5 |
| PARBHANI | MAHARASHTRA | 22.59 | 3 | 1.46 | 3 | 32.21 | 8 | 5.91 | 8 | 2.63 | 8.0 | 1.01 | 8 |
| JALNA | MAHARASHTRA | 23.98 | 5 | 1.44 | 3 | 28.39 | 9 | 4.91 | 9 | 3.10 | 9.0 | 1.32 | 9 |
| AURANGABAD | MAHARASHTRA | 24.36 | 5 | 1.57 | 4 | 24.36 | 5 | 4.64 | 5 | 1.70 | 5.0 | 0.59 | 5 |
| NASHIK | MAHARASHTRA | 24.28 | 5 | 1.41 | 2 | 27.27 | 9 | 4.82 | 9 | 2.76 | 9.0 | 1.19 | 9 |
| MUMBAI SUBURBAN | MAHARASHTRA | 23.59 | 4 | 0.94 | 1 | 22.81 | 6 | 4.00 | 4 | 1.98 | 6.0 | 0.49 | 4 |
| MUMBAI | MAHARASHTRA | 26.43 | 7 | 1.75 | 6 | 17.29 | 2 | 4.27 | 2 | 1.25 | 2.0 | 0.36 | 2 |
| RAIGARH | MAHARASHTRA | 25.61 | 6 | 1.73 | 6 | 23.76 | 4 | 6.15 | 3 | 1.63 | 4.0 | 0.45 | 3 |
| PUNE | MAHARASHTRA | 21.96 | 2 | 1.26 | 1 | 25.35 | 8 | 6.10 | 9 | 2.71 | 8.0 | 1.19 | 9 |
| AHMADNAGAR | MAHARASHTRA | 22.37 | 3 | 1.60 | 4 | 23.91 | 8 | 5.35 | 8 | 2.70 | 8.0 | 1.14 | 8 |
| BID | MAHARASHTRA | 22.05 | 2 | 1.33 | 2 | 26.73 | 2 | 5.98 | 2 | 1.26 | 2.0 | 0.26 | 2 |
| LATUR | MAHARASHTRA | 23.52 | 4 | 1.91 | 8 | 24.09 | 5 | 4.97 | 5 | 1.79 | 5.0 | 0.59 | 5 |
| OSMANABAD | MAHARASHTRA | 22.32 | 3 | 1.48 | 3 | 24.44 | 7 | 5.96 | 7 | 2.24 | 7.0 | 0.84 | 7 |
| SOLAPUR | MAHARASHTRA | 24.80 | 6 | 1.99 | 9 | 25.80 | 7 | 6.04 | 6 | 2.11 | 7.0 | 0.72 | 6 |
| SATARA | MAHARASHTRA | 23.39 | 4 | 1.61 | 5 | 22.49 | 6 | 5.19 | 6 | 1.95 | 6.0 | 0.69 | 6 |
| RATNAGIRI | MAHARASHTRA | 21.02 | 2 | 1.18 | 1 | 21.82 | 4 | 6.71 | 4 | 1.56 | 4.0 | 0.53 | 4 |
| SINDHUDURG | MAHARASHTRA | 21.65 | 2 | 1.25 | 1 | 19.54 | 4 | 4.79 | 2 | 1.49 | 4.0 | 0.29 | 2 |
| KOLHAPUR | MAHARASHTRA | 23.34 | 4 | 1.59 | 4 | 22.85 | 8 | 5.53 | 7 | 2.59 | 8.0 | 0.94 | 7 |
| SANGLI | MAHARASHTRA | 23.25 | 4 | 1.47 | 3 | 21.85 | 6 | 5.31 | 6 | 1.98 | 6.0 | 0.75 | 6 |
| SRIKAKULAM | ANDHRA PRADESH | 26.82 | 8 | 1.84 | 7 | 30.99 | 8 | 5.30 | 7 | 2.45 | 8.0 | 0.92 | 7 |
| VIZIANAGARAM | ANDHRA PRADESH | 26.47 | 7 | 1.49 | 3 | 31.51 | 9 | 5.04 | 9 | 3.24 | 9.0 | 1.15 | 9 |
| VISAKHAPATNAM | ANDHRA PRADESH | 23.96 | 5 | 1.41 | 2 | 29.67 | 9 | 6.27 | 8 | 3.39 | 9.0 | 1.03 | 8 |
| EAST GODAVARI | ANDHRA PRADESH | 24.29 | 5 | 1.40 | 2 | 30.76 | 10 | 4.79 | 10 | 4.59 | 10.0 | 2.09 | 10 |
| WEST GODAVARI | ANDHRA PRADESH | 26.43 | 7 | 1.64 | 5 | 30.29 | 9 | 5.90 | 8 | 3.22 | 9.0 | 1.13 | 8 |
| KRISHNA | ANDHRA PRADESH | 26.66 | 7 | 1.51 | 3 | 25.57 | 10 | 4.91 | 10 | 4.53 | 10.0 | 2.00 | 10 |
| GUNTUR | ANDHRA PRADESH | 25.78 | 7 | 1.66 | 5 | 28.03 | 9 | 4.88 | 10 | 3.44 | 9.0 | 1.53 | 10 |
| PRAKASAM | ANDHRA PRADESH | 24.08 | 5 | 1.36 | 2 | 30.48 | 10 | 5.08 | 10 | 4.18 | 10.0 | 1.59 | 10 |
| SRI POTTI SRIRAMULU NELLORE | ANDHRA PRADESH | 23.76 | 4 | 1.50 | 3 | 30.93 | 10 | 5.53 | 9 | 3.71 | 10.0 | 1.15 | 9 |
| Y.S.R. | ANDHRA PRADESH | 24.42 | 5 | 1.78 | 7 | 26.36 | 10 | 6.45 | 10 | 3.60 | 10.0 | 1.56 | 10 |
| KURNOOL | ANDHRA PRADESH | 22.83 | 3 | 1.70 | 6 | 30.23 | 10 | 5.93 | 9 | 4.43 | 10.0 | 1.49 | 9 |
| ANANTAPUR | ANDHRA PRADESH | 23.38 | 4 | 1.18 | 1 | 23.64 | 9 | 5.01 | 7 | 3.19 | 9.0 | 0.87 | 7 |
| CHITTOOR | ANDHRA PRADESH | 23.42 | 4 | 1.32 | 2 | 23.90 | 10 | 5.90 | 9 | 3.81 | 10.0 | 1.29 | 9 |
| BELGAUM | KARNATAKA | 22.86 | 3 | 1.55 | 4 | 23.87 | 9 | 5.01 | 10 | 3.12 | 9.0 | 1.76 | 10 |
| BAGALKOT | KARNATAKA | 23.20 | 3 | 1.68 | 5 | 20.59 | 7 | 4.15 | 7 | 2.20 | 7.0 | 0.87 | 7 |
| BIJAPUR | KARNATAKA | 23.33 | 4 | 1.55 | 4 | 23.96 | 8 | 5.65 | 7 | 2.42 | 8.0 | 0.93 | 7 |
| BIDAR | KARNATAKA | 23.61 | 4 | 1.69 | 5 | 27.44 | 5 | 6.80 | 4 | 1.71 | 5.0 | 0.52 | 4 |
| RAICHUR | KARNATAKA | 24.11 | 5 | 1.77 | 7 | 28.51 | 10 | 5.87 | 9 | 3.71 | 10.0 | 1.46 | 9 |
| KOPPAL | KARNATAKA | 22.32 | 3 | 1.46 | 3 | 27.72 | 8 | 6.78 | 7 | 2.45 | 8.0 | 0.92 | 7 |
| GADAG | KARNATAKA | 22.07 | 2 | 1.70 | 6 | 23.20 | 8 | 5.04 | 6 | 2.42 | 8.0 | 0.74 | 6 |
| DHARWAD | KARNATAKA | 23.83 | 4 | 1.37 | 2 | 22.06 | 8 | 5.06 | 8 | 2.59 | 8.0 | 1.13 | 8 |
| UTTARA KANNADA | KARNATAKA | 21.65 | 2 | 1.37 | 2 | 20.91 | 5 | 4.24 | 3 | 1.70 | 5.0 | 0.48 | 3 |
| HAVERI | KARNATAKA | 21.26 | 2 | 1.32 | 2 | 25.82 | 7 | 5.11 | 6 | 2.08 | 7.0 | 0.71 | 6 |
| BELLARY | KARNATAKA | 25.67 | 6 | 2.10 | 9 | 25.06 | 9 | 5.58 | 7 | 2.76 | 9.0 | 0.89 | 7 |
| CHITRADURGA | KARNATAKA | 21.40 | 2 | 1.48 | 3 | 19.09 | 9 | 4.54 | 9 | 2.94 | 9.0 | 1.39 | 9 |
| DAVANAGERE | KARNATAKA | 22.55 | 3 | 1.25 | 1 | 24.04 | 8 | 4.10 | 7 | 2.60 | 8.0 | 0.89 | 7 |
| SHIMOGA | KARNATAKA | 21.81 | 2 | 1.29 | 1 | 17.01 | 7 | 4.14 | 7 | 2.12 | 7.0 | 0.88 | 7 |
| UDUPI | KARNATAKA | 23.97 | 5 | 1.82 | 7 | 19.56 | 5 | 4.65 | 5 | 1.72 | 5.0 | 0.63 | 5 |
| CHIKMAGALUR | KARNATAKA | 19.69 | 1 | 1.42 | 2 | 19.56 | 5 | 4.35 | 6 | 1.71 | 5.0 | 0.73 | 6 |
| TUMKUR | KARNATAKA | 21.78 | 2 | 1.07 | 1 | 23.50 | 8 | 4.18 | 8 | 2.61 | 8.0 | 0.99 | 8 |
| BANGALORE | KARNATAKA | 18.60 | 1 | 0.99 | 1 | 17.11 | 5 | 3.64 | 4 | 1.83 | 5.0 | 0.53 | 4 |
| MANDYA | KARNATAKA | 22.27 | 3 | 1.22 | 1 | 21.17 | 5 | 4.46 | 3 | 1.73 | 5.0 | 0.42 | 3 |
| HASSAN | KARNATAKA | 20.71 | 2 | 1.29 | 1 | 19.57 | 5 | 4.47 | 4 | 1.81 | 5.0 | 0.55 | 4 |
| DAKSHINA KANNADA | KARNATAKA | 22.94 | 3 | 1.73 | 6 | 13.45 | 3 | 3.66 | 3 | 1.44 | 3.0 | 0.43 | 3 |
| KODAGU | KARNATAKA | 21.22 | 2 | 1.49 | 3 | 17.79 | 5 | 4.01 | 3 | 1.68 | 5.0 | 0.47 | 3 |
| MYSORE | KARNATAKA | 22.65 | 3 | 1.47 | 3 | 19.43 | 7 | 3.71 | 6 | 2.20 | 7.0 | 0.78 | 6 |
| CHAMARAJANAGAR | KARNATAKA | 20.21 | 1 | 1.35 | 2 | 22.00 | 9 | 5.04 | 9 | 3.33 | 9.0 | 1.47 | 9 |
| GULBARGA | KARNATAKA | 22.80 | 3 | 1.62 | 5 | 27.67 | 9 | 6.76 | 9 | 2.79 | 9.0 | 1.41 | 9 |
| YADGIR | KARNATAKA | 23.21 | 3 | 1.65 | 5 | 28.55 | 9 | 6.14 | 9 | 2.94 | 9.0 | 1.30 | 9 |
| KOLAR | KARNATAKA | 20.43 | 2 | 1.31 | 2 | 20.19 | 8 | 4.41 | 8 | 2.31 | 8.0 | 1.03 | 8 |
| CHIKKABALLAPURA | KARNATAKA | 19.04 | 1 | 1.40 | 2 | 23.72 | 8 | 5.20 | 6 | 2.67 | 8.0 | 0.72 | 6 |
| BANGALORE RURAL | KARNATAKA | 20.56 | 2 | 1.33 | 2 | 20.95 | 9 | 5.19 | 8 | 2.72 | 9.0 | 1.11 | 8 |
| RAMANAGARA | KARNATAKA | 22.55 | 3 | 1.40 | 2 | 20.44 | 6 | 4.20 | 4 | 1.85 | 6.0 | 0.50 | 4 |
| NORTH GOA | GOA | 23.36 | 4 | 1.67 | 5 | 14.00 | 1 | 4.15 | 1 | 0.74 | 1.0 | 0.14 | 1 |
| SOUTH GOA | GOA | 21.55 | 2 | 1.12 | 1 | 13.69 | 2 | 4.18 | 2 | 1.02 | 2.0 | 0.32 | 2 |
| LAKSHADWEEP | LAKSHADWEEP | 15.01 | 1 | 1.49 | 3 | 9.92 | 1 | 4.13 | 1 | 0.69 | 1.0 | 0.18 | 1 |
| KASARAGOD | KERALA | 19.94 | 1 | 1.22 | 1 | 14.15 | 1 | 3.58 | 1 | 0.69 | 1.0 | 0.16 | 1 |
| KANNUR | KERALA | 21.30 | 2 | 1.48 | 3 | 14.66 | 2 | 4.37 | 3 | 1.16 | 2.0 | 0.47 | 3 |
| WAYANAD | KERALA | 16.62 | 1 | 1.33 | 2 | 10.70 | 2 | 3.25 | 3 | 1.01 | 2.0 | 0.42 | 3 |
| KOZHIKODE | KERALA | 19.49 | 1 | 1.19 | 1 | 9.74 | 1 | 2.33 | 1 | 0.80 | 1.0 | 0.19 | 1 |
| MALAPPURAM | KERALA | 21.78 | 2 | 1.74 | 6 | 10.64 | 1 | 2.94 | 1 | 0.86 | 1.0 | 0.17 | 1 |
| PALAKKAD | KERALA | 23.54 | 4 | 1.62 | 5 | 18.08 | 4 | 4.59 | 5 | 1.66 | 4.0 | 0.67 | 5 |
| THRISSUR | KERALA | 21.16 | 2 | 1.21 | 1 | 18.18 | 2 | 3.91 | 3 | 1.06 | 2.0 | 0.42 | 3 |
| ERNAKULAM | KERALA | 19.67 | 1 | 1.29 | 1 | 11.74 | 1 | 2.87 | 1 | 0.73 | 1.0 | 0.17 | 1 |
| IDUKKI | KERALA | 20.05 | 1 | 1.14 | 1 | 10.87 | 1 | 2.34 | 1 | 0.76 | 1.0 | 0.13 | 1 |
| KOTTAYAM | KERALA | 21.94 | 2 | 0.96 | 1 | 12.43 | 2 | 2.03 | 3 | 1.12 | 2.0 | 0.40 | 3 |
| ALAPPUZHA | KERALA | 20.19 | 1 | 1.19 | 1 | 10.93 | 1 | 2.28 | 1 | 0.76 | 1.0 | 0.12 | 1 |
| PATHANAMTHITTA | KERALA | 21.13 | 2 | 1.24 | 1 | 12.43 | 2 | 2.41 | 2 | 1.01 | 2.0 | 0.30 | 2 |
| KOLLAM | KERALA | 19.66 | 1 | 1.35 | 2 | 14.37 | 2 | 3.14 | 3 | 1.03 | 2.0 | 0.40 | 3 |
| THIRUVANANTHAPURAM | KERALA | 18.60 | 1 | 1.00 | 1 | 13.96 | 1 | 2.80 | 1 | 0.82 | 1.0 | 0.14 | 1 |
| THIRUVALLUR | TAMIL NADU | 23.31 | 4 | 1.39 | 2 | 29.00 | 9 | 5.18 | 7 | 2.76 | 9.0 | 0.89 | 7 |
| CHENNAI | TAMIL NADU | 23.92 | 5 | 1.27 | 1 | 22.06 | 6 | 3.36 | 3 | 1.91 | 6.0 | 0.42 | 3 |
| KANCHEEPURAM | TAMIL NADU | 24.91 | 6 | 1.32 | 2 | 26.40 | 9 | 5.10 | 8 | 2.88 | 9.0 | 1.06 | 8 |
| VELLORE | TAMIL NADU | 21.98 | 2 | 1.42 | 2 | 24.81 | 8 | 4.90 | 7 | 2.70 | 8.0 | 0.89 | 7 |
| TIRUVANNAMALAI | TAMIL NADU | 24.23 | 5 | 1.31 | 2 | 27.04 | 9 | 5.46 | 8 | 3.16 | 9.0 | 1.11 | 8 |
| VILUPPURAM | TAMIL NADU | 23.79 | 4 | 1.53 | 4 | 31.81 | 10 | 6.62 | 10 | 3.73 | 10.0 | 1.62 | 10 |
| SALEM | TAMIL NADU | 20.91 | 2 | 1.12 | 1 | 22.86 | 8 | 5.41 | 7 | 2.62 | 8.0 | 0.94 | 7 |
| NAMAKKAL | TAMIL NADU | 24.05 | 5 | 1.53 | 4 | 22.64 | 9 | 3.87 | 8 | 3.44 | 9.0 | 1.04 | 8 |
| ERODE | TAMIL NADU | 23.54 | 4 | 1.29 | 1 | 24.40 | 9 | 4.42 | 9 | 2.94 | 9.0 | 1.22 | 9 |
| THE NILGIRIS | TAMIL NADU | 23.18 | 3 | 1.74 | 6 | 18.46 | 4 | 5.38 | 5 | 1.59 | 4.0 | 0.60 | 5 |
| DINDIGUL | TAMIL NADU | 21.33 | 2 | 1.60 | 4 | 19.84 | 8 | 4.58 | 7 | 2.71 | 8.0 | 0.92 | 7 |
| KARUR | TAMIL NADU | 23.65 | 4 | 1.38 | 2 | 32.34 | 10 | 5.18 | 10 | 4.96 | 10.0 | 2.16 | 10 |
| TIRUCHIRAPPALLI | TAMIL NADU | 25.05 | 6 | 1.65 | 5 | 29.81 | 10 | 6.00 | 9 | 3.48 | 10.0 | 1.46 | 9 |
| PERAMBALUR | TAMIL NADU | 23.89 | 4 | 1.44 | 3 | 33.88 | 10 | 6.46 | 9 | 3.82 | 10.0 | 1.45 | 9 |
| ARIYALUR | TAMIL NADU | 25.96 | 7 | 1.72 | 6 | 28.61 | 10 | 5.26 | 10 | 4.02 | 10.0 | 1.70 | 10 |
| CUDDALORE | TAMIL NADU | 24.13 | 5 | 1.39 | 2 | 26.57 | 10 | 5.03 | 10 | 4.45 | 10.0 | 2.07 | 10 |
| NAGAPATTINAM | TAMIL NADU | 24.99 | 6 | 1.51 | 3 | 30.82 | 9 | 5.66 | 9 | 3.44 | 9.0 | 1.45 | 9 |
| THIRUVARUR | TAMIL NADU | 24.21 | 5 | 1.52 | 4 | 27.34 | 10 | 4.73 | 9 | 3.84 | 10.0 | 1.26 | 9 |
| THANJAVUR | TAMIL NADU | 25.87 | 7 | 1.54 | 4 | 25.96 | 9 | 4.53 | 7 | 3.02 | 9.0 | 0.91 | 7 |
| PUDUKKOTTAI | TAMIL NADU | 26.21 | 7 | 1.61 | 5 | 28.18 | 9 | 5.36 | 9 | 3.24 | 9.0 | 1.24 | 9 |
| SIVAGANGA | TAMIL NADU | 22.82 | 3 | 1.43 | 3 | 25.96 | 8 | 6.42 | 8 | 2.64 | 8.0 | 0.97 | 8 |
| MADURAI | TAMIL NADU | 23.81 | 4 | 1.43 | 3 | 20.81 | 7 | 5.12 | 5 | 2.13 | 7.0 | 0.66 | 5 |
| THENI | TAMIL NADU | 21.02 | 2 | 1.62 | 5 | 20.63 | 4 | 4.57 | 3 | 1.67 | 4.0 | 0.40 | 3 |
| VIRUDHUNAGAR | TAMIL NADU | 24.48 | 5 | 1.72 | 6 | 27.72 | 8 | 6.83 | 6 | 2.41 | 8.0 | 0.80 | 6 |
| RAMANATHAPURAM | TAMIL NADU | 27.41 | 8 | 1.63 | 5 | 21.70 | 4 | 4.13 | 4 | 1.65 | 4.0 | 0.49 | 4 |
| THOOTHUKKUDI | TAMIL NADU | 27.05 | 8 | 1.77 | 7 | 21.32 | 8 | 5.10 | 6 | 2.61 | 8.0 | 0.79 | 6 |
| TIRUNELVELI | TAMIL NADU | 25.24 | 6 | 1.43 | 3 | 21.96 | 7 | 4.57 | 5 | 2.17 | 7.0 | 0.62 | 5 |
| KANNIYAKUMARI | TAMIL NADU | 23.74 | 4 | 1.42 | 2 | 19.91 | 4 | 4.56 | 2 | 1.54 | 4.0 | 0.30 | 2 |
| DHARMAPURI | TAMIL NADU | 20.95 | 2 | 1.12 | 1 | 21.19 | 4 | 3.85 | 3 | 1.64 | 4.0 | 0.42 | 3 |
| KRISHNAGIRI | TAMIL NADU | 23.59 | 4 | 1.47 | 3 | 18.81 | 5 | 4.44 | 2 | 1.82 | 5.0 | 0.36 | 2 |
| COIMBATORE | TAMIL NADU | 21.34 | 2 | 1.05 | 1 | 26.10 | 8 | 5.18 | 7 | 2.44 | 8.0 | 0.83 | 7 |
| TIRUPPUR | TAMIL NADU | 23.63 | 4 | 1.32 | 2 | 27.41 | 9 | 4.68 | 9 | 2.85 | 9.0 | 1.29 | 9 |
| YANAM | PUDUCHERRY | 21.23 | 2 | 1.14 | 1 | 19.39 | 5 | 4.58 | 5 | 1.73 | 5.0 | 0.62 | 5 |
| PUDUCHERRY | PUDUCHERRY | 22.79 | 3 | 1.31 | 2 | 28.32 | 9 | 6.27 | 8 | 2.74 | 9.0 | 1.14 | 8 |
| MAHE | PUDUCHERRY | 18.46 | 1 | 1.37 | 2 | 20.52 | 1 | 12.26 | 1 | 0.64 | 1.0 | 0.05 | 1 |
| KARAIKAL | PUDUCHERRY | 23.45 | 4 | 2.13 | 9 | 26.48 | 9 | 5.68 | 9 | 2.88 | 9.0 | 1.51 | 9 |
| NICOBARS | ANDAMAN & NICOBAR ISLANDS | 22.75 | 3 | 1.35 | 2 | 16.44 | 2 | 5.74 | 2 | 1.30 | 2.0 | 0.34 | 2 |
| NORTH & MIDDLE ANDAMAN | ANDAMAN & NICOBAR ISLANDS | 27.34 | 8 | 1.70 | 6 | 29.83 | 8 | 6.86 | 8 | 2.40 | 8.0 | 1.00 | 8 |
| SOUTH ANDAMAN | ANDAMAN & NICOBAR ISLANDS | 26.73 | 8 | 2.24 | 10 | 26.08 | 7 | 5.86 | 9 | 2.29 | 7.0 | 1.15 | 9 |
| EAST SIANG | ARUNACHAL PRADESH | 20.03 | 1 | 1.43 | 3 | 13.41 | 1 | 3.31 | 1 | 0.87 | 1.0 | 0.23 | 1 |
| KRA DAADI | ARUNACHAL PRADESH | 17.44 | 1 | 1.48 | 3 | 8.81 | 1 | 1.82 | 1 | 0.69 | 1.0 | 0.13 | 1 |
| KURUNG KUMEY | ARUNACHAL PRADESH | 19.13 | 1 | 1.66 | 5 | 14.96 | 1 | 4.24 | 1 | 0.64 | 1.0 | 0.10 | 1 |
| LOHIT | ARUNACHAL PRADESH | 24.43 | 5 | 1.78 | 7 | 17.86 | 2 | 5.16 | 2 | 0.96 | 2.0 | 0.32 | 2 |
| LONGDING | ARUNACHAL PRADESH | 21.18 | 2 | 1.76 | 6 | 12.28 | 1 | 3.60 | 1 | 0.59 | 1.0 | 0.09 | 1 |
| NAMSAI | ARUNACHAL PRADESH | 24.92 | 6 | 1.38 | 2 | 26.96 | 3 | 6.18 | 3 | 1.39 | 3.0 | 0.43 | 3 |
| SIANG | ARUNACHAL PRADESH | 18.16 | 1 | 1.70 | 6 | 6.89 | 1 | 1.87 | 1 | 0.66 | 1.0 | 0.12 | 1 |
| TIRAP | ARUNACHAL PRADESH | 19.91 | 1 | 1.28 | 1 | 10.96 | 1 | 2.83 | 1 | 0.64 | 1.0 | 0.13 | 1 |
| WEST SIANG | ARUNACHAL PRADESH | 17.82 | 1 | 1.14 | 1 | 11.80 | 2 | 3.70 | 2 | 1.14 | 2.0 | 0.35 | 2 |
| BISWANATH | ASSAM | 27.13 | 8 | 1.61 | 5 | 34.71 | 6 | 7.71 | 6 | 1.94 | 6.0 | 0.71 | 6 |
| CHARAIDEO | ASSAM | 30.69 | 10 | 1.84 | 7 | 37.26 | 4 | 7.28 | 5 | 1.60 | 4.0 | 0.64 | 5 |
| DHUBRI | ASSAM | 27.88 | 8 | 2.17 | 10 | 32.85 | 4 | 7.53 | 5 | 1.58 | 4.0 | 0.59 | 5 |
| HOJAI | ASSAM | 27.29 | 8 | 1.74 | 6 | 27.64 | 4 | 6.72 | 3 | 1.55 | 4.0 | 0.42 | 3 |
| JORHAT | ASSAM | 29.00 | 9 | 1.72 | 6 | 38.57 | 8 | 7.82 | 8 | 2.42 | 8.0 | 1.12 | 8 |
| KARBI ANGLONG | ASSAM | 28.55 | 9 | 1.92 | 8 | 27.86 | 4 | 7.54 | 4 | 1.53 | 4.0 | 0.57 | 4 |
| MAJULI | ASSAM | 30.22 | 10 | 1.76 | 6 | 34.23 | 3 | 7.90 | 2 | 1.37 | 3.0 | 0.38 | 2 |
| NAGAON | ASSAM | 27.74 | 8 | 2.38 | 10 | 30.46 | 5 | 6.80 | 6 | 1.74 | 5.0 | 0.78 | 6 |
| SIVASAGAR | ASSAM | 27.49 | 8 | 1.84 | 7 | 36.04 | 8 | 6.24 | 8 | 2.45 | 8.0 | 1.10 | 8 |
| SONITPUR | ASSAM | 28.25 | 9 | 1.46 | 3 | 32.03 | 5 | 9.99 | 6 | 1.68 | 5.0 | 0.75 | 6 |
| SOUTH SALMARA MANCACHAR | ASSAM | 27.92 | 8 | 1.93 | 8 | 27.31 | 3 | 6.22 | 2 | 1.36 | 3.0 | 0.39 | 2 |
| WEST KARBI ANGLONG | ASSAM | 29.04 | 9 | 1.98 | 9 | 27.58 | 1 | 6.66 | 1 | 0.79 | 1.0 | 0.16 | 1 |
| BALOD | CHHATTISGARH | 25.14 | 6 | 1.59 | 4 | 29.80 | 6 | 5.44 | 5 | 1.91 | 6.0 | 0.64 | 5 |
| BALODA BAZAR | CHHATTISGARH | 28.51 | 9 | 1.87 | 8 | 28.81 | 7 | 5.94 | 8 | 2.17 | 7.0 | 1.00 | 8 |
| BALRAMPUR | CHHATTISGARH | 29.17 | 9 | 1.60 | 4 | 30.36 | 6 | 7.08 | 4 | 1.96 | 6.0 | 0.53 | 4 |
| BASTAR | CHHATTISGARH | 26.68 | 7 | 1.99 | 9 | 44.16 | 9 | 7.89 | 10 | 3.35 | 9.0 | 1.71 | 10 |
| BEMETARA | CHHATTISGARH | 26.20 | 7 | 1.83 | 7 | 21.00 | 2 | 4.93 | 2 | 1.18 | 2.0 | 0.29 | 2 |
| BILASPUR | CHHATTISGARH | 26.61 | 7 | 1.82 | 7 | 28.23 | 8 | 5.91 | 9 | 2.45 | 8.0 | 1.18 | 9 |
| DANTEWADA | CHHATTISGARH | 29.37 | 9 | 1.98 | 9 | 41.66 | 6 | 8.86 | 7 | 1.94 | 6.0 | 0.88 | 7 |
| DURG | CHHATTISGARH | 25.85 | 7 | 1.74 | 6 | 24.57 | 2 | 7.01 | 2 | 1.24 | 2.0 | 0.36 | 2 |
| GARIYABAND | CHHATTISGARH | 27.56 | 8 | 1.51 | 3 | 34.15 | 5 | 7.56 | 5 | 1.75 | 5.0 | 0.67 | 5 |
| KODAGAON | CHHATTISGARH | 27.61 | 8 | 1.99 | 9 | 46.12 | 9 | 5.98 | 10 | 2.87 | 9.0 | 1.65 | 10 |
| MUNGELI | CHHATTISGARH | 24.91 | 6 | 1.62 | 5 | 26.30 | 8 | 5.46 | 8 | 2.35 | 8.0 | 1.13 | 8 |
| RAIPUR | CHHATTISGARH | 28.24 | 9 | 1.95 | 8 | 28.18 | 4 | 6.40 | 7 | 1.67 | 4.0 | 0.82 | 7 |
| SUKMA | CHHATTISGARH | 29.37 | 9 | 2.22 | 10 | 43.71 | 4 | 11.08 | 5 | 1.53 | 4.0 | 0.64 | 5 |
| SURAJPUR | CHHATTISGARH | 28.91 | 9 | 1.96 | 8 | 28.98 | 6 | 4.81 | 5 | 1.98 | 6.0 | 0.66 | 5 |
| SURGUJA | CHHATTISGARH | 28.22 | 9 | 1.98 | 9 | 27.74 | 3 | 5.33 | 3 | 1.37 | 3.0 | 0.41 | 3 |
| CENTRAL | NCT OF DELHI | 20.55 | 2 | 1.21 | 1 | 21.15 | 8 | 4.36 | 9 | 2.62 | 8.0 | 1.21 | 9 |
| EAST | NCT OF DELHI | 22.34 | 3 | 1.73 | 6 | 17.29 | 5 | 4.78 | 5 | 1.81 | 5.0 | 0.67 | 5 |
| NEW DELHI | NCT OF DELHI | 25.22 | 6 | 1.37 | 2 | 23.48 | 9 | 5.50 | 9 | 3.07 | 9.0 | 1.20 | 9 |
| NORTH | NCT OF DELHI | 22.18 | 3 | 1.41 | 2 | 21.79 | 7 | 3.93 | 7 | 2.17 | 7.0 | 0.84 | 7 |
| NORTH EAST | NCT OF DELHI | 22.31 | 3 | 1.67 | 5 | 19.93 | 8 | 4.56 | 7 | 2.37 | 8.0 | 0.83 | 7 |
| NORTH WEST | NCT OF DELHI | 23.92 | 5 | 1.31 | 2 | 23.04 | 6 | 5.62 | 4 | 1.93 | 6.0 | 0.55 | 4 |
| SHAHDARA | NCT OF DELHI | 23.08 | 3 | 1.77 | 7 | 21.27 | 7 | 6.14 | 8 | 2.22 | 7.0 | 1.13 | 8 |
| SOUTH | NCT OF DELHI | 23.19 | 3 | 1.64 | 5 | 20.01 | 7 | 5.08 | 6 | 2.26 | 7.0 | 0.77 | 6 |
| SOUTH EAST | NCT OF DELHI | 24.28 | 5 | 1.67 | 5 | 18.98 | 6 | 5.88 | 5 | 1.88 | 6.0 | 0.65 | 5 |
| SOUTH WEST | NCT OF DELHI | 24.23 | 5 | 1.53 | 4 | 26.27 | 7 | 6.65 | 5 | 2.19 | 7.0 | 0.67 | 5 |
| WEST | NCT OF DELHI | 22.64 | 3 | 1.49 | 3 | 27.77 | 8 | 7.01 | 6 | 2.57 | 8.0 | 0.80 | 6 |
| AHMADABAD | GUJARAT | 27.22 | 8 | 1.70 | 6 | 30.93 | 7 | 6.69 | 6 | 2.24 | 7.0 | 0.75 | 6 |
| ARAVALI | GUJARAT | 24.92 | 6 | 1.71 | 6 | 44.36 | 10 | 8.75 | 10 | 5.52 | 10.0 | 2.85 | 10 |
| BHAVNAGAR | GUJARAT | 23.35 | 4 | 1.76 | 6 | 24.70 | 8 | 5.32 | 8 | 2.46 | 8.0 | 1.08 | 8 |
| BOTAD | GUJARAT | 25.80 | 7 | 1.42 | 2 | 29.84 | 7 | 5.74 | 6 | 2.09 | 7.0 | 0.75 | 6 |
| CHHOTA UDAIPUR | GUJARAT | 27.17 | 8 | 2.05 | 9 | 44.42 | 10 | 8.38 | 10 | 4.06 | 10.0 | 1.88 | 10 |
| DEVBHUMI DWARKA | GUJARAT | 23.95 | 5 | 1.96 | 8 | 23.25 | 5 | 5.68 | 4 | 1.72 | 5.0 | 0.50 | 4 |
| GIR SOMNATH | GUJARAT | 23.56 | 4 | 1.50 | 3 | 25.20 | 5 | 4.53 | 6 | 1.83 | 5.0 | 0.68 | 6 |
| JAMNAGAR | GUJARAT | 26.27 | 7 | 2.28 | 10 | 21.79 | 5 | 5.41 | 6 | 1.71 | 5.0 | 0.79 | 6 |
| JUNAGADH | GUJARAT | 25.29 | 6 | 1.87 | 8 | 30.54 | 7 | 6.03 | 7 | 2.24 | 7.0 | 0.82 | 7 |
| KHEDA | GUJARAT | 23.33 | 4 | 2.03 | 9 | 44.20 | 10 | 7.31 | 10 | 5.87 | 10.0 | 1.78 | 10 |
| MAHISAGAR | GUJARAT | 26.92 | 8 | 1.83 | 7 | 39.32 | 9 | 7.86 | 10 | 3.32 | 9.0 | 1.61 | 10 |
| MORBI | GUJARAT | 24.14 | 5 | 1.69 | 5 | 25.91 | 2 | 5.47 | 2 | 1.30 | 2.0 | 0.31 | 2 |
| PANCH MAHALS | GUJARAT | 24.71 | 5 | 1.59 | 4 | 40.14 | 10 | 7.03 | 10 | 3.80 | 10.0 | 1.75 | 10 |
| RAJKOT | GUJARAT | 25.53 | 6 | 1.61 | 5 | 32.76 | 5 | 8.30 | 3 | 1.73 | 5.0 | 0.43 | 3 |
| SABAR KANTHA | GUJARAT | 25.24 | 6 | 1.65 | 5 | 37.74 | 9 | 6.86 | 9 | 2.79 | 9.0 | 1.29 | 9 |
| SURENDRANAGAR | GUJARAT | 25.92 | 7 | 1.70 | 6 | 27.53 | 7 | 5.56 | 6 | 2.18 | 7.0 | 0.69 | 6 |
| VADODARA | GUJARAT | 26.93 | 8 | 1.60 | 4 | 38.27 | 9 | 8.09 | 9 | 3.19 | 9.0 | 1.16 | 9 |
| BHIWANI | HARYANA | 24.02 | 5 | 1.27 | 1 | 37.15 | 9 | 5.71 | 9 | 3.41 | 9.0 | 1.26 | 9 |
| CHARKHI DADRI | HARYANA | 24.59 | 5 | 1.65 | 5 | 41.33 | 10 | 8.20 | 10 | 3.82 | 10.0 | 1.96 | 10 |
| AGAR MALWA | MADHYA PRADESH | 28.05 | 8 | 1.31 | 2 | 24.65 | 3 | 3.68 | 1 | 1.47 | 3.0 | 0.22 | 1 |
| SHAJAPUR | MADHYA PRADESH | 23.30 | 4 | 1.37 | 2 | 20.91 | 5 | 4.56 | 4 | 1.73 | 5.0 | 0.53 | 4 |
| PALGHAR | MAHARASHTRA | 24.58 | 5 | 1.76 | 6 | 29.06 | 2 | 9.37 | 2 | 1.14 | 2.0 | 0.27 | 2 |
| THANE | MAHARASHTRA | 24.53 | 5 | 1.88 | 8 | 28.82 | 6 | 7.83 | 3 | 1.93 | 6.0 | 0.40 | 3 |
| EAST GARO HILLS | MEGHALAYA | 26.37 | 7 | 2.16 | 10 | 21.62 | 1 | 7.13 | 1 | 0.51 | 1.0 | 0.05 | 1 |
| EAST JANTIA HILLS | MEGHALAYA | 23.19 | 3 | 1.50 | 3 | 19.25 | 3 | 4.90 | 5 | 1.47 | 3.0 | 0.60 | 5 |
| NORTH GARO HILLS | MEGHALAYA | 24.78 | 6 | 2.47 | 10 | 29.81 | 1 | 9.64 | 1 | 0.83 | 1.0 | 0.20 | 1 |
| SOUTH WEST GARO HILLS | MEGHALAYA | 26.62 | 7 | 1.60 | 4 | 32.06 | 2 | 6.60 | 3 | 1.09 | 2.0 | 0.41 | 3 |
| SOUTH WEST KHASI HILLS | MEGHALAYA | 23.87 | 4 | 1.65 | 5 | 30.27 | 6 | 9.08 | 7 | 2.03 | 6.0 | 0.95 | 7 |
| WEST GARO HILLS | MEGHALAYA | 24.15 | 5 | 1.82 | 7 | 28.76 | 2 | 7.89 | 6 | 1.27 | 2.0 | 0.69 | 6 |
| WEST JAINTIA HILLS | MEGHALAYA | 22.84 | 3 | 1.58 | 4 | 29.54 | 8 | 7.98 | 9 | 2.50 | 8.0 | 1.42 | 9 |
| WEST KHASI HILLS | MEGHALAYA | 23.52 | 4 | 1.52 | 4 | 24.27 | 7 | 7.83 | 6 | 2.17 | 7.0 | 0.72 | 6 |
| FAZILKA | PUNJAB | 24.10 | 5 | 1.23 | 1 | 36.28 | 9 | 7.94 | 9 | 3.34 | 9.0 | 1.32 | 9 |
| FIROZPUR | PUNJAB | 25.30 | 6 | 1.78 | 7 | 30.89 | 9 | 6.65 | 10 | 3.15 | 9.0 | 1.56 | 10 |
| GURDASPUR | PUNJAB | 24.59 | 5 | 1.68 | 5 | 27.42 | 5 | 4.64 | 3 | 1.73 | 5.0 | 0.41 | 3 |
| PATHANKOT | PUNJAB | 24.75 | 6 | 1.74 | 6 | 28.48 | 5 | 6.99 | 7 | 1.72 | 5.0 | 0.81 | 7 |
| ADILABAD | TELANGANA | 22.57 | 3 | 1.37 | 2 | 30.26 | 10 | 6.76 | 10 | 4.46 | 10.0 | 1.84 | 10 |
| BHADRADRI KOTHAGUDEM | TELANGANA | 21.52 | 2 | 1.23 | 1 | 38.49 | 10 | 9.13 | 10 | 5.06 | 10.0 | 2.28 | 10 |
| HYDERABAD | TELANGANA | 20.18 | 1 | 0.74 | 1 | 30.11 | 9 | 4.74 | 8 | 2.95 | 9.0 | 1.01 | 8 |
| JAGITIAL | TELANGANA | 22.46 | 3 | 1.83 | 7 | 27.20 | 10 | 5.13 | 10 | 4.54 | 10.0 | 1.75 | 10 |
| JANGOAN | TELANGANA | 21.16 | 2 | 1.23 | 1 | 30.95 | 10 | 5.82 | 10 | 4.13 | 10.0 | 1.55 | 10 |
| JAYASHANKAR BHUPALAPALLY | TELANGANA | 22.66 | 3 | 1.69 | 6 | 36.14 | 10 | 7.32 | 10 | 4.26 | 10.0 | 1.57 | 10 |
| JOGULAMBA GADWAL | TELANGANA | 22.70 | 3 | 1.40 | 2 | 34.88 | 10 | 6.35 | 10 | 4.47 | 10.0 | 1.89 | 10 |
| KAMAREDDY | TELANGANA | 21.85 | 2 | 1.51 | 3 | 31.74 | 10 | 6.51 | 9 | 3.49 | 10.0 | 1.38 | 9 |
| KARIMNAGAR | TELANGANA | 20.92 | 2 | 1.28 | 1 | 34.57 | 10 | 6.57 | 10 | 4.14 | 10.0 | 1.81 | 10 |
| KHAMMAM | TELANGANA | 21.94 | 2 | 1.21 | 1 | 33.21 | 10 | 6.44 | 10 | 4.55 | 10.0 | 2.08 | 10 |
| KOMARAM BHEEM ASIFABAD | TELANGANA | 22.47 | 3 | 1.48 | 3 | 37.55 | 10 | 6.21 | 9 | 4.06 | 10.0 | 1.42 | 9 |
| MAHABUBABAD | TELANGANA | 26.12 | 7 | 1.60 | 4 | 30.04 | 9 | 6.17 | 9 | 3.08 | 9.0 | 1.16 | 9 |
| MAHABUBNAGAR | TELANGANA | 22.82 | 3 | 1.49 | 3 | 27.39 | 8 | 6.93 | 6 | 2.52 | 8.0 | 0.73 | 6 |
| MANCHERIAL | TELANGANA | 21.63 | 2 | 1.28 | 1 | 29.11 | 10 | 4.93 | 9 | 4.60 | 10.0 | 1.37 | 9 |
| MEDAK | TELANGANA | 21.72 | 2 | 1.31 | 2 | 27.11 | 10 | 5.08 | 10 | 4.59 | 10.0 | 1.74 | 10 |
| MEDCHAL-MALKAJGIRI | TELANGANA | 22.94 | 3 | 1.23 | 1 | 26.31 | 10 | 5.37 | 9 | 3.90 | 10.0 | 1.26 | 9 |
| NAGARKURNOOL | TELANGANA | 21.30 | 2 | 1.44 | 3 | 26.79 | 10 | 5.63 | 9 | 4.26 | 10.0 | 1.51 | 9 |
| NALGONDA | TELANGANA | 21.64 | 2 | 1.36 | 2 | 28.01 | 8 | 6.68 | 8 | 2.65 | 8.0 | 1.11 | 8 |
| NIRMAL | TELANGANA | 21.66 | 2 | 1.21 | 1 | 28.75 | 10 | 5.96 | 10 | 5.07 | 10.0 | 1.81 | 10 |
| NIZAMABAD | TELANGANA | 23.02 | 3 | 1.46 | 3 | 26.78 | 10 | 5.07 | 10 | 3.77 | 10.0 | 1.84 | 10 |
| PEDDAPALLI | TELANGANA | 21.59 | 2 | 1.18 | 1 | 34.96 | 10 | 6.11 | 10 | 5.59 | 10.0 | 3.66 | 10 |
| RAJANNA SIRCILLA | TELANGANA | 22.27 | 3 | 1.51 | 3 | 28.76 | 10 | 5.91 | 10 | 3.71 | 10.0 | 1.58 | 10 |
| RANGA REDDY | TELANGANA | 22.62 | 3 | 1.69 | 5 | 26.53 | 9 | 4.48 | 8 | 2.96 | 9.0 | 1.13 | 8 |
| SANGAREDDY | TELANGANA | 20.55 | 2 | 1.09 | 1 | 24.72 | 9 | 4.34 | 9 | 3.37 | 9.0 | 1.50 | 9 |
| SIDDIPET | TELANGANA | 20.74 | 2 | 1.23 | 1 | 30.66 | 10 | 5.81 | 10 | 4.71 | 10.0 | 1.86 | 10 |
| SURYAPET | TELANGANA | 23.01 | 3 | 1.54 | 4 | 28.55 | 10 | 5.98 | 9 | 3.88 | 10.0 | 1.29 | 9 |
| VIKARABAD | TELANGANA | 20.22 | 1 | 1.30 | 2 | 34.76 | 10 | 8.03 | 10 | 3.57 | 10.0 | 1.75 | 10 |
| WANAPARTHY | TELANGANA | 22.73 | 3 | 1.45 | 3 | 28.93 | 10 | 6.45 | 10 | 4.39 | 10.0 | 1.76 | 10 |
| WARANGAL RURAL | TELANGANA | 22.25 | 3 | 1.68 | 5 | 30.69 | 10 | 5.22 | 9 | 4.33 | 10.0 | 1.35 | 9 |
| WARANGAL URBAN | TELANGANA | 21.26 | 2 | 1.11 | 1 | 31.49 | 10 | 5.65 | 10 | 4.04 | 10.0 | 1.80 | 10 |
| YADADRI BHUVANAGIRI | TELANGANA | 21.81 | 2 | 1.34 | 2 | 26.93 | 10 | 4.53 | 10 | 4.03 | 10.0 | 1.68 | 10 |
| GOMATI | TRIPURA | 29.11 | 9 | 1.56 | 4 | 30.24 | 5 | 6.51 | 6 | 1.69 | 5.0 | 0.68 | 6 |
| KHOWAI | TRIPURA | 29.81 | 10 | 2.00 | 9 | 31.44 | 3 | 6.44 | 5 | 1.42 | 3.0 | 0.61 | 5 |
| NORTH TRIPURA | TRIPURA | 31.30 | 10 | 2.16 | 10 | 34.25 | 4 | 6.98 | 4 | 1.55 | 4.0 | 0.54 | 4 |
| SEPAHIJALA | TRIPURA | 30.70 | 10 | 1.74 | 6 | 34.62 | 6 | 6.67 | 5 | 1.98 | 6.0 | 0.63 | 5 |
| SOUTH TRIPURA | TRIPURA | 30.22 | 10 | 1.42 | 3 | 32.42 | 4 | 7.33 | 3 | 1.64 | 4.0 | 0.42 | 3 |
| UNAKOTI | TRIPURA | 28.78 | 9 | 1.68 | 5 | 34.84 | 8 | 7.00 | 9 | 2.69 | 8.0 | 1.43 | 9 |
| WEST TRIPURA | TRIPURA | 30.88 | 10 | 1.67 | 5 | 33.30 | 3 | 6.92 | 3 | 1.39 | 3.0 | 0.42 | 3 |
| AMETHI | UTTAR PRADESH | 25.33 | 6 | 1.88 | 8 | 18.04 | 3 | 4.44 | 2 | 1.35 | 3.0 | 0.35 | 2 |
| BUDAUN | UTTAR PRADESH | 23.98 | 5 | 1.52 | 4 | 27.70 | 5 | 6.80 | 7 | 1.79 | 5.0 | 0.81 | 7 |
| GHAZIABAD | UTTAR PRADESH | 24.89 | 6 | 1.51 | 3 | 23.60 | 7 | 4.56 | 9 | 2.12 | 7.0 | 1.23 | 9 |
| HAPUR | UTTAR PRADESH | 23.78 | 4 | 2.04 | 9 | 25.02 | 2 | 6.42 | 2 | 1.03 | 2.0 | 0.26 | 2 |
| MORADABAD | UTTAR PRADESH | 23.21 | 3 | 1.57 | 4 | 19.70 | 2 | 6.06 | 2 | 1.19 | 2.0 | 0.36 | 2 |
| MUZAFFARNAGAR | UTTAR PRADESH | 21.97 | 2 | 1.61 | 5 | 21.78 | 7 | 6.04 | 6 | 2.15 | 7.0 | 0.75 | 6 |
| RAE BARELI | UTTAR PRADESH | 23.25 | 4 | 1.62 | 5 | 21.95 | 6 | 5.57 | 6 | 1.95 | 6.0 | 0.71 | 6 |
| SAMBHAL | UTTAR PRADESH | 22.40 | 3 | 2.11 | 9 | 26.60 | 3 | 6.09 | 6 | 1.42 | 3.0 | 0.76 | 6 |
| SHAMLI | UTTAR PRADESH | 22.44 | 3 | 1.44 | 3 | 19.06 | 1 | 4.93 | 1 | 0.94 | 1.0 | 0.24 | 1 |
| SULTANPUR | UTTAR PRADESH | 24.80 | 6 | 1.90 | 8 | 20.84 | 5 | 5.68 | 5 | 1.67 | 5.0 | 0.59 | 5 |
| PASCHIM BARDDHAMAN | WEST BENGAL | 34.05 | 10 | 2.88 | 10 | 28.09 | 2 | 7.60 | 3 | 1.22 | 2.0 | 0.41 | 3 |
| PURBA BARDDHAMAN | WEST BENGAL | 27.88 | 8 | 1.52 | 4 | 47.22 | 6 | 6.96 | 5 | 1.89 | 6.0 | 0.63 | 5 |
